# Post graduate remediation programs in medicine: a scoping review

**DOI:** 10.1186/s12909-022-03278-x

**Published:** 2022-04-20

**Authors:** Clarissa Wei Shuen Cheong, Elaine Li Ying Quah, Keith Zi Yuan Chua, Wei Qiang Lim, Rachelle Qi En Toh, Christine Li Ling Chiang, Caleb Wei Hao Ng, Elijah Gin Lim, Yao Hao Teo, Cheryl Shumin Kow, Raveendran Vijayprasanth, Zhen Jonathan Liang, Yih Kiat Isac Tan, Javier Rui Ming Tan, Min Chiam, Alexia Sze Inn Lee, Yun Ting Ong, Annelissa Mien Chew Chin, Limin Wijaya, Warren Fong, Stephen Mason, Lalit Kumar Radha Krishna

**Affiliations:** 1 Yong Loo Lin School of Medicine, National University Singapore, 1E Kent Ridge Road, 119228 NUHS Tower Block, Level, Singapore, 11 Singapore; 2 Division of Supportive Palliative and Care, National Cancer Centre Singapore, 11 Hospital Crescent, Singapore, 16961 Singapore; 3 Division of Cancer Education, National Cancer Centre Singapore, 11 Hospital Crescent, Singapore, 169610 Singapore; 4 Medical Library, National University of Singapore Libraries, Blk MD6, Centre, 14 Medical Dr, #05-01 for Translational Medicine, Singapore, 117599 Singapore; 5 Duke-NUS Medical School, 8 College Road, Singapore, 169857 Singapore; 6 Department of Infectious Diseases, Singapore General Hospital, Outram Road, Singapore, 169608 Singapore; 7 Department of Rheumatology and Immunology, Singapore General Hospital, 16 College Road, Block 6 Level 9, Singapore, 169854 Singapore; 8 Palliative Care Institute Liverpool, Academic Palliative & End of Life Care Centre, Cancer Research Centre, University of Liverpool, 200 London Road, Liverpool, L3 9TA UK; 9 Centre for Biomedical Ethics, National University of Singapore, Blk MD11, 10 Medical Drive, #02-03, Singapore, 117597 Singapore; 10 PalC, The Palliative Care Centre for Excellence in Research and Education, PalC c/o Dover Park Hospice, 10 Jalan Tan Tock Seng, Singapore, 308436 Singapore

**Keywords:** Postgraduate physicians, Physicians in training, Remediation, Surgical, Medical, Education, Systematic scoping review

## Abstract

**Background:**

Recognizing that physicians may struggle to achieve knowledge, skills, attitudes and or conduct at one or more stages during their training has highlighted the importance of the ‘deliberate practice of improving performance through practising beyond one’s comfort level under guidance’. However, variations in physician, program, contextual and healthcare and educational systems complicate efforts to create a consistent approach to remediation.

Balancing the inevitable disparities in approaches and settings with the need for continuity and effective oversight of the remediation process, as well as the context and population specific nature of remediation, this review will scrutinise the remediation of physicians in training to better guide the design, structuring and oversight of new remediation programs.

**Methods:**

Krishna’s Systematic Evidence Based Approach is adopted to guide this Systematic Scoping Review (SSR in SEBA) to enhance the transparency and reproducibility of this review. A structured search for articles on remediation programs for licenced physicians who have completed their pre-registration postings and who are in training positions published between 1st January 1990 and 31st December 2021 in PubMed, Scopus, ERIC, Google Scholar, PsycINFO, ASSIA, HMIC, DARE and Web of Science databases was carried out. The included articles were concurrently thematically and content analysed using SEBA’s Split Approach. Similarities in the identified themes and categories were combined in the Jigsaw Perspective and compared with the tabulated summaries of included articles in the Funnelling Process to create the domains that will guide discussions.

**Results:**

The research team retrieved 5512 abstracts, reviewed 304 full-text articles and included 101 articles. The domains identified were characteristics, indications, frameworks, domains, enablers and barriers and unique features of remediation in licenced physicians in training programs.

**Conclusion:**

Building upon our findings and guided by Hauer et al. approach to remediation and Taylor and Hamdy’s Multi-theories Model, we proffer a theoretically grounded 7-stage evidence-based remediation framework to enhance understanding of remediation in licenced physicians in training programs. We believe this framework can guide program design and reframe remediation’s role as an integral part of training programs and a source of support and professional, academic, research, interprofessional and personal development.

**Supplementary Information:**

The online version contains supplementary material available at 10.1186/s12909-022-03278-x.

## Background

Remediation is increasingly seen as an integral part of all training programs, yet their incorporation in current training programs face a number of hurdles [1]. To begin, remedial interventions may be called upon to support a variety of gaps in medical knowledge, clinical and communication skills and/or professional principles [2–4]. Remediation may also involve addressing attitudinal, behavioural and or motivational issues at more than one time point in the training journey. Critically, remediation of a number of aspects may be called for. This varied and personalised approach to remediation is unsurprising given the particularities of the speciality, its setting, regnant sociocultural, organizational, curriculum, healthcare, educational, academic, research, and clinical influences, the influence of the hidden, formal and informal curriculum [5–8]. Remediation is also influenced by the physician’s demographical and historical perspectives, experiences, motivations, insights, willingness to engage and openness to learning, and their training backgrounds, experiences, level of competence and skills in the clinical, academic, and research spheres [9, 10].

It is thus unsurprising that given the need for flexible, systems-based, speciality-sensitive, context-specific interventions to prepare physicians for their many responsibilities and roles [11]; a robust, systematic and transparent approach to remediation continues to evade practice [12]. However with variability in current “*formal programs intended to assist residents”,* (or by extension, doctors) *“in difficulty, by facilitating a correction for those who are struggling to achieve competency in their discipline”* remediation processes are increasingly prone to compromises in the manner that personalised remediation is provided [12].

### Current reviews of remediation

Acknowledging these gaps and concerns, a number of reviews on remediation in the postgraduate setting were carried out. Recognising the context specificity and population dependent nature of remediation processes, most reviews and commentaries have moved away from combined reviews of remediation seen in Lacasse et al. (2019)‘s BEME review, Bourgeois-Law et al. (2018)‘s scoping review, Al-Sheikly et al. (2020)‘s review of remediation of communication skills, Brennan et al. (2020) review of remediation of professionalism lapses in medical students and doctors [13], Kalet et al. (2017)‘s ‘eye opener’ to remediation [14], Ellaway et al. (2018)‘s commentary of remediation of competency-based medical education, Chou et al. (2019)‘s dos and don’ts of remediation in medical education, similarly included medical students and postgraduates physicians in training in diverse settings [15]. Unsurprisingly Shearer et al. (2019) and Kebaetse et al. (2018) found that many remediation programs did not align with best practice guidelines [9, 16]. Yet these reviews such as Hauer et al’s 2009 broad review of remediation in undergraduate, graduate and continuing medical education remain influential forming the basis for focused reviews and guidance to general design of remediation programs.

For our purposes of designing a remediation program for licenced physicians in training programs focused reviews such as Morris et al. (2012)‘s and Barrett et al. (2016)‘s BEME review have helped addressed some of the gaps in how remediation programs should be designed, structured and assessed; yet many gaps remain [17].

### Need for this review

Guided by Bourgeois-Law et al. (2018)‘s scoping review of remediation in practicing physicians [1], Kurzweil and Galleta’s (2018) commentary of neurology residents [18] and latterly by Price et al’s (2021) review of remediation for doctors and Pirie et al’s (2020) review of remediation amongst residents in competency based residency educational systems, a holistic understanding of the remediation process for licenced physicians in training such as residency, advanced residency and fellowship programs remain unclear particularly when few account for the learning environment in remediation [19]. To achieve a more holistic review we adopt a systematic scoping review (SSR) and build on Pirie et al. (2020)‘s and Qi et al. (2021)‘s limited reviews of remediation of ‘residents in difficulty’. We propose to analyse current literature on postgraduate remediation programs to better inform future programs for physicians in training [20–22]. It is our intention to design, structure and assess our remediation program based upon these findings, and accounting for Cleland et al. (2021)‘s conclusions on the impact of the learning environment on the remediation process.

## Methods

Whilst SSRs are well suited to contend with the personalised and sociocultural elements of the remediation processes given their Constructivist ontological and Relativist epistemological roots, [23–26] we adopt Krishna’s Systematic Evidence Based Approach (henceforth SEBA) to overcome concerns over their consistency, reproducibility and transparency [27–32]. With SEBA methodology evidenced in reviews of teaching and assessing mentoring, communication, empathy, ethics, professionalism training and portfolio use [33–41], we believe it will be well suited to guide this review.

**Fig. 1:**
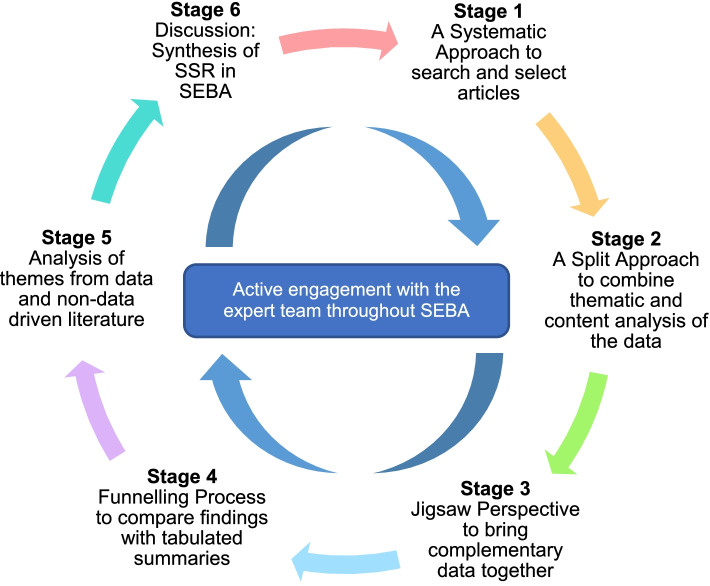
The SEBA process

Our SEBA guided SSR (henceforth SSR in SEBA) of remediation programs for licenced physicians in training programs will include SEBA’s 6-stage process offering a systematic approach to searching and selecting articles for the review, Split Approach, The Jigsaw Perspective, The Funnelling Process, analysis of the data and non-data driven literature and synthesis of the discussion focuses on enhancing consistency, reproducibility, and transparency in the structured research process (Fig. 1). We will discuss each of these aspects in the coming sections. The principles of interpretivist analysis are also employed to enhance reflexivity and discussions in SEBA’s six stages.

SEBA relies on an expert team composed of medical librarians from the Yong Loo Lin School of Medicine (YLLSoM) at the National University of Singapore (NUS) and local educational experts and clinicians at the National Cancer Centre Singapore (NCCS), the Palliative Care Institute Liverpool, YLLSoM and Duke-NUS Medical School (henceforth expert team) to guide, oversee and support its 6 stages. Employ of the expert team is key given that this review is being carried out as part of an educational initiative run by the Division of Supportive and Palliative Care and the Division of Cancer Education at NCCS called the Palliative Medicine Initiative (PMI) *[*32*]*. The PMI aims at boost interests in Palliative and Supportive Care, medical education, ethics and professionalism amongst medical students and junior doctors using a Novice Mentoring approach. This approach sees the medical students and junior doctors involved in this review mentored in small groups of two or three mentees through each stage of the review process.

### Stage 1: Systematic Approach

i. Determining the title and background of the review

The research and expert teams set out the overarching goals, study population, context and remediation programs to be evaluated. Based on the findings of recent reviews of remediation and Price et al. (2021)‘s realist review of remediation that underscores the impact of the remediation environment and structure, the expert team underscored the need to be mindful of the differences in assessments, follow up, feedback, remediation and follow up present in training programs in postgraduate medicine and those available to physicians who are not in, have exited or who have completed their training. As a result we focus on licenced physicians in formal training programs and recognise that the stage of the physician’s career has significant bearing upon the remediation process [42]. As a result we do not include attendings, consultants and physicians who are not in training programs.

However, recognising that most training programs are subject to similar competencies as exemplified by surgical, medical, psychiatry, obstetrics and gynaecology and paediatrics residency programs under the Accreditation Council for Graduate Medical Education (ACGME) [43], the Academy of Royal Colleges [44], The Royal Colleges of Physicians and Surgeons of Canada [45], The Royal Australasian College of Surgeons [46] or The Royal Australasian College of Physicians [47], we studied remediation programs in all training programs involving licenced physicians.

Acknowledging the complexity of the remediation process and the manner that it is carried out and supported, we adopt Price et al. (2021)‘s definition of remediation as “an intervention, or suite of interventions, required in response to assessment against threshold standards’ with the aim of remedying underperformance so the doctor can return to safe practice”.

ii. Identifying the research question

To this end guided by the Population, Intervention, Comparison, Outcome and Study Design (PICOS) elements of the inclusion criteria [48, 49], the primary research question focuses on remediation programs in formal medical training programs which include all specialities and subspecialities of psychiatry, medicine, surgery, paediatrics, family medicine and obstetrics and gynaecology. These must be formal training programs or structured and assessed longitudinal programs involving fully registered or licenced physicians. These include residency and advanced training programs, specialist training, surgical training, and other speciality and subspeciality training programs.

The primary research question was determined to be: “*What is known of remediation programs in training programs for licenced physicians?*” The secondary research questions were “*What methods are used to structure remediation programs in training programs for licenced physicians?*” and “*what are the characteristics of remediation programs in training programs for licenced physicians?*”

iii. Inclusion criteria

Guided by the expert team, the research team created the inclusion criteria for the SSR in SEBA as outlined (Table 1).

**Table 1:** PICOS

PICOs	Inclusion Criteria	Exclusion Criteria
Population	• Licenced physicians in training. These are doctors who have graduated from medical school and completed their pre-registration postings and are fully licenced physicians and who are in training positions.	• Specialists who have completed their training • Physicians who have completed or who have left training programs • Physicians who are not in training schemes or programs • Medical Students • Allied health specialties such as Pharmacy, Dietetics, Chiropractic, Midwifery, Podiatry, Speech Therapy, Occupational and Physiotherapy • Non-medical specialties such as Clinical and Translational Science, Alternative and Traditional Medicine, Veterinary, Dentistry
Intervention	• Remediation programmes in the academic, professional and clinical context as part of a training program in any field of medicine	• Poor characterisation of remediation processes.
Comparison	• Comparisons of the various practices in remediation programmes (approaches, modalities, processes, objectives, motivations, challenges, facilitating characteristics/resources)	
Outcome	• Impact of remediation programmes on host organisation and other relevant stakeholders. • Evaluation of remediation processes by institutions	
Study design	• Articles in English or translated to English • All study designs including mixed methods research, meta-analyses, systematic reviews, randomized controlled trials, cohort studies, case-control studies, cross-sectional studies, descriptive papers, opinion pieces and grey literature • Years of Publication: 1 January 1990–31 December 2021 • Databases: PubMed, SCOPUS, Web of Science, ERIC, Google Scholar, ASSIA, DARE, PsycINFO

iv. Searching

Ten members of the research team carried out independent searches of PubMed, Scopus, ERIC, Google Scholar, Psycinfo, ASSIA, HMIC, DARE and Web of Science databases. To facilitate this process the search process saw three senior researchers well versed in carrying out systematic reviews and systematic scoping reviews each met with the one team of 2–3 medical students and guided them through the search process of four databases. This approach was to train new researchers in the PMI and to ensure that at least two teams were independently reviewing each database. Each team met regularly and discussed their findings. After a search of the first 100 articles in a particular database, the medical students and the mentor who was the senior researcher compared their findings at an online meeting. Subsequently the teams met at specific time points often after reviewing a predetermined number of included articles to discuss their concerns, exchange opinions and advance their understanding of the research process and the area of study. Interrater reliability was not evaluated.

To ensure a sustainable research process, the research team confined the searches to articles published between 1st January 1990 and 31st December 2021 to account for prevailing manpower and time constraints. The independent searches, hand searching of seven leading journals in medical education (Academic Medicine, Medical Education, Medical Teacher, Advances Health Sciences Education, BMC Medical Education, Teaching and Learning in Medicine and Perspectives on Medical Education) and ancestry searches were conducted between 12th September 2020 and 18th October 2020 and repeated between 14th February 2021 and 18th April 2021. The PubMed search strategy may be found in the Appendix.

v. Extracting and charting.

The ten members of the research team reviewed all the titles and abstracts identified, created individual lists of titles to be included and discussed these online within their teams. Working in teams of three medical student and a senior reviewer, the teams reviewed the abstracts and titles and discussed their findings at regular meetings. The findings of the 3 teams were then discussed at online meetings where Sandelowski and Barroso [50]‘s ‘negotiated consensual validation’ was used to achieve consensus on the final list of titles to be reviewed. Here, ‘negotiated consensual validation’ refers to

*“a social process and goal, especially relevant to collaborative, methodological, and integration research, whereby research team members articulate, defend, and persuade others of the “cogency” or “incisiveness” of their points of view or show their willingness to abandon views that are no longer tenable. The essence of negotiated validity is consensus”*. (p.229)

The three research teams repeated this process independently studying all the full text articles on the final list of titles, created their own lists of articles to be included and discussed their findings online at research meetings. ‘Negotiated consensual validation’ was used to achieve consensus on the final list of articles to be analysed.

### Stage 2 of SEBA: Split Approach

To enhance validity of the analysis, a split approach is employed with concurrent use of thematic and directed content analysis. Three teams of researchers simultaneously and independently reviewed the included full-text articles. The first team summarised and tabulated the included full-text articles in keeping with recommendations drawn from Wong, Greenhalgh [51]‘s RAMESES publication standards: meta-narrative reviews and Popay, Roberts [52]‘s “Guidance on the conduct of narrative synthesis in systematic reviews”. The tabulated summaries served to ensure that key aspects of included articles were not lost.

Concurrently, the second team analysed the included articles using Braun and Clarke [53]‘s approach to thematic analysis. In phase 1, the research team carried out independent reviews, ‘actively’ reading the included articles to find meaning and patterns in the data. In phase 2, ‘codes’ were constructed from the ‘surface’ meaning and collated into a code book to code and analyse the rest of the articles using an iterative step-by-step process. As new codes emerged, these were associated with previous codes and concepts. In phase 3, the categories were organised into themes that best depict the data. An inductive approach allowed themes to be “*defined from the raw data without any predetermined classification*” [54]. In phase 4, the themes were refined to best represent the whole data set and discussed. In phase 5, the research team discussed the results of their independent analysis online and at reviewer meetings. ‘Negotiated consensual validation’ was used to determine a final list of themes approach and ensure the final themes.

A third team of researchers employed Hsieh and Shannon [55]‘s approach to directed content analysis to analyse the 107 included articles. Analysis using the directed content analysis approach involved “*identifying and operationalizing a priori coding categories*”. The first stage saw the research team draw categories from both Hauer et al. (2009)‘s article “Remediating Professionalism Lapses in Medical Students and Doctors: A Systematic Review” and the locally employed ACGME core competencies [43] to guide the coding of the articles in the second stage. Any data not captured by these codes were assigned a new code. In keeping with deductive category application, coding categories were reviewed and revised as required.

In the third stage, the research team discussed their findings online and used ‘negotiated consensual validation’ to achieve consensus. The final codes were compared and discussed with the final author, who checked the primary data sources to ensure that the codes made sense and were consistently employed. Any differences in coding were resolved between the research team and the final author. ‘Negotiated consensual validation’ was used as a means of peer debrief in all three teams to further enhance the validity of the findings.

#### Quality assessment of studies

To enhance methodological rigour and to provide reviewers with a chance to evaluate the credibility of the conclusions and the transferability of the findings the six members of the research team independently reviewed all the articles on the final list based on the Medical Education Research Study Quality Instrument (MERSQI) [56] and the Consolidated Criteria for Reporting Qualitative Studies (COREQ) [57] quality assessments (Appendix). We also added the JBI Critical Appraisal Checklist for Systematic Reviews [58] following the reviewers’ comments.

## Results

**Fig. 2:**
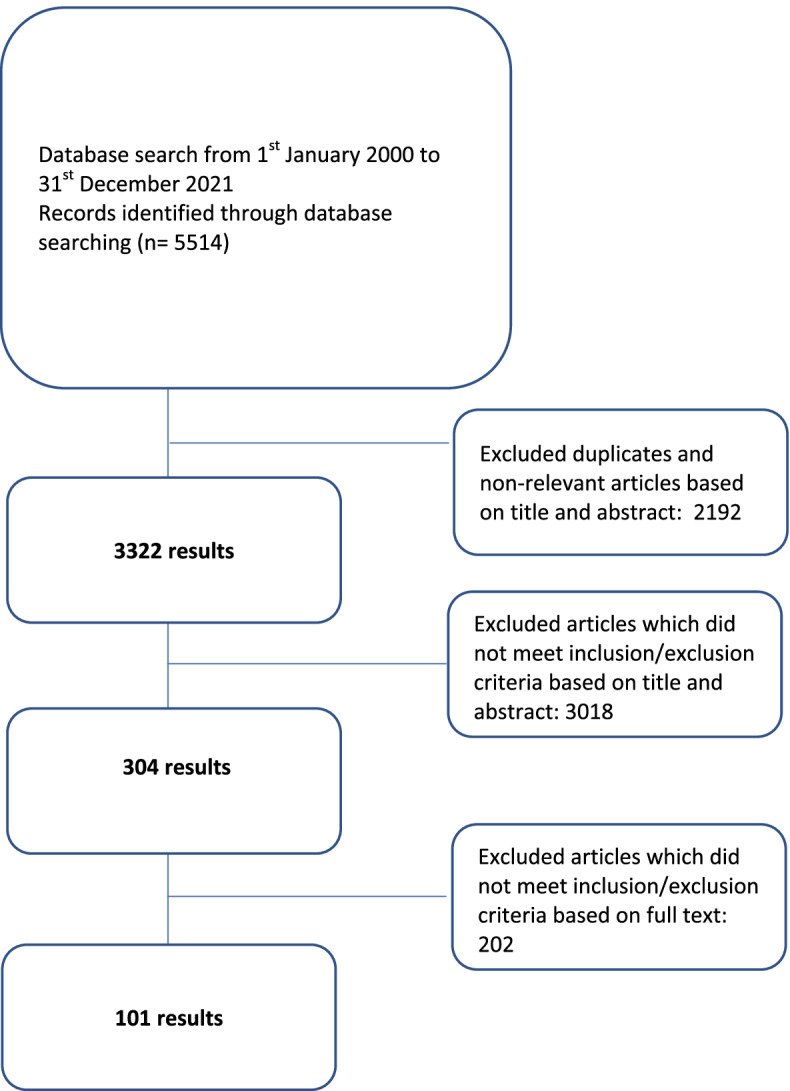
PRISMA Flowchart

A total of 5514 abstracts were identified, 304 full-text articles were reviewed, 101 articles were included as shown in Fig. 2 below.

The themes identified through thematic analysis were

- Characterising Remediation
- Reasons for Remediation
- Frameworks for Remediation
- Modalities for Remediation
- Outcome Measures of Remediation
- Enablers and Barriers of Remediation
- Unique Features of Postgraduate Remediation

The categories identified through directed content analysis were

- Domains of Remediation
  - Medical Knowledge
  - Practice Based Learning and Improvement
  - Patient Care and Procedural Skills
  - Interpersonal and Communication Skills
  - Professionalism
- Reasons for Remediation
- Modalities for Remediation
- Outcome Measures of Remediation
- Enablers and Barriers of Remediation

### Stage 3 of SEBA: Jigsaw Perspective

In keeping with SEBA’s reiterative process, the themes and categories were reviewed by the expert and research teams. Here, overlaps between the categories and themes were viewed as pieces of a jigsaw puzzle with the intention of combining overlapping/complementary pieces to create a bigger piece of the puzzle referred to as themes/categories.

To create themes/categories the Jigsaw Perspective adopted Phases 4 to 6 of France, Uny [59]‘s adaptation of Noblit, Hare [60]‘s seven phases of meta-ethnography. As per Phase 4, the themes and the categories identified in the Split Approach are grouped according to their focus. These groups of categories and themes are contextualized by reviewing the articles from which the themes and categories were drawn from. Reciprocal translation was then used to determine if the themes and categories can be used interchangeably.

The themes/categories delineated included the goals of remediation, approaches, practice-based learning and improvement, patient care and procedural skills, interpersonal communication skills, professionalism and knowledge.

### Stage 4 of SEBA: Funnelling Process

The Funnelling Process employed Phases 3 to 5. To begin, the themes/categories identified in the Jigsaw Approach were juxtaposed with the key messages identified in the tabulated summaries to create *funnelled domains*. The process saw the goals, approaches and assessment themes combined within the categories of patient care and procedural skills, interpersonal communication skills, professionalism, knowledge and enablers and barriers. These *domains* included characterization, reasons, frameworks, and enablers and barriers. These *domains* formed the basis for ‘the line of argument’ in Stage 6 of SEBA.

### Domains

I. Characterising remediation

Remediation is characterised as an organised and targeted process focused upon specified and agreed upon targets [61, 62] and supporting learners at risk of “falling from good standing” [63], with difficulty [9, 18, 62, 64–80] or with identified deficiencies [74, 81] in content knowledge, skills, attitudes, clinical reasoning [82, 83] and or professionalism [84]. Remediation is also a “regulatory process removing an individual’s educational autonomy” [80] that also sees close follow-up by faculty [62, 85, 86].

Magin, Stewart [63], Parran, Pisman [69] distance remedial action from sanctions, probation or as a form of punishment that carries the threat of termination. Rather these authors frame remediation as a form of “physician enhancement” [87] aimed at assisting learners to develop a deeper understanding of their professional identity formation and their obligation to professional development [88].

Remediation processes are either seen as subsidiary to [63, 78, 79, 89–91] or separate from the standard curricula [79, 92]. Remediation is rarely seen as “part of an educational continuum involving different degrees of support” within the formal curricula [80].

II. Reasons for Remediation

Personal factors contributing to the need for remediation include:

- mental health concerns including anxiety, depression and stress [6, 11, 65, 66, 71, 74, 85, 93–102],
- poor physical health [74, 97, 99]
- learning disability or difficulties (e.g. conceptualisation, time management or assessment anxiety) [6, 65, 85, 93, 96, 98, 100, 102–104]
- substance abuse [65, 66, 68, 85, 93, 99, 105, 106]
- personality disorders [88, 95]
- personal relationship concerns such as divorce [65, 66, 74, 94, 101]
- lack of resilience, and setbacks that threaten one’s identity [92, 106]

Remediation may also be indicated for concerning changes in behaviour [93], lapses in professionalism [98, 105–109], a lack of motivation knowledge gaps, skills shortfalls or attitude shortcomings [61, 68, 71, 72, 75, 82, 89, 95, 98, 101, 102, 105, 109–116].

Personal factors are more likely to require remediation when learners are older [102, 117, 118], had reported character failings [119], required remediation in the past [120], learners who required more time to complete medical school [121], had more re-examinations in the past [121] and previous lapses in professionalism in medical school [92].

Program related factors predisposing to professional lapses and the need for remediation [66] include high workload [84, 112, 118], chronic fatigue, sleep deprivation, stress and overwork [84], a “culture of recurrent assessment and unsupportive teaching environments” [74] and changing placements which requires learners to adapt to new environments and form new relationships [74].

III. Frameworks for Remediation

The ACGME, CanMEDs, General Medical Council (GMC), American Board of Surgery In-Training Examination (ABSITE), Collége des Médecins du Québec (CMQ) and American Board of Pediatrics In-Training Examination (ABP ITE) frameworks have been variously used to detect physicians in need of remediation. When considered alongside the physician’s particular psychosocial, clinical, professional, research and academic situation these frameworks have been used to diagnose specific limitations, shape personalised practical remediation processes, inform career readjustment, improve patient safety and care and even minimize personal and financial costs for the struggling physician [96, 122] (Table 2).

**Table 2:** Frameworks for Methods of Identification

Framework	Description	References
ACGME	The Accreditation Council on Graduate Medical Education (ACGME) and the American Board of Medical Specialties created the Milestones Project to provide competency-based outcomes for trainees. Milestones serve many purposes in both graduate medical education and the accreditation process. Among them, milestones provide transparent expectations, support better longitudinal assessment of trainees, and enhance public accountability through aggregate reporting of competency by specialty [123].	[18, 62, 64, 65, 77, 78, 81, 85, 89, 90, 93, 99, 105, 114, 124–128]
CanMeds	CanMEDS is a framework that delineates the outcomes that doctors should achieve to effectively meet the healthcare needs of the people they serve. These abilities are grouped thematically under seven roles. A competent physician seamlessly integrates the competencies of all seven CanMEDS Roles. The CanMEDS Roles • Medical Expert (the integrating role) • Communicator • Collaborator • Leader • Health Advocate • Scholar • Professional The overarching goal of CanMEDS is to improve patient care. The CanMEDS model has been adapted around the world, both within and outside the health professions.	[9, 88, 107]
GMC	The General Medical Council (GMC) sets the standards expected of medical training organisations and outcomes that doctors in training practising in the UK should achieve.	[101, 112, 129, 130]
ABSITE	The American Board of Surgery In-Training Exam (ABSITE) has been offered annually to surgical residents training in accredited programs in the United States since 1975. The examination consists of 225 multiple-choice questions and must be completed in 5 h. The results are defined as the percent correct, standard score, and percentile [compared with other examinees in the same postgraduate year (PGY)] and are reported for the total test and the basic science and clinical management portions of the examination to the program directors	[95, 131–133]
CMQ	The CMQ identifies physicians with clinical performance problems primarily through the professional inspection committee, complaints forwarded to the inquiry division, or processes initiated by physicians who would like to reorient their careers or come back to practice after a period of inactivity of over 4 years [110].	[110]
ABP ITE	The American Board of Paediatrics (ABP) has offered the In-Training Examination (ITE) annually since 1971 to pediatric trainees in US and Canadian programs as a service to residents and program directors. The ITE is a 3-h exam consisting of approximately 150 multiple-choice questions and is administered on designated days in July.	[86]

This holistic perspective is particularly useful in addressing issues such as alcohol abuse, rehabilitation, and resolution of personal issues [9, 87, 97, 130, 134].

IV. Domains for Remediation
  **A.** Detecting and confirmation

i. Medical Knowledge

Gaps in knowledge are detected through regular in-training assessments. This may involve standardised tests or clinical assessments [9, 61, 64, 65, 87, 94, 95, 99, 110, 115, 128, 130–133, 135, 136], simulations [18, 78], retrospective record reviews [96], Objective Structured Clinical Examinations (OSCE)s [70, 87, 96, 113, 137, 138], and or failure to meet recertification requirements such as entrustable professional activities (EPAs) [72, 96]. These shortfalls should be considered in tandem with the supervisor’s clinical evaluations [62, 65], interviews [96, 139], peer ratings [65, 96], self-assessments [128], neuropsychological testing [140] and or activity logs [137].

ii. Practice-Based Learning and Improvement

Practice-Based Learning and Improvement entails the ability to comprehend relevant information and a commitment to lifelong learning [43]. Gaps in this domain may be identified through regular in-training assessments such as standardised tests or clinical assessments [9, 61, 64, 65, 87, 94, 95, 110, 115, 128, 130, 131, 133, 135, 136], simulations [18, 78], retrospective record reviews [96], OSCEs [70, 87, 96, 113, 137, 138], failure to achieve EPAs [72, 96]. These findings should be considered in tandem with supervisory evaluations [62, 65, 103], interviews [96, 139], peer ratings [65, 96], self and activity logs [137].

iii. Patient Care and Procedural Skills

Objective methods of identifying lapses in patient care and gaps in procedural skills include regular in-training assessments [64, 65, 95, 110, 115, 128, 130, 133, 135, 136], simulations [18, 78], OSCEs [70, 96, 137, 138], failure to achieve EPAs [72]. These findings should be considered with supervisory or clinical evaluations [62, 65, 120], self-assessments [128], and activity logs [137].

iv. Interpersonal and Communication Skills

The need for remediation in a physician’s interpersonal and communication skills may be identified through in-training evaluations [137], OSCEs or oral examinations [137], simulated patients (SP) interactions [74, 84], team feedback and self-referrals [84]. Greater information may be accrued through supervisory or clinical evaluations [62, 65, 120], self-assessments [128], and activity logs [137].

v. Professionalism

The characterisation of unprofessional conduct set out by the American Board of Internal Medicine Examiners includes abuse of power, greed, arrogance, misrepresentation, impairment, lack of conscientiousness, and conflict of interest. Identifying lapses in professionalism pivots on the employ of longitudinal and holistic evaluations and continued learner engagement and feedback [114].

Unprofessional conduct can be assessed through online reporting system [116], formal resident evaluation systems [114], committee identification [92, 104], SP interactions [74, 84] and self-referral [84]. These findings may be corroborated by psychiatric assessment [87] and personality surveys [74].

B. Approach

i. Medical Knowledge

Remediation of knowledge deficits are summarised in Table 3.

**Table 3:** Modalities for Remediating Knowledge

Modality	References
Case discussions	[3, 68, 69, 72, 83, 86, 87, 110, 113, 137]
Clinical tutorials, workshops (Didactic and Educational Activity)	[3, 69, 86, 95, 96, 99, 110, 128, 140, 141]
Lectures	[67, 69, 70, 83]
Online courses, assignments, quizzes	[82, 132, 133, 138]
Repeat/increased clinical rotations	[18, 66, 77, 89, 128, 130, 132, 136]
Readings	[3, 65–67, 86, 89, 110, 128, 131, 137]

ii. Practice-Based Learning and Improvement

Remediation of Practice-Based Learning and Improvement are set out in Table 4.

**Table 4:** Modalities for Remediating Practice-Based Learning and Improvement

Modality	References
Intensified direct supervision	[65–67, 72, 77, 82, 87, 95, 96, 99–101, 105, 110, 113, 128, 130, 137, 142]
Role-modelling	[114]
Direct observation & feedback	[9, 62, 68, 69, 72, 85, 93, 98–100, 105, 110, 113, 122, 141, 143],
Simulations of clinical scenarios	[9, 62, 67, 78, 87, 98, 99, 125, 130, 138, 140, 141]
Case discussions	[68, 69, 72, 82, 83, 86, 87, 110, 113, 137, 144]
Clinical tutorials, workshops (Didactic and Educational Activity)	[69, 86, 95, 96, 99, 110, 128, 140, 141, 145]
Lectures	[67, 69, 70, 83]
Counselling	[74, 89, 95, 99, 100, 122]
Clinical embedding such as ward round presentations	[140]
Online courses, assignments, quizzes	[82, 132, 133, 138]
Repeat/increased clinical rotations	[18, 66, 77, 89, 99, 128, 130, 132, 136]
Readings	[65–67, 86, 89, 110, 128, 131, 137]
Holistic support (e.g. alcohol addiction rehabilitation, underlying reasons for substandard skills)	[9, 87, 97, 130, 134]

iii. Patient Care and Procedural Skills

Remediating lapses in patient care and gaps in procedural skills may involve a variety of techniques (Table 5).

iv. Interpersonal and Communication Skills

**Table 5:** Modalities for Remediating Patient Care and Procedural Skills

Modality	References
Intensified direct supervision	[65–67, 72, 77, 87, 99, 105, 113, 128, 130, 142]
Professional coaching to correct personal behaviour	[61, 72, 89, 99, 105, 145]
Direct observation & feedback	[72, 98, 99, 105, 113, 122, 141],
Simulations of clinical scenarios	[61, 67, 74, 78, 87, 98, 125, 130, 140, 141]
Clinical tutorials, workshops (Didactic and Educational Activity)	[112]
Lectures	[67]
Repeat/increased clinical rotations	[18, 66, 77, 89, 128, 130, 136]
Readings	[61, 65–67, 89, 128]
Holistic support (e.g. alcohol addiction rehabilitation, underlying reasons for substandard skills)	[9, 87, 97, 130, 134]

Modalities used in remediating a physician’s interpersonal and communication skills are outlined in Table 6.

v. Professionalism

**Table 6:** Modalities for Remediating Interpersonal and Communication Skills

Modality	References
Intensified direct supervision	[65–67, 72, 77, 85, 87, 95, 98–100, 105, 108, 113, 122, 128, 130, 132, 137, 141–143]
Professional coaching to correct personal behaviour	[72, 89, 95, 99, 100, 105, 122, 137, 145]
Simulations of clinical scenarios	[67, 74, 78, 87, 98, 99, 125, 130, 140, 141]
Case discussions	[72, 86, 87, 113, 137]
Clinical tutorials, workshops (Didactic and Educational Activity)	[86, 95, 99, 112, 128, 140, 141, 145]
Lectures	[67]
Counselling	[74, 89, 95, 99, 122]
Clinical embedding such as ward round presentations	[140]
Online courses, assignments, quizzes	[132, 133]
Repeat/increased clinical rotations	[18, 66, 77, 89, 99, 128, 130, 132, 136]
Readings	[65–67, 86, 89, 128, 131, 137]
Holistic support (e.g. alcohol addiction rehabilitation, underlying reasons for substandard skills)	[9, 87, 97, 130, 134]

A range of modalities have been utilised to address professionalism lapses. These are summarised in Table 7.

C. Outcome measures

1. Medical Knowledge and Practice-Based learning and improvement

**Table 7:** Modalities for Remediating Professionalism

Modality	References
Intensified direct supervision	[9, 62, 65–68, 72, 77, 85, 87, 95, 98, 99, 105, 113, 114, 122, 128, 130, 132, 137, 139, 141–143]
Professional coaching to correct personal behaviour	[72, 89, 95, 99, 105, 114, 122, 137, 139, 145]
Role-modelling	[108, 114]
Simulations of clinical scenarios	[9, 61, 62, 67, 74, 78, 87, 88, 98, 99, 125, 130, 138–141]
Case discussions	[72, 82, 86–88, 113, 137, 144]
Clinical tutorials, workshops (Didactic and Educational Activity)	[95, 99, 112, 128, 140, 141, 145]
Lectures	[67]
Counselling	[74, 89, 95, 99, 114, 122]
Clinical embedding such as ward round presentations	[140]
Online courses, assignments, quizzes	[82, 132]
Repeat/increased clinical rotations	[18, 66, 77, 89, 99, 128, 130, 132, 136]
Readings	[65–67, 89, 128, 137]
Holistic support (e.g. alcohol addiction rehabilitation, underlying reasons for substandard skills)	[87, 97, 98, 104, 106, 130]

There are a variety of outcome measures employed in remediation programs. Similarities in presented outcomes for medical knowledge and practice based learning allow both domains to be presented in Table 8.

2. Patient Care and Procedural Skills

**Table 8:** Outcome Measures for Remediating Knowledge

Outcome measure	References
Timing of evaluation	
Post-remediation evaluations	[62, 63, 72, 78, 81, 102, 110, 123, 131, 136, 137]
Mid-term evaluation	[62, 115, 137]
Quarterly assessment	[122]
Daily assessment	[9, 72, 93]
Monthly assessment	[95, 96, 105]
Nature of Assessments	
Longitudinal	[78, 96, 101, 113, 120, 125, 130, 133, 146]
Multisource	[9]
Type of Assessments	
Interview, oral exam	[62, 76, 87, 96, 101, 137]
Multiple Choice Questions	[69, 87, 147]
Objective Structured Clinical Examination	[18, 85, 87, 138, 147]
Patient records, documentation	[91, 145]
Workplace-based assessment, complaints	[85, 95, 96, 113, 135]

Outcome measures are as follows in Table 9.

3. Interpersonal and Communication Skills

**Table 9:** Outcome Measures for Remediating Patient Care and Procedural Skills

Outcome measure	References
Timing of evaluation	
Post-remediation evaluations	[72, 78, 81, 85, 123, 136, 137, 145]
Mid-term evaluation	[115, 137]
Quarterly assessment	[122]
Daily assessment	[72]
Monthly assessment	[95, 105]
Nature of Assessments	
Longitudinal	[74, 78, 113, 125, 130]
Type of Assessments	
Self-reflection such as reflective essay assignments and reports	[109, 130, 137]
Peer-assessment	[145]
Objective Structured Clinical Examination	[18, 85, 87, 120, 147]
Patient records, documentation	[91, 145]
Workplace-based assessment, complaints	[85, 95, 113, 135]

Outcome measures are as follows in Table 10.

4. Professionalism

**Table 10:** Outcome Measures for Remediating Interpersonal and Communication Skills

Outcome measure	References
Timing of evaluation	
Post-remediation evaluations	[63, 72, 78, 81, 85, 123, 136, 137, 145]
Mid-term evaluation	[115, 137]
Quarterly assessment	[122]
Daily assessment	[72]
Monthly assessment	[95, 105]
Nature of Assessments	
Longitudinal	[74, 78, 113, 125, 130, 133]
Type of Assessments	
Group discussions	[72]
Interview, oral exam	[62, 87, 137]
Self-reflection such as reflective essay assignments and reports	[109, 130, 137]
Peer-assessment	[145]
Objective Structured Clinical Examination	[18, 85, 87, 147]
Patient logs, records, documentation	[91, 145]
Workplace-based assessment, complaints	[85, 95, 113, 135]

Table 11 lists the various outcome measures utilised in remediating professionalism.

V. Enablers and Barriers

**Table 11:** Outcome Measures for Remediating Professionalism

Outcome measure	References
Timing of evaluation	
Post-remediation evaluations	[62, 72, 78, 81, 85, 123, 136, 137, 145]
Mid-term evaluation	[62, 115, 137]
Quarterly assessment	[122]
Daily assessment	[72]
Monthly assessment	[95, 105]
Nature of Assessments	
Longitudinal	[74, 78, 101, 113, 125, 130]
Multisource	[108]
Type of Assessments	
Group discussions	[72]
Interview, oral exam	[62, 87, 137]
Self-reflection such as reflective essay assignments and reports	[88, 109, 130, 137, 139]
Peer-assessment	[62, 127, 145]
Multiple Choice Questions	[69, 87, 147]
Objective Structured Clinical Examination	[18, 85, 87, 147]
Survey	[71, 144]
Workplace-based assessment, complaints, patient records and documentation	[85, 91, 95, 108, 113, 135, 145]

There are a variety of enablers and barriers of remediation processes and programs. These have been categorised to institutional, tutor and learner factors and are summarised in Table 12.

**Table 12:** Enablers to Successful Remediation Programs

Enablers	References
**Institutional factors**	
Remediation coordinator for streamlining of processes and outcomes	[9, 72, 89, 91, 92, 96, 100, 105, 110, 122, 123]
Screening for genuine shortcomings with valid and reliable tools and at appropriate timings	[81, 84, 109, 110, 126, 137, 146]
Understand the basis for the need for remediation	[9, 81, 122]
Use of continuous improvement processes	[85, 126, 127, 146]
Provide resources such as remediation toolkits, guidelines, faculty development sessions and workshops	[9, 106, 108, 125, 148]
Having a framework of remediation that clearly defines each stage of remediation for documentation, transparency and communication	[10, 64, 107, 122, 123]
Setting expectations and goals for physician performance	[4, 66, 75, 108, 114, 122, 137, 145]
Collaborative negotiation of remediation plans and goals, reasons for lapses and consequences of failing remediation	[4, 10, 66, 67, 72, 84, 88, 97, 98, 122, 128, 139, 149, 150]
Training mentors and supervisors how to assess, provide meaningful feedback and remediate	[9, 70, 82, 84, 95, 99, 100, 114, 141, 145, 147, 151]
Provide contact with different interdisciplinary experts to allow for a more holistic remediation process	[10, 110]
Protected time	[84, 138, 152]
Increased emphasis on remediation by institutions	[152]
Continuous/frequent monitoring of trainee competencies	[9, 83, 105, 115, 146]
Reframe remediation (not as a punishment)	[80, 122, 146]
Further evaluation of remediation tools’ effectiveness	[101, 103]
**Tutor factors**	
Tight supervision with follow-up	[94, 96, 108, 113, 145, 148]
Faculty as role models	[108, 111, 114]
Address trainee’s personal problems if possible	[84, 122]
Empower the learner to learn at his own pace, self-directed	[9, 70, 133, 146]
**Learner factors**	
Learner must be receptive	[18, 122]
Continuous reflection of the experience	[4, 69, 100, 104, 109, 133, 150]
**Barriers**	**References**
**Institutional factors**	
Lack of standardisation/evidence-based remediation programs/established theory	[9, 10, 62, 64, 73, 76–78, 89, 101, 107, 113, 114, 131, 139, 153]
Time-consuming, resource-expensive	[9, 62, 69, 72, 85, 89, 99, 103, 109, 110, 112, 122, 131, 147, 154]
Suboptimal screening and evaluation methods	[62, 72, 73, 78, 80, 95, 99, 122, 148, 154]
Wrongly identifying residents	[10, 70, 84]
Lack of documentation and clear process to be followed	[63, 73, 77, 95, 123, 136, 139]
Insufficient monitoring of resident performance	[62, 63, 77, 83]
Lack of institutional support	[9, 77, 140, 155]
**Tutor factors**	
Progress and outcomes of trainees can be subjective	[10, 84, 108]
Faculty unwilling to participate in supervising remediation programs	[69, 72, 112, 138]
Reluctance of faculty to fail poorly performing trainees	[62, 95, 107, 115, 122, 136, 154]
Faculty not trained to give feedback	[62, 95, 122, 148, 154]
Emotional drain on faculty given difficulties in remediating trainees	[9, 72, 99, 131]
**Learner factors**	
Learners reluctant to be identified as needing remediation, lack of self-awareness	[65, 66, 69, 73, 88, 91, 92, 98, 100, 114, 122, 127, 137, 150, 155]
High clinical responsibilities of learners	[63, 99, 137]
Some learner deficiencies are not amenable with remediation given incompatible inherent attitudes and learning styles	[61, 90, 122]

### Expert team engagement

In keeping with the SEBA methodology, the expert team reviewed the findings of the review. To determine the validity of our premise, the expert team recommended review of the included articles to determine if the domains identified in this SSR in SEBA were similar to those of the various specialities. This review process revealed 59 articles that included a variety of specialities, 17 articles from surgical specialities, 9 from Emergency Medicine, 7 from medical specialities, and 3 from family medicine.

Comparisons between the domains identified in this SSR in SEBA and those the surgical and medical specialities and the 59 from a variety of speciality revealed similar findings. To triangulate these findings, we found that the domains identified were also consistent with Pirie et al. (2020)‘s review involving trainees from across all specialities in postgraduate medicine, To et al. (2021)‘s scoping review of underperforming surgical trainees and Qi et al. (2021)‘s review of program directors’ perspectives of remediation in graduate medical education.

### Stage 5 of SEBA: Analysis of Evidence-based and Non-data driven Literature

Evidence-based data from bibliographic databases (henceforth evidence-based publications) were separated from grey literature and opinion, perspectives, editorial, letters and non-data based articles drawn from bibliographic databases (henceforth non-data driven) and both groups were thematically analysed separately. The themes/categories from both groups were compared to determine if there were additional themes in the non-data driven group that could influence the narrative.

The key funnelled domains identified from peer-reviewed evidence-based publications were:

- Characterising Remediation
- Reasons for Remediation
- Frameworks for Remediation
- Domains for Remediation
- Enablers and Barriers
- Unique Features of Postgraduate Remediation

The key funnelled domains identified from non-data driven publications were:

- Characterising Remediation
- Reasons for Remediation
- Domains for Remediation
- Enablers and Barriers
- Unique Features of Postgraduate Remediation

There was consensus that themes from non-data driven and the peer-reviewed evidence-based publications were similar and did not bias the analysis untowardly.

### Stage 6: Synthesis of SSR in SEBA

The Synthesis of the discussion of this SSR in SEBA was guided by the STORIES (Structured approach to the Reporting In healthcare education of Evidence Synthesis) statement [156] and Best Evidence Medical Education (BEME) Collaboration guide [157].

## Discussion

In answering its primary research question this SSR in SEBA reveals a number of key findings.

i. Commonalities amongst specialities

This SSR in SEBA reveals commonalities in remediation programs for licenced physicians in training across all specialities. This suggests that whilst contextual factors do impact remediation processes, remediation takes relatively similar forms when carried out under the aegis of similar core competencies and involving learners with similar end goals and abilities. Indeed across the various specialities involved here, remediation is conceived in a similar manner and the indications for remedial support and the approaches adopted are also comparable.

This would suggest that the findings of this SSR in SEBA could be used to guide design, structure, oversight and assessment of remediation programs in any speciality involving licenced physicians in training. However this framework must be infused with local sociocultural, educational, financial, healthcare, legal, ethical and professional factors to be effective.

ii. Individualised approach

In addition any remediation framework must be sufficiently flexible to attend to the “physician in need’s” particular situation, needs, goals, abilities, availabilities, and the gravity of the issue or issues proposed. Thus remediation frameworks must be sufficiently adaptable to contend with the employ of individualised remediation approaches to achieve clearly delineated outcome measures within agreed upon timescales.

iii. Positioning the remediation program

In keeping with an integrated view where remediation processes run concurrently with training programs, concerted efforts must be made to help faculty and ‘physicians in need’ view remediation processes in this role. Being part of the formal program would also ensure faculty are provided with the time, training and effective means of identifying and addressing knowledge gaps, skills shortfalls, attitudinal issues and professionalism lapses. A formal remediation program overseen by curricula designers and administrators will be better able to meet the personalised nature of the remediation process and provide the individualised approach, support, assessment, feedback and oversight necessary to meet agreed upon targets without compromising its structured nature.

iv. Considering the remediation stakeholders

In highlighting the need for a remediation framework that is sufficiently flexible to attend to the individualised needs of the ‘physician in need’, this SSR in SEBA also underlines the roles of the main stakeholders. These are the tutors, the host organization and the physician in need.

- The faculty

It is clear that faculty involved in remediation, require time, training and support to meet their responsibilities and goals [10, 108]. Longitudinal support of faculty must include training in carrying out coordinated assessments, facilitating multisource feedback; and providing timely, appropriate, personalised, longitudinal and specific support across a variety of settings [10, 108]. These processes must be coordinated, guided by a clear set of expectations, roles and responsibilities, timelines and an agreed upon and personalised set of outcomes that consider the risk to patient care; the nature and severity of the competency, attitudinal and performance and or performance deficits; the physician in needs’ position, abilities, experiences, skills, knowledge, attitudes and competencies; the timelines determined; and the practical considerations involved [84, 88, 94, 101]. A code of practice, roles, responsibilities, assessment methods, remediation approaches, outcome measures, oversight of the remediation process and subsequent follow up also help guide faculty in shaping their approach [84, 88, 94, 101].

b. The host organization

These considerations fall upon the host organization to establish within the remediation. Tasked with ensuring patient safety, the host organization must ensure effective assessment processes to establish the issues affecting a physician in need, the risk to patient care, fellow professionals, team working and professional practice and must consider professional standards, institutional codes of practice, regnant sociocultural considerations and administrative policies.

The host organization is also responsible for providing physicians in need with an effective chance to remediate and meet their overall goals. To begin the host organization must help nurture an effective remediation and learning environment [84, 88, 94, 101]. Kalet, Guerrasio [10] noted that remediation’s personalised approach relies upon the cultivation of a safe, collaborative and student-centred, non-judgemental learning environments [10, 18, 104, 108, 146, 158]. Incumbent to this approach is also ensuring a clear set of expectations, roles and responsibilities, timelines and a clear set of outcomes [84, 88, 94, 101].

c. The physician in need

Partially discussed but not given pride of place in the centre of the discussions about remediation is the willingness of physicians in need to acknowledge their gaps, engage in remediation and invest in completing the remediation and re-integration into their training processes. Echoing Price, Wong [4]‘s review of remediation the role of the physician in need’s motivations, perceptions, availability, and ability demands proper considerations in planning the course of the process. However perhaps just as importantly, their willingness, engagement and motivations to remediate must also be considered.

### The multi-theories model of adult learning

Building upon Al-Sheikhly, Östlundh [159]‘s suggestions of using the adult learning theory and Kolb’s experiential cycle to guide assessment, structuring and engagement of the physician in need in the remediation process we adopt Taylor and Hamdy [160]‘s Multi-theories Model of Adult Learning that contains both theories.

To begin, the physician in need of remediation must see and understand the necessity for remediation within their particular situation, be provided advice, evidence and support to participate in this process. Based upon Taylor and Hamdy [160]‘s Multi-theories Model of Adult Learning faculty must help the physician in need move through the following phases of the remediation process

- **dissonance**, where the physician in need identify knowledge, skills, attitudes, and competency gaps.
- **consolidate** feedback and evaluation data
- **refinement**, where the physician in need attempts to understand why certain knowledge, skills, attitudes gap exist
- **organisation**, where they determine the areas of priority, participate in key learning activities, making meaning of new information gathered
- **agree** upon a remediation plan
- **engagement** the physician in need must be prepared to remain engaged throughout the remediation

Here the potential impact upon patient care and the physician in need’s professional responsibilities must be at the heart of the remediation process and underline the rationale for remediation being mandated and formally overseen [80]. However this process must ensure that remediation is not be perceived as a form of punishment that carries the threat of termination, but rather a form of “physician enhancement” [87].

iv. A remediation framework

These considerations form the basis for the proffering of a structured framework for remediation. Our considerations were influenced by Price et al. (2021)‘s update of Hauer et al. (2009)‘s structured approach to remediation and the findings of recent reviews of remediation that have reiterated the importance of structure and contextual considerations. Here the data would suggest that aside from an effective blend of flexibility to meet the personalised nature of remediation and consistency to ensure effective oversight; the nous to select and apply the appropriate tools amidst a wide variety of available tools underscores the need for a guiding framework. Such a framework would help determine the relevant tools to be used and the remediation methods to be employed. Our data also suggests that although other frameworks [107] have been used adaptations to the ACGME framework appears best albeit with effective contextualisation to the education sphere it will be applied to.

**Fig. 3:**
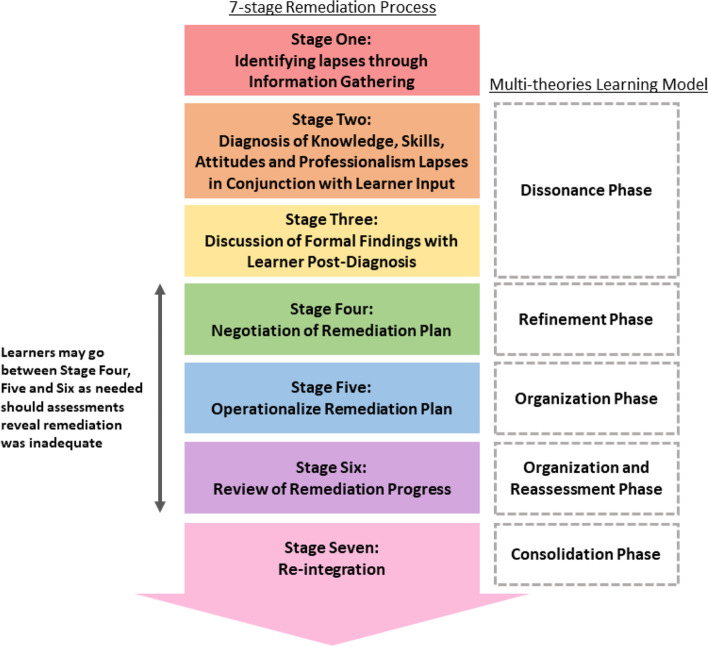
A 7-stage remediation framework built upon Taylor and Hamdy [160]‘s Multi-theories Model of Adult Learning and Hauer, Ciccone [62]‘s remediation framework

We posit that insights from recent reviews of remediation help triangulate our findings [1, 3, 4, 62, 135], and based upon Taylor and Hamdy [160]‘s Multi-theories Model of Adult Learning it is possible to build on Hauer, Ciccone [62]‘s seminal remediation framework to advance a 7-stage remediation framework (Fig. 3).

### A 7-stage remediation process

The first stage begins with identifying knowledge, skills, attitudes or professionalism gaps. A consistent finding across the various competencies is the need for early detection given the potential for competency gaps to impact upon patient care and safety. With data across the competencies suggesting that detection of competency gaps can come from feedback, peer ratings, work-based assessments, supervisor’s evaluations, complaints and interviews, remediation processes are increasingly ‘proactive’ in setting out to detect competency gaps through regular assessments. It is in proactive stance proposed that the role of the faculty supported and guided by the host organization becomes clear.

Here the host organization equips faculty with the appropriate tools and training to identify the physicians in need. In many cases, the tools used appear to be similar across various competencies. It follows that the first stage relies on the presence of an accessible, robust, longitudinal means of providing feedback, curating longitudinal and multisource information on the physician and effective coordination and review of this data. This approach serves to move remediation away from a reactive process to its desired role as an integrated process within the training program that acts upon potential gaps in competency and addressing them in a timely, personalised, appropriate, specific, longitudinal and holistic manner. Conscious of medicine’s hierarchical system and power dynamics within multidisciplinary teams, the feedback or reporting system that this stage is reliant upon should also allow for anonymous feedback from the team to a neutral host organisation. Such a system needs to maintain anonymity of the reporter and yet ensure that the physician is treated fairly [71] whilst the issue is examined. Stage One confirms that issues are indeed present, setting the scene for Stage Two which focuses on diagnosing the problem through a holistic review of the issues [10].

Stage Two acknowledges that gaps in competency levels need to be formally examined particularly when this information will guide the remediation process. Concurrently, to ensure that physicians are fairly treated, and that they are provided with specific evidence and examples of gaps to be addressed as well as the opportunity to question or indeed challenge the feedback provided on them [71, 161], all feedback, results and reports should be reviewed and gaps in competency effectively ‘diagnosed’. Diagnosis includes a review of the physician’s portfolio, input from all stakeholders, and with due consideration of the physician’s psychosocial, academic, personal, research, clinical, professional and practice situation, the physician’s own input and perspective of the issues [81, 84, 108–110, 126, 137]. This holistic perspective is adopted in recognition of data suggesting that competency gaps often involve more than one competency. Diagnosing such multidimensional issues is a time and labour intensive process [85, 147] underscoring the need for the remediation and reporting process to be part of and supported by the education and the student affairs teams [9, 10] within the formal program. Phase one and two underscore the team based approach needed by faculty in diagnosing and supporting the remediation process and highlights the pivotal role of the faculty in supporting it.

Once the diagnosis of the problem is made, Stage Three begins with discussion of the formal findings with the physician to help them understand the issues identified [122].

Integrating Kolb’s Cycle, Taylor and Hamdy [160]‘s theory suggests that it is in Stage Two and Three when knowledge, skills, attitudes and or conduct are challenged and found wanting, the physician enters a Dissonance phase. Here understanding the nature of the problem and how this falls short of expectations and or codes of conduct; acknowledging and reflecting upon the feedback provided; the physician’s own perspective and understanding of the problem and its implications, their motivation to remediate, their particular academic, clinical, research and personal situation, their learning and working environment; and the resources available to them influences engagement with the Dissonance phase and their desire to address this gap and advance their abilities. The Dissonance phase underscores the importance of getting the physician’s ‘buy in’ on the need for remediation, remediation plans, duration, and outcome measures as well as the implications of failing to meet these goals.

The physician then enters stage Four which focuses on the negotiation of a remediation plan [9, 10]. Here they also enter the Refinement phase and contemplates the gaps and issues, reviews practice, remediation options and begins to seek solutions, reflect and discusses their task and the remediation required [153]. It is acknowledged that a learner’s receptiveness to feedback and teaching [18, 122], reflection on their situation, the plan and their remediation experiences [69, 100, 104, 109, 119, 133, 150, 153] is key to remediation success.

Stage Five sees the adoption and operationalization of the personalised remediation plan [9, 10] through a mix of approaches depending on the nature of the issue, context and the physician’s need and goals. This corresponds to the Organization phase as the physician enacts, practices and inculcates the changes needed into their practice and thinking and reflects on and reviews their progress. Here too feedback and holistic support is key to maintaining the physician’s engagement in the process.

Straddling the Organization and Reassessment phases, Stage Six sees the need to review the effects of the remediation as the physician reflects upon their remediation experiences and considers his/her progress. It is only after successful assessments can the physician be allowed to return to their previous role along their training trajectory. However, the timelines should be flexible to allow for changes in the physician’s situation [9].

Part of the Consolidation phase where the physician reflects upon the remediation experience and their learning, Stage Seven emphasises the importance of re-integration without being perceived or judged negatively by others [10, 62, 80]. Here the role of the host organization in changing the program culture is critical. Whilst normalisation of supervision and awareness of supportive structures will prevent burnout and amotivation [69, 113, 144] the need for a change in practice also underscores the importance of the remediation environment [19]. Echoing Cleland, Cilliers [19]‘s review on the remediation environment and in keeping with Price, Wong [4]‘s posit of engagement of the host organization, remediation should be seen as a part of training process and a resource for personal and professional development [9, 125, 146] rather than a failure or punishment [80, 122]. This change in culture and shift in thinking will encourage a conducive and receptive learning and practice environment that appreciates an integrated remediation process and will enhance receptiveness to remediation [18, 122].

Overall the 7-stage remediation framework built upon Taylor and Hamdy [160]‘s Multi-theories Model of Adult Learning and Hauer, Ciccone [62]‘s remediation framework and guided by Cleland, Cilliers [19]‘s review on the remediation environment and Lacasse, Audétat [3], Kalet, Guerrasio [10], Zbieranowski, Takahashi [107], Kurzweil and Galetta [18], Brennan, Price [13], Al-Sheikhly, Östlundh [159] and Price, Wong [4]‘s studies underscores the key finds of this review. These are that remediation is an evolving, personalised and longitudinal process that is influenced by the physician, the faculty, the host organization and the remediation environment. Such particularities undergird the variations reported in prevailing programs and reiterate the need to better understand the process of remediation. The 7-stage remediation framework also affirms the need for holistic support by a team of trained faculty in order a wide variety of reasons for remediation. It is also clear that such support must be guided by a clear framework to contend with the individual nature of the remediation process and overseen by a host organization. A host organization supported program that is effectively integrated into the training program will also help mould the culture and maximise gains for the program.

Finally these recommendations [2, 3, 162–165] are consistent with a number of recent commentaries [9, 10, 15, 18, 79, 85, 101, 166–168] and guidelines on remediation [14, 150, 169].

### Limitations

One of the main limitations of this study was its inability to differentiate residents from more senior doctors such as consultants and senior consultants, which is critical given their different levels of experience, roles, responsibilities and needs. Moreover, whilst this study was intended to analyse the wide range of current literature on postgraduate remediation programs, our review was limited by a lack of reporting of current remediation processes. Furthermore, most of the included papers were largely drawn from North American and European practices potentially limiting the applicability of these findings in other healthcare settings.

Other limitation include our focus on articles that were published in English which may have compounded concerns over the applicability of these findings given the preponderance of articles drawn from North America and Europe. Whilst taking into account the limited resources and availability of the research and experts teams and limiting the review to the specified dates to increase the chances of completing the review, this too could have seen important articles excluded. Conversely, our inclusion of remediation of surgical and medical physicians in training may be an overly inclusive approach. Even though the findings do reflect Pirie et al. (2020)‘s, To et al. (2020)‘s and Qi et al. (2021)‘s limited reviews of residents in training in medicine and surgery [6, 11, 20] and Sparks et al. (2016)‘s review of remediation of anaesthetic fellows [72], Melton et al’s (2018) review of remediation of orthopaedic residents [105], Silverberg et al. (2015)‘s review of remediation of emergency medicine residents [126], Audetat et al. (2015)‘s review of remediation plans amongst family medicine residents [100] and Raman et al. (2018)’ review amongst neurosurgical trainees [170], the relatively small numbers of focused reviews of specific specialities may still be considered an overreach.

## Conclusion

Building upon recent reviews on remediation that have served to triangulate our findings and inspire the synthesis of the 7-stage remediation framework, we believe our theoretically grounded evidence-based 7-stage remediation framework will facilitate the advancement of remediation’s role and value in training programs. However, it is clear it also requires further study to determine its practical value even as Price et al. (2021)‘s recent review on the subject does echo many of our findings. As we look forward to engaging in discussions in this field, we believe future work should also focus on remediation’s role as a support mechanism that acts to prevent breaches and diagnoses and acts upon gaps early on. The impact of remediation on professional identity formation and continuing professional development should also be evaluated.

## Supplementary Information

**Additional file 1.** Appendix.

## Data Availability

All data generated or analysed during this review are included in this published article and its supplementary files.

## Abbreviations

SSR: Systematic Scoping Review
SEBA: Systematic Evidence Based Approach
NUS: National University of Singapore
YLLSoM: Yong Loo Lin School of Medicine
NCCS: National Cancer Centre Singapore
PICOS: Population, Intervention, Comparison, Outcomes, Study Design
RAMSES: Realist And Meta narrative Evidence Syntheses, Evolving Standards
ACGME: Accreditation Council for Graduate Medical Education
MERSQI: Medical Education Research Study Quality Instrument
COREQ: Consolidated Criteria for Reporting Qualitative Studies
CanMEDs: Canadian Medical Education Directives for Specialists
GMC: General Medical Council
ABSITE: American Board of Surgery IN-Training Examination
CMQ: Collège des médecins du Québec
ABPITE: American Board of Pediatrics In-Training Examination
OSCE: Objective Structured Clinical Examinations
EPA: Entrustable Professional Activities
SP: Simulated Patient
STORIES: Structured Approach to the Reporting in Healthcare Education of Evidence Synthesis
BEME: Best Evidence Medical Education
