# Supplementary material for: Post graduate remediation programs in medicine: a scoping review
Source: BMC Med Educ. 2022 Apr 20;22:294. doi: 10.1186/s12909-022-03278-x (PMC9020048; doi:10.1186/s12909-022-03278-x)
Supplement: Supplementary file 1 — Additional file 1. Appendix. [file 12909_2022_3278_MOESM1_ESM.docx]

**Appendix**

Full Search Strategy

("Remedial Teaching"[Mesh] OR Remedial[tiab] OR Remedials[tiab] OR Remediating[tiab] OR Remediation[tiab] OR Remediations[tiab] OR remedy[tiab] OR remediate[tiab]) AND ("Education, Medical"[Mesh] OR "Education, Medical, Graduate"[Mesh] OR "Internship and Residency"[Mesh] OR ((medical [tiab] OR clinical[tiab] or medicine[tiab]) AND (postgraduate[tiab] OR postgraduates [tiab] OR graduate[tiab] OR graduates[tiab] OR resident[tiab] OR residents[tiab] OR residency[tiab] OR residencies[tiab] OR housemanship[tiab] OR housemanships[tiab] OR “house officer”[tiab] OR “house officers”[tiab] OR intern[tiab] OR interns[tiab] OR internship[tiab] OR internships[tiab] OR interning[tiab] education[tiab] OR educations[tiab] OR competence[tiab] OR competency[tiab] OR learner[tiab] OR learners[tiab] OR trainee[tiab] OR trainees[tiab] OR performance[tiab] OR curriculum[tiab] OR syllabus[tiab]))

363750 (1 Feb 2022)

Amended (1 Feb 2022)

("Remedial Teaching"[Mesh] OR Remedial[tiab] OR Remedials[tiab] OR Remediating[tiab] OR Remediation[tiab] OR Remediations[tiab] OR remedy[tiab] OR remediate[tiab]) AND ("Education, Medical"[Mesh] OR "Education, Medical, Graduate"[Mesh] OR "Internship and Residency"[Mesh] OR ((medical [tiab] OR clinical[tiab] OR medicine[tiab]) AND (postgraduate[tiab] OR postgraduates [tiab] OR graduate[tiab] OR graduates[tiab] OR resident[tiab] OR residents[tiab] OR residency[tiab] OR residencies[tiab] OR housemanship[tiab] OR housemanships[tiab] OR “house officer”[tiab] OR “house officers”[tiab] OR intern[tiab] OR interns[tiab] OR internship[tiab] OR internships[tiab] OR interning[tiab] OR education[tiab] OR educations[tiab] OR competence[tiab] OR competency[tiab] OR learner[tiab] OR learners[tiab] OR trainee[tiab] OR trainees[tiab] OR performance[tiab] OR curriculum[tiab] OR syllabus[tiab])))

1929 (1 Feb 2022)

Table 1. Summary of Modalities Utilised in Post-graduate Remediation, guided by ACGME competencies

| **Modality** | **Medical Knowledge** | **Practice-based learning and Improvement** | **Patient Care and Procedural Skills** | **Interpersonal and Communication Skills** | **Professionalism** |
| --- | --- | --- | --- | --- | --- |
| Case discussion | ✔ | ✔ |  | ✔ | ✔ |
| Clinical tutorials, workshops | ✔ | ✔ | ✔ | ✔ | ✔ |
| Lectures | ✔ | ✔ | ✔ | ✔ | ✔ |
| Online courses, assignments, quizzes | ✔ | ✔ |  | ✔ | ✔ |
| Repeat/increased clinical rotations | ✔ | ✔ | ✔ | ✔ | ✔ |
| Readings | ✔ | ✔ | ✔ | ✔ | ✔ |
| Intensified direct supervision |  | ✔ | ✔ | ✔ | ✔ |
| Role-modelling |  | ✔ |  |  | ✔ |
| Direct observation & feedback |  | ✔ | ✔ |  |  |
| Simulations of clinical scenarios |  | ✔ | ✔ | ✔ | ✔ |
| Counselling |  | ✔ |  | ✔ | ✔ |
| Clinical embedding such as ward round presentations |  | ✔ |  | ✔ | ✔ |
| Holistic support |  | ✔ | ✔ | ✔ | ✔ |
| Professional coaching to correct personal behaviour |  |  | ✔ | ✔ | ✔ |

Table 2. Summary of Outcome Measures Utilised in Post-graduate Remediation, guided by ACGME competencies

| **Outcome measure** | **Medical Knowledge** | **Practice based learning** | **Patient Care and Procedural Skills** | **Interpersonal and Communication Skills** | **Professionalism** |
| --- | --- | --- | --- | --- | --- |
| Timing of evaluation | | | | | |
| Post-remediation evaluations | ✔ | ✔ | ✔ | ✔ | ✔ |
| Mid-term evaluations | ✔ | ✔ | ✔ | ✔ | ✔ |
| Quarterly assessment | ✔ | ✔ | ✔ | ✔ | ✔ |
| Daily assessment | ✔ | ✔ | ✔ | ✔ | ✔ |
| Monthly assessment | ✔ | ✔ | ✔ | ✔ | ✔ |
| Nature of Assessments | | | | | |
| Longitudinal | ✔ | ✔ | ✔ | ✔ | ✔ |
| Multisource | ✔ | ✔ | ✔ | ✔ | ✔ |
| Type of Assessments | | | | | |
| Interview, oral exam | ✔ | ✔ |  | ✔ | ✔ |
| MCQs | ✔ | ✔ |  |  | ✔ |
| Objective Structured Clinical Examination | ✔ | ✔ | ✔ | ✔ | ✔ |
| Patient records, documentation | ✔ | ✔ | ✔ | ✔ |  |
| Workplace-based assessment, complaints | ✔ | ✔ | ✔ | ✔ | ✔ |
| Skills practice sessions |  | ✔ |  |  |  |
| Group Discussions |  | ✔ |  | ✔ | ✔ |
| Self-reflection such as reflective essay assignments and reports |  | ✔ | ✔ | ✔ | ✔ |
| Peer-assessment |  | ✔ | ✔ | ✔ | ✔ |
| Trainee satisfaction survey |  | ✔ |  |  |  |
| Survey |  |  |  |  | ✔ |

Table 3. Summary of Included Articles

| **Title** | **Author** | **Year** | **Type of study** | **MERSQI** | **JBI Checklist for Qualitative Papers** | **Methodology** | **Purpose of study** | **Key findings** | **Proposed solutions** |
| --- | --- | --- | --- | --- | --- | --- | --- | --- | --- |
| How resident unprofessional behavior is identified and managed: a program director survey | Adams et al. | 2008 | Quantitative | 13.5 | NA | A web-based survey was emailed to 241 directors of US obstetrics and gynecology residency programs. | To determine how unprofessional behavior by residents is identified/ managed within residency programs, and under what conditions concerns are communicated to potential employers. | 141 program directors (PDs) responded (58%). 84% of PDs indicated that problems with professionalism most commonly come to their attention through personal communication. Methods of addressing the problem included expression of expectation of improvement (95%), psychological counseling (68%), placing resident on probation (59%), and dismissal (30%). The majority of PDs felt remediation was not completely successful. All PDs are willing to communicate professionalism concerns to potential employers, but 42% provide this information only if asked. | Resident unprofessional behavior is a common problem for program directors, and remediation is challenging. PDs are willing to express concerns to potential employers, but a significant percentage indicate concerns only if asked. |
| Pediatric board review course for residents “at risk” | Aeder et al. | 2010 | Quantitative | 14.5 | NA | Residents “at-risk” taking the course (Yes) were compared to residents not taking the course who were not at-risk (No) as well as to a cohort of residents predating the onset of the course with mixed risk (Comparison). Analyses compared board exam scores  and passing rates in the three groups and in the subcategory of residents who scored ≤300 on the in-training exam in each group. | There is a correlation between American Board of Pediatrics In-Training and General Pediatrics Certifying  Examination scores. A course targeted mainly for residents “at-risk” based on in-training scores may improve the  outcome of the certification exam. The objective of the study was to evaluate the impact of a board review course  on pediatric board certification scores, particularly for residents at-risk | Standard scores and pass rates were higher for the residents not at risk, middle for residents of mixed risk and lowest for residents at risk. When analyzing the subset of scores for those with in-training score  of <300, the scores were higher for the residents in the course than those who never took it. Also, residents with  in-training score of <300 achieved higher average point score gains when comparing the certification exam to their  last in-training score. Linear regression analysis showed that not delaying board certification, third year in-training scores and taking the board review course were significantly associated with higher board certification scores. | Pediatric residents at risk for not passing their Pediatric board certification exams benefited from a  board review course. |
| Supporting trainees in difficulty: a new approach for Scotland | Anderson et al. | 2011 | Descriptive | NA | NA | NA | This paper focuses on these three new initiatives through which the four Scottish Deaneries will develop and standardize their processes for managing trainees who fail to progress. | The Framework, the National Advisory Group and the new course provide an infrastructure which underpins the NES approach to supporting clinicians in recognizing and effectively managing trainee doctors in difficulty The added value of the new NES infrastructure is that it provides (1) Access to information; (2) Clarity and consistency; (3) Access to specialist resources. This infrastructure aims to provide a consistent, transparent and sustainable solution to the management of trainees in difficulty. This approach will allow NES to monitor the number of trainees in difficulty, evaluate the management process and initiate changes/improvements as required. This will ensure that adequate support for both trainees and trainers is provided and that employers and Deaneries are appropriately and actively involved. It also allows the four Scottish Deaneries to demonstrate to PMETB that they have in place a clearly defined process for managing any trainee in difficulty. | This infrastructure aims to provide a consistent, transparent and sustainable solution to the management of trainees in difficulty. This approach will allow NES to monitor the number of trainees in difficulty, evaluate the management process and initiate changes/improvements as required. This will ensure that adequate support for both trainees and trainers is provided and that employers and Deaneries are appropriately and actively involved. It also allows the four Scottish Deaneries to demonstrate to PMETB that they have in place a clearly defined process for managing any trainee in difficulty. |
| Why does this learner perform poorly on tests? Using self-regulated learning theory to diagnose the problem and implement solutions. | Andrews, Kelly & DeZee | 2016 | Descriptive | NA | NA | NA | Here we introduce a novel, ready-to-use test-taking assessment and remediation framework based on self regulated learning (SRL) theory; describe implementation at our center; and lay the foundation for a large-scale study of this promising solution to a perennial problem. | At one academic institution, this method was used to categorize learners into struggling test-taker subtypes which correspond to deficiencies in the SRL subprocesses outlined above. A learning plan based on the SRL deficiency was developed for each struggling test-taker subtype. In a small number of internal medicine residents with low in-training examination scores, use of this method yielded improvements in 2013 in-training examination score that doubled the expected improvement based on historical averages. | This method is a novel application of SRL theory to a commonly encountered problem in medical education: the learner who performs poorly on tests. Large-scale, multicentre studies of medical learners at a variety of training levels and program types are needed to determine the effectiveness and generalizability of this intervention. |
| Intern underperformance is detected more  Frequently in emergency medicine rotations | Aram et al. | 2013 | Quantitative | 14.5 | NA | A retrospective review of intern assessment forms from a 2 year period (2009-2010) was conducted at a tertiary referral hospital in Brisbane, Queensland. The frequency of interns assessed as 'requiring substantial assistance' and/or 'requires further development' on mid- or end-of-term assessment forms was determined. Forms were analysed by the clinical rotation, time of year and domain(s) of clinical practice in which underperformance was documented. | To determine the frequency and nature of intern underperformance as documented on in-training assessment forms. | During 2009 and 2010 the overall documented incidence of intern underperformance was 2.4% (95% CI 1.5-3.9%). Clinical rotation in emergency medicine detected significantly more underperformance compared with other rotations (P < 0.01). Interns predominantly had difficulty with 'clinical judgment and decision-making skills', 'time management skills' and 'teamwork and colleagues' (62.5%, 55% and 32.5% of underperforming assessments, respectively). Time of the year did not affect frequency of underperformance. A proportion of 13.4% (95% CI 9.2-19.0%) of interns working at the institution over the study period received at least one assessment in which underperformance was documented. Seventy-six per cent of those interns who had underperformance identified by mid-term assessment successfully completed the term following remediation. | The prevalence of underperformance among interns is low, although higher than previously suggested. Emergency medicine detects relatively more interns in difficulty than other rotations. |
| Remediation plans in family medicine residency | Audetat et al. | 2015 | Quantitative | 11.5 | NA | A multimethods approach in which data were collected from different sources: remediation plans developed  by faculty, program statistics for the corresponding academic years, and students’ academic records and rotation  assessment results. | To assess use of the remediation instrument that has been implemented in training sites at the University  of Montreal in Quebec to support faculty in diagnosing and remediating resident academic difficulties, to examine  whether and how this particular remediation instrument improves the remediation process, and to determine its  effects on the residents’ subsequent rotation assessments. | The framework that was developed for assessing  remediation plans was used to analyze 23 plans produced  by 10 teaching sites for 21 residents. All plans documented  cognitive problems and implemented numerous remediation  measures. Although only 48% of the plans were of good quality,  implementation of a remediation plan was positively associated  with the resident’s success in rotations following the remediation  period. | The use of remediation plans is well embedded  in training sites at the University of Montreal. The residents’  difficulties were mainly cognitive in nature, but this generally  related to deficits in clinical reasoning rather than knowledge  gaps. The reflection and analysis required to produce a  remediation plan helps to correct many academic difficulties  and normalize the academic career of most residents in difficulty.  Further effort is still needed to improve the quality of plans and to  support teachers. |
| Diagnosis and management of clinical reasoning difficulties: Part II. Clinical reasoning difficulties: Management and remediation strategies | Audetat et al. | 2017 | Descriptive | NA | NA | NA | We will describe the difficulties in clinical reasoning and specific remediation strategies in more detail. We will also focus on the specific challenges and issues related to the management of clinical reasoning difficulties. | Difficulties in clinical reasoning should be identified early and appropriate support provided before learners run into major difficulties. The cues available differ by type of supervision, that is, direct observation, case discussions or chart review. Remediation strategies should be selected on the basis of the root causes of the difficulty, the contextual factors and the learners’ needs. Organizational support is crucial for the remediation process given the unpredictability of clinical contexts and locations in which supervisors juggle multiple duties. In most cases, remediation leads to academic success. | Becoming an effective clinical supervisor is a developmental process; clinical teachers need to be supported by ongoing faculty development to strengthen their educational competencies with respect to clinical reasoning characteristics, clinical supervision, as well as educational diagnosis and remediation development processes. That support would make them feel more confident in their supervising tasks and increase their satisfaction in their teaching role. |
| Diagnosis and management of clinical reasoning difficulties: Part I. Clinical reasoning supervision and educational diagnosis | Audétat et al. | 2017 | Descriptive | NA | NA | NA | There are many obstacles to the timely identification of clinical reasoning difficulties in health professions education. This  guide aims to provide readers with a framework for supervising clinical reasoning and identifying the potential difficulties as  they may occur at each step of the reasoning process. | Despite the challenges posed by the clinical setting, it is  possible for supervisors to identify the strengths and weaknesses of their learners’ clinical reasoning. | The understanding  of their own reasoning processes and their  engagement in a process of educational diagnosis of their  learners’ reasoning should help supervisors select appropriate  supervision strategies to facilitate the development of  their learners’ clinical reasoning. |
| Systematic review of the factors and the key indicators that identify doctors at risk of complaints, malpractice claims or impaired performance | Austin et al. | 2021 | Review | NA | NA | Ovid-Medline, Ovid Embase, Scopus and Cochrane Central Register of Controlled Trials were searched from 2011 until March 2020. Reference lists and Google were also hand searched. | To identify the risk factors associated with complaints, malpractice claims and impaired performance in medical practitioners. | Sixty-seven peer-reviewed papers and three grey literature publications from 2011 to March 2020 were reviewed by pairs of independent reviewers. Twenty-three key factors identified, which were categorised as demographic or workplace related. Gender, age, years spent in practice and greater number of patient lists were associated with higher risk of malpractice claim or complaint. Risk factors associated with physician impaired performance included substance abuse and burn-out. | New ways of supporting doctors might be developed, using risk factor data to reduce adverse events and patient harm. |
| Identification of high-risk residents | Bergen et al. | 2000 | Quantitative | 15 | NA | High-risk residents were identified by the former program director. Twenty-four of one hundred fifteen residents over a 10-year period had one to four problematic areas: cognitive, synthetic, family/health, and interpersonal skills. Outcomes included finished (18), voluntary withdrawal (1), and involuntary withdrawal (5). A case-control study matching controls to cases by date of entry into the training program was used. Records were reviewed for demographics, preentry qualifications, American Board of Surgery In-Training Exam (ABSITE) scores, letters of complaint or praise, events of counseling, and monthly ratings. The records of 48 residents were reviewed. Ward evaluations were on eight categories with a 5-point Leikert scale (3-unacceptable to 7-outstanding). The evaluation score assigns points only to low ratings. High scores represent progressively poorer performance. A Wilcoxon signed ranks test was used to compare the cases and controls for continuous variables. The McNemar test was used in comparisons of categorical data with binary outcomes. Exact P values are reported. | Identification of high-risk residents allows remediation and support for administrative action when necessary. This study characterizes differences in documentation of marginally performing residents in a general surgery residency. | Objective data were similar for both groups. Study residents tended to score higher on monthly evaluations at Year 2 and by Year 3 this achieved significance (0.026). Study residents were more likely to have negative faculty letters (0.016) and events of counseling by a faculty member (0.017) and the program director (0.005). | Identification of residents at risk should begin as early as possible during training. A combination of faculty evaluations and evidence of letters of counseling can detect high-risk residents. Programs may use such indicators to support decisions regarding remedial work or administrative action. |
| Remediation of problematic residents—a national survey | Bhatti et al. | 2016 | Quantitative | 9.5 | NA | Cross sectional survey  We conducted a national survey, sending a questionnaire to the program directors of 106 otolaryngology residency programs. We collected information on demographics of the program, identification of problematic residents, and remediation strategies. | Despite careful selection processes, residency programs face the challenge of training residents who fall below minimal performance standards. Poor performance of a resident can endanger both patient safety and the reputation of the residency program. It is important, therefore, for a program to identify such residents and implement strategies for their successful remediation. The purpose of our study was to gather information on evaluation and remediation strategies employed by different otolaryngology programs. | The response rate was 74.5%, with a 2% cumulative incidence of problematic residents in otolaryngology programs during the past 10 years. The most frequently reported deficiencies of problematic residents were unprofessional behavior with colleagues/staff (38%), insufficient medical knowledge (37%), and poor clinical judgment (34%). Personal or professional stress was the most frequently identified underlying problem (70.5%). Remediation efforts included general counseling (78%), frequent feedback sessions (73%), assignment of a mentor (58%), and extra didactics (47%). These remediation efforts failed to produce improvement in 23% of the identified residents, ultimately leading to their dismissal. | Remediation is a complicated process and needs to be tailored to individual needs. This could explain the lack of a uniform approach across programs and the use of multiple remediation efforts for individual residents. Generally, remediation is considered a negative term that may require reporting the remediation period to prospective employers and licensing boards. Avoiding potential adverse consequences associated with formal remediation, general counseling is the most common and probably the first step in remediation followed by frequent feedback sessions and assignment of a mentor. These informal efforts have already been shown to be effective for successful remediation.3 As shown in our survey that personal factors were among the major cause for unsatisfactory performance. Though residents are continuously monitored for their academic achievements, underlying personality or integrity issues are difficult to identify without such informal sessions. Therefore, assignment of mentors who residents trust and would share any issues without hesitance is important. Based on these results, we suggest that assistance should first be provided as informal counseling, while maintaining internal documentation of the process. That documentation may serve as a basis for formal remediation if the problem persists. That next step should include a written remediation plan with a defined timeline, use of resources, and expected outcomes. Actions such as assignment of a mentor, additional didactics, and educational activities may be part of such a plan. Finally, if such efforts do not work, a probation status can be applied, again with clear documentation and defined timeline. Our survey showed that the probation status was officially applied to almost one-third of the residents; the reason might be that the threshold for declaring the probation status was different among PDs. It is important to understand that probation should be reserved only for those with failed remediation or clearly unacceptable behavior such as confirmed substance abuse, falsifying information, or unethical behaviors.9 |
| Common factors among family medicine residents who encounter difficulty | Binczyk et al. | 2018 | Quantitative | 11 | NA | Secondary data analysis was performed on archived resident files from a Canadian family medicine residency program. Residents who commenced an urban family medicine residency program between the years of 2006 and 2014 were included in the study. | The objective of this study was to determine whether there are common factors among the residents who encounter difficulty during training in a large Canadian family medicine residency program. | Five hundred nine family medicine residents were included in data analysis. Residents older than 30 years were 2.33 times (95% CI: 1.27-4.26) more likely to encounter difficulty than residents aged 30 years or younger. Nontransfer residents were 8.85 times (95% CI: 1.17-66.67) more likely to encounter difficulty than transfer residents. The effects of sex, training site, international medical graduate status, and rotation order on the likelihood of encountering difficulty were nonsignificant. | Older and nontransfer residents may be facing unique circumstances and may benefit from additional support from the program. |
| A remedy for resident evaluation and remediation | Boiselle | 2005 | Descriptive | NA | NA | NA | The purpose of this article is to describe how these programs have been developed in the context of the published literature on the subjects of resident evaluation and remediation. | This article describes a  comprehensive method of resident evaluation that supplements the rotation evaluation with several additional methods, including a regularly scheduled faculty “roundtable” discussion of resident performance. It has also described a framework with which to approach resident remediation,  including a resident–residency program director– educational liaison agreement form for dealing with resident performance issues in specific rotations. Throughout,  an emphasis has been placed on the need to involve  the resident in both the evaluation and remediation process. As we gain experience with this new system, and as new research emerges in the field of evaluation and remediation,  our process will continue to evolve, with the goal of continuously improving our ability to detect problems  early and to effectively deal with issues after they are  identified. | As we gain experience with this new system, and as new research emerges in the field of evaluation and remediation, our process will continue to evolve, with the goal of continuously improving our ability to detect problems early and to effectively deal with issues after they are identified. |
| Education or regulation? Exploring our underlying  conceptualisations of remediation for practising physicians | Bourgeois-Law et al. | 2018 | Qualitative | NA | 10 | Semi‐structured interviews were conducted using purposive sampling across the range of Canadian stakeholders involved in the remediation of practising physicians, including regulatory authorities, universities, national certifying bodies and medical organisations. With the use of constructivist grounded theory processes, analysis proceeded apace with data collection in an iterative process, with initial insights guiding subsequent interviews. | This study therefore, explored how various administration‐level stakeholders conceptualise remediation in order to construct a description of the nature and meaning of remediation. | Participants often simultaneously held two different conceptualisations of remediation: (i) remediation as part of an educational continuum involving different degrees of support, and (ii) remediation as a regulatory process removing an individual's educational autonomy. Interviewees moved between these two conceptualisations but did not always appear to be aware of doing so. These conceptualisations each had different implications regarding the degree to which remediation can be incorporated into professional processes of maintaining competence. | Understanding that stakeholders frequently approach the complex issue of remediation with two different perspectives without conscious awareness of doing so may help to explain several challenges in the field, including the issues of what falls under the umbrella of remediation and who should be responsible for offering remediation support. Our findings suggest the need for conceptual clarity around remediation, both to ensure that we illuminate logistical dilemmas in enacting remediation and to address the stigma of ‘de‐professionalisation’ that the provision of even minor educational supports (such as feedback) might invoke by association. |
| Remediation in practice: a polarity to be managed | Bourgeois-Law et al. | 2021 | Descriptive | NA | NA | NA | In this article, we argue that viewing remediation for practicing physicians as a polarity to be managed offers a framework that can further the conversation about how to address some of remediation’s challenges. | NA | We suggest that a successfully managed educationregulation polarity would encourage universities, health organizations, and regulatory authorities to work together to develop remediation programs and might bolster a university’s willingness to deliver such programs. It would also increase remediatee psychological safety, thereby facilitating the insight and motivation necessary for successful remediation and decreasing some of the challenges inherent in the remediator role. Finally, used in conjunction with what is currently known about what works in remediation, it could provide a lens through which programs could analyze their remediation challenges and consider modifications to their processes. |
| Remediating professionalism lapses in medical students and doctors: A systematic review. | Brennan et al. | 2019 | Review | NA | NA | Databases Embase, MEDLINE, Education Resources Information Center and the British Education Index were searched in September 2017 and October 2018. Studies reporting interventions to remediate professionalism lapses in medical students and doctors were included. A standardised data extraction form incorporating a previously described behaviour change technique taxonomy was utilised. A narrative synthesis approach was adopted. Quality was assessed using the Critical Appraisal Skills Programme checklist. | This review sought to identify and assess the effectiveness of interventions to remediate professionalism lapses in medical students and doctors. | A total of 19 studies on remediation interventions reported in 23 articles were identified. Of these, 13 were case studies, five were cohort studies and one was a qualitative study; 37% targeted doctors, 26% medical students, 16% residents and 21% involved mixed populations. Most interventions were multifaceted and addressed professionalism issues concomitantly with clinical skills, but some focused on specific areas (eg sexual boundaries and disruptive behaviours). Most used three or more behaviour change techniques. The included studies were predominantly of low quality as 13 of the 19 were case studies. It was difficult to assess the effectiveness of the interventions as the majority of studies did not carry out any evaluation. | The literature tentatively suggests that remediating lapses in professionalism, as part of a wider programme of remediation, can facilitate participants to graduate from a programme of study, and pass medical licensing and mock oral board examinations. |
| Remedial education: can this doctor be saved? | Cerda et al. | 2000 | Quantitative | 11 | NA | A personalized assessment is designed for each physician accepted into the program … Assessment Report: Ratings, reports and other information of the various assessments are compiled and the Medical Director writes a report on the assessment. The report describes all of the assessments administered and a description of the results. Deficiencies in knowledge or skills, if any, are identified and a personalized continuing education program is prescribed. Personalized programs may include: home-study programs, attendance at CME conferences, self-study assigned readings, preceptorship or visiting mini-fellowship programs, other educational activities. The report is provided to the participating physician and the referring authority … Prescribed Continuing Education: As part of the assessment, physicians are given a detailed description of their deficiencies as well as a detailed remedial education program. Remediation "prescriptions" include: 1. Specific Continuing Medical Education (CME) recommendations. 2. Recommendations for supervision of practice ranging from chart review to direct supervision. 3. Experiential learning consisting of mini-fellowships or residency training. 4. Psychiatric/psychological treatment for an identified mental illness such as depression/anxiety or substance abuse. | However, the important question remains of whether the negative outcome or incident is indicative of an underlying deficiency in knowledge or skill, or represents an isolated and unfortunate incident for a fully competent practitioner. This distinction had to be made by the Board based upon incomplete knowledge of the physician's skills and abilities. The C.A.R.E.S. assessment is individually designed to provide objective information needed in making this important decision. | The UF C.A.R.E.S Program provides a great benefit to physicians and their patients. It provides an atmosphere of professional collaboration and encouragement to address specific educational needs and underscores a commitment to providing continuing medical education, meaningful doctor-to-doctor collaboration, better patient care, and reflects a medical model of diagnosis and treatment of specific problems. | The UF C.A.R.E.S Program provides a great benefit to physicians and their patients. It provides an atmosphere of professional collaboration and encouragement to address specific educational needs and underscores a commitment to providing continuing medical education, meaningful doctor-to-doctor collaboration, better patient care, and reflects a medical model of diagnosis and treatment of specific problems. |
| Training, testing and remediating: the importance of simulation for surgical trainees | Chandra et al. | 2019 | Descriptive | NA | NA | NA | Simulation-based surgical training has become invaluable in training doctors, improving patient safety and offering a safe arena for learning technical skills. With work-hour regulations limiting time spent in hands-on clinical activity (operating, patient care, emergency room evaluations, etc.), the classic apprenticeship model of surgical training is moving towards one based on competency. Simulation allows safe standardization of training and has utility in assessment and remediation. Ingraining deliberate practice and 1:1 feedback into training promote skill acquisition and long-term retention. Objective knowledge/skill assessments help faculty guide and reinforce weak areas among learners. | The evolution of our simulation curriculum has resulted in significantly improved overall performance of our residents across the 5-year continuum. Both anecdotal (successful emergency cricothyrotomies placed in real patients, less misplaced chest tubes, etc.) and legitimate (speed and accuracy of mesh placement for total extraperitoneal inguinal hernia repair, lower infection rates with central line placement, faster skin closure, etc.) experience/data suggest that improving performance with simulated skills does transfer and does improve real clinical outcomes. | Simulation-based training, assessment and remediation allow general surgery residents to learn quickly and effectively in an educational environment that is free of harm to real patients. It is a powerful complement to robust and diverse clinical training. |
| Managing remediation | Cohen, Rhydderch & Cooper | 2014 | Descriptive | NA | NA | NA | We  offer advice on how to navigate and manage the process of  the provision and management of remediation and a case  study using a doctor identified with a performance concern  is used to explore possible pathways for remediation.  Towards the end of the chapter, we also signal some common  problems and pitfalls. | The primary purpose of remediation is to support an individual,  at any level in their career, to move from ineffective  to effective medical practice and/or learning. It is about  building and sustaining change. | To be effective and sustainable  requires a holistic approach to understanding the  nature of performance concerns, their triggers, and impacts.  At the heart of this chapter is the evidence‐based argument  that, as for any complex condition, it is important for those  involved in remediation to simultaneously take account of  health, personal, and social factors. The individual’s wellbeing  should be at the centre of remediation. Tutors, supervisors,  or line managers are central to this process. They  must understand their role (or roles), their boundaries, and  the process and systems they support. They must have a  working knowledge of resources, how to access them, and  the legal frameworks that drive remediation. Well‐designed  remediation can make a difference to not only the individual  but, ultimately, patient care. |
| Assessment and remedial clinical education of surgeons in California | Cosman et al. | 2011 | Retrospective quantitative | 9 | NA | We review 10 years of assessment and  remedial clinical education of general, general/vascular,  and general/colorectal surgeons in the UCSD PACE  Program. | The PACE Program aims to assess competence and  make a plan for remediating any deficiencies that are revealed.  The assessment and remedial education of surgeons  may present special challenges. About 5% of the  physicians participating in the PACE Program have been  general surgeons. | Forty-seven general, general/vascular, and colorectal  surgeons were assessed by the University of California,  San Diego, Physician Assessment and Clinical Education  program in 2000 to 2010. | Assessment and remedial clinical education  of practicing surgeons is feasible and possibly  beneficial. A program of assessment and remedial clinical  education of surgeons designed to meet the needs of one medical board is being used by nongovernmental organizations  as well, and it seems to meet the needs of some  individual surgeons. This type of program may play a role  in the profession’s self-regulation. |
| Dealing with the struggling learner | Crannell & Brasel | 2019 | Descriptive | NA | NA | NA | Every resident will inevitably struggle with some aspect of training. The degree and severity of the struggle, however, will vary and the struggling residents will fall along a continuum. The struggles of a few will not be evident to those in program leadership. One of the most challenging aspects of managing a struggling resident’s remediation is the early identification of one who is faltering. We identify best practices in identification and remediation. | Clearly documenting remediation expectations, re-evaluating the SR progress by other faculty not involved in the remediation process, and/or the use of clinical competency committees, is necessary to determine success or failure. The PD must follow through with consequences. | The SR can be a challenge for the PD and will also be time-consuming, requiring considerable faculty dedication. With early identification and a true understanding of the SR’s deficit, however, specific and targeted remediation with deliberate practice results in success rates in the range of 48.6%-90% dis likely to guide the SR to complete training and to have a successful surgical career. |
| Simple frameworks for daily work: innovative  strategies to coach residents struggling with time  management, organization, and efficiency | DeKosky et al. | 2018 | Qualitative | NA | 8 | Assessments of residents and fellows referred to the EIRC between July 2014 and October 2016 were reviewed for organization and efficiency deficits. Common areas were identified, and an iterative process of learner observations and expert input was used to develop remediation tools. | Organization and efficiency is not a recognized clinical competency, and there is little information on strategies for remediating these deficits. We sought to determine the major themes of organization and efficiency deficits at our institution and develop tools for their remediation. | Over a 2-year period, the EIRC developed remediation plans for 4% of residents (13 of 342 total residents), and for 1 internal medicine subspecialty fellow. Organization and efficiency was the primary or secondary deficit in more than half of those assessed. Most common deficiencies involved admitting a patient efficiently, performing effective pre-rounding, and composing daily progress notes/presentations. Remediation tools that provided deconstruction of tasks to their most granular and reproducible components were effective in improving performance. | Deficits in organization and efficiency can disproportionately affect resident performance and delay milestone achievement. Many residents would benefit from detailed frameworks and assistance with new approaches to basic elements of daily work. |
| “Problem residents”: prevalence, problems and  remediation in the era of core competencies | Dupras et al. | 2012 | Quantitative | 10.5 | NA | The Association of Program Directors in Internal Medicine Survey Committee develops and deploys an annual survey of internal medicine residency program directors to address important issues in graduate medical education. The survey process has been described.5,6 For this study, e-mail notifications with program-specific hyperlinks to a Web-based questionnaire were sent in August 2008 to each Association of Program Directors in Internal Medicine institutional member (program directors) (372), representing 97.1% of 383 US categoric internal medicine residencies.7 Reminders were sent to nonresponders biweekly until the survey closed in November 2008. The survey collected information on characteristics of the program and program directors in addition to the program director’s experiences with residents in difficulty during the 2007-2008 academic year. The entire survey and results are available online.8 Survey responses were appended with data from publicly available sources, including the US Census Bureau,9 American Medical Association Fellowship and Residency Electronic Interactive Database Access System Online,10 and ABIM.11 Data from the public ACGME website included accreditation cycle length, government affiliations, number of approved and filled training positions, and program director appointment date.12 The study was approved by the Mayo Clinic Institutional Review Board. | The purpose of this study was to assess internal medicine program director experiences with residents in difficulty in the era of Accreditation Council for Graduate Medical Education (ACGME) competencies. | Our results suggest that current application materials do not provide the information needed to avoid selecting future residents in difficulty. The majority of residents in difficulty have problems in multiple competencies. A comprehensive and multisource evaluation system is necessary to identify residents in difficulty, because individual residents are unable to see their own deficient behaviors. Remediation remains difficult for all the competencies, but most troubling is the challenge of remediating unprofessional behavior. | Our results suggest that current application materials do not provide the information needed to avoid selecting future residents in difficulty. The majority of residents in difficulty have problems in multiple competencies. A comprehensive and multisource evaluation system is necessary to identify residents in difficulty, because individual residents are unable to see their own deficient behaviors. Remediation remains difficult for all the competencies, but most troubling is the challenge of remediating unprofessional behavior. |
| Perspective: Viewing "strugglers" through a different lens: How a self-regulated learning perspective can help medical educators with assessment and remediation | Durning et al. | 2011 | Descriptive | NA | NA | NA | The authors propose a theory-based framework, Self-Regulated Learning– Microanalytic Assessment and Training (SRL-MAT), that is specifically designed to foster individual medical trainee self-regulatory beliefs and behaviors, and thus provide a distinct method to assist medical trainees who struggle. | Trainees who struggle in medical school or residency might lack the regulatory skills to consistently monitor and adapt their learning efforts to maximize their performance. As a result, evaluating these skills and processes during medical training may not only help medical trainers better understand the processes that inhibit growth in their students but also may help them devise remediation or instructional plans to help these students improve. | SRL microanalytic methodology can be applied to virtually any well-defined task, event, or activity. In medical education, some of the key tasks on which we expect medical students to reach proficiency include history taking, physical examination, and communication. |
| Addressing physicians’ impaired communication skills | Egener | 2008 | Descriptive | NA | NA | The coaching model consists of a discrete set of communication skills that are gradually integrated into professional activities while debriefing that process in a supportive relationship. | This report describes the use of a coaching model developed by the author to remediate inadequate communication skills. | Outcomes are provided for the first 13 physicians coached after the approach was standardized. On a Likert scale (range, 1–7), with 7 expressing “high satisfaction,” all participants rated the consultation in the 5–7 range (mean, 6.3), and all supervisors rated the consultation in the 6–7 range (mean, 6.7). | A coaching model is effective in improving communication skills deemed inadequate by physicians’ patients and colleagues. Future work should evaluate the impact of integrating coaching into health care organizations and on developing new tools to augment coaching. |
| Situating remediation: accommodating  Success and failure in medical education  systems | Ellaway et al. | 2018 | Perspective | NA | NA | This Perspective sets out a conceptual  review of current practices and an  argument for a holistic approach to  remediation in the context of their parent medical education systems. | There has been a widespread shift to competency-based medical education (CBME) in the United States and Canada. Much of the CBME discourse has focused on the successful learner, with relatively little attention paid to what happens in CBME systems when learners stumble or fail. Emerging issues, such as the well-documented problem of “failure to fail” and concerns about litigious learners, have highlighted a need for well-defined and integrated frameworks to support and guide strategic approaches to the remediation of struggling medical learners. | The authors propose parameters for integrating remediation into CBME and describe a model based on five zones of practice along with the rules of engagement associated with each zone. The zones are “normal” curriculum, corrective action, remediation, probation, and exclusion. The authors argue that, by linking and integrating theory and practice in remediation with CBME, a more integrated systems-level response to differing degrees of learner difficulty and failure can be developed. The proposed model demonstrates how educational practice in different zones is based on different rules, roles, responsibilities, and thresholds for moving between zones. A model such as this can help medical educators and medical education leaders take a more integrated approach to learners’ failures as well as their successes by being more explicit about the rules of engagement that apply in different circumstances across the competency continuum. | We hope that this model may provide a framework for further research on developing medical competence, as well as helping to better define the expertise needed to conduct effective remediation, better manage educational resources, and better embody compassion for all our learners. |
| Character failings in the surgeon fallen from grace: a thematic analysis of disciplinary hearings against surgeons | Elledge & Jones | 2020 | Review | NA | NA | We requested previously published transcripts of conduct hearings involving surgeons carried out by the Medical Practitioner Tribunal Service (MPTS) in the UK between 2016 and 2020. A deductive thematic analysis of transcripts  was carried out by the authors using the method  described by Braun and Clarke9–11 and adhering  to the Consolidated Criteria for Reporting Qualitative Research. | We sought to examine the character failings of surgeons who faced fitness to practice enquiries under the Medical Practitioner Tribunal Service in the UK. In particular, we examined the absence of virtue as perceived through the lens of Aristotelian ethics using thematic analysis of tribunal hearing transcripts from 2016 to 2020. | We identified three overarching themes that are explored in depth: ’the god complex’, ’reputation over integrity’ and ’wounded pride’. | We hope to use this as the foundation for a re-examination of the place of phronesis in postgraduate surgical education, which we argue should be perceived as an exercise in character development and reformation rather than the simplistic teaching of skills to standardised outcomes. |
| Resident remediation in family medicine residency programs: a cera survey of program directors | Frazier et al. | 2021 | Quantitative | 8.5 | NA | This study analyzed responses from the Council of Academic Family Medicine Educational Research Alliance (CERA) national survey of family medicine program directors in 2017. Survey questions included topics on faculty remediation training, remediation prevalence, tools for remediation, and barriers to remediation. | The objective of this study was to evaluate the remediation landscape in family medicine residency programs by investigating resident remediation characteristics, tools to improve the process, and remediation challenges. | 267 of 503 program directors completed our survey (53% response rate). Most residency programs (245/264, 93%) had at least one resident undergoing remediation in the last 3 years. A majority (242/265, 91%) of residents undergoing remediation were successful within 12 months. The three most important tools to improve remediation were an accessible remediation toolkit (50%), formal remediation recommendations from national family medicine organizations (20%), and on-site faculty development and training (19%). The top-two challenges to the remediation process were a lack of documented evaluations to trigger remediation and a lack of faculty knowledge and skills with effective remediation strategies. | National family medicine organizations like AFMRD and STFM could develop a task force to develop a standardized remediation toolkit based on the core competencies and milestones. |
| Objective assessment of general surgery residents followed  by remediation | Gas et al. | 2016 | Quantitative | 12 | NA | Postgraduate year (PGY) 2 to 5 general surgery (GS) residents were tested in a 5 station, objective structured clinical examination style event called the Surgical X-Games. Stations were 15 minutes in length and tested both surgical knowledge and technical skills. Stations were scored on a scale of 1 to 5 (1 = Fail, 2 = Mediocre, 3 = Pass, 4 = Good, and 5 = Stellar). Station scores ≤ 2 were considered subpar and required remediation to a score ≥ 4. Five remediation sessions allowed residents the opportunity to practice the stations with staff surgeons. Videos of each skill or test of knowledge with clear instructions on how to perform at a stellar level were offered. Trainees also had the opportunity to checkout take-home task trainers to practice specific skills. Residents requiring remediation were then tested again in-person or sent in self-made videos of their performance. | Surgical training programs often lack objective assessment strategies. Complicated scheduling characteristics frequently make it difficult for surgical residents to undergo formal assessment; actually having the time and opportunity to remediate poor performance is an even greater problem. We developed a novel methodology of assessment for residents and created an efficient remediation system using a combination of simulation, online learning, and self-assessment options. | A total of, 35 residents participated in the Surgical X-Games in the spring of 2015. Among all, 31 (89%) had scores that were deemed subpar on at least 1 station. Overall, 18 (58%) residents attempted remediation. All 18 (100%) achieved a score ≥ 4 on the respective stations during a makeup attempt. Overall X-Games scores and those of PGY2s, 3s, and 4s were higher after remediation (p < 0.05). No PGY5s attempted remediation. | Despite difficulties with training logistics and busy resident schedules, it is feasible to objectively assess most GS trainees and offer opportunities to remediate if performance is poor. Our multifaceted remediation methodology allowed 18 residents to achieve good or stellar performance on each station after deliberate practice. Enticing chief residents to participate in remediation efforts in the spring of their final year of training remains a work in progress. |
| Use of an Objective Structured Clinical Exam (OSCE) for early identification of communication skills deficits in interns | Goldhamer et al. | 2018 | Quantitative | 15 | NA | Ninety-seven interns matriculating to eight residency programs in six specialties at four hospitals participated in a  nine-station communication skills OSCE. Ratings were based on the “Kalamazoo, adapted” communication skills checklist.  Possible association with intern performance evaluations was assessed by repeated-measures logistic regression and ROC  curves were generated. | There is limited information about whether OSCE during GME orientation can identify trainee communication  deficits before these become evident via clinical performance evaluations. | The mean OSCE score was 4.08 ± 0.27 with a range of 3.3–4.6. Baseline OSCE scores were associated with subsequent  communication concerns recorded by faculty, based on 1591 evaluations. A 0.1-unit decrease in the OSCE communication  score was associated with an 18% higher odds of being identified with a communication concern by faculty  evaluation (odds ratio 1.18, 95% CI 1.01–1.36, p.0.034). ROC curves did not demonstrate a “cut-off” score (AUC.0.558).  Non-faculty evaluators were 3–5 times more likely than faculty evaluators to identify communication deficits, based on 1900  evaluations. | Lower OSCE performance was associated with faculty communication concerns on performance evaluations;  however, a “cut-off” score was not demonstrated that could identify trainees for potential early intervention. Multi-source  evaluation also identified trainees with communication skills deficits. |
| Influence of remedial professional development programs for poorly performing physicians | Goulet, Gagnon & Gingras | 2007 | Quantitative | 13.5 | NA | For each physician, 30 to 50 patient records were selected randomly for review. Ratings were assigned for the quality of record keeping and for 3 elements pertaining to the quality of care: the clinical investigation plan, diagnostic accuracy, and patient treatment and follow-up. The impact of the program was measured by comparing the proportion of physicians with satisfactory ratings assigned by peer review before and after the remedial professional development program. | The aim and the originality of the present retrospective study was to verify whether poorly performing physicians who were referred to the Practice Enhancement Division (PED) of the Collège des médecins du Québec (CMQ) showed any significant improvement in their clinical performance as evaluated by a formal assessment tool, the peer review process, after completing an individualized RPDP. | Overall, this study suggests that completion of the individualized RPDP organized by the PED has a positive effect on the quality of practice of targeted poorly performing physicians. Following completion of an RPDP, a substantial number of physicians who experienced problems appear to incorporate their new knowledge and skills into their daily practice. Key findings include the following (1) Professional development programs must target a physician’s specific deficiencies by using an individualized approach. (2) Remediation programs should be completed on a 1-on-1 basis, allowing tutors to proceed at the physician’s pace and to adapt to his or her learning capacity. (3) When poorly performing physicians take part in a remedial professional development program, are not ill, and have learning capacity, there can be improvement. | Our findings show that there is room for improvement. Even after successfully completing an RPDP, 29 of the physicians assessed (43%) still kept inadequate records. The medical profession must be convinced of the importance of proper record keeping. |
| An innovative approach to remedial continuing medical education, 1992–2002 | Goulet, Jacques & Gagnon | 2005 | Quantitative | 13 | NA | The CMQ (the Quebec medical licensing authority) developed a process to identify  physicians who had shortcomings in their clinical performance, determine their  educational needs, propose, in  collaboration with the four medical  schools in the province, personalized  retraining programs (clinical training  programs, tutorials, focused readings,  workshops, and refresher courses), and subsequently evaluate the impact of these retraining programs. | The authors describe the process of  remedial retraining programs organized  and planned for Quebec physicians by  the College des medecins du Quebec  (CMQ) and report the outcomes of these  efforts from April 1992 to March 2002. | During the ten-year period reported, 305  physicians (216 family physicians and 89 specialists) were referred to the Practice  Enhancement Division of the CMQ for  personalized remedial retraining. The  vast majority of these physicians were  men (81%). The following difficulties  were identified: therapeutic knowledge  (37%), diagnostic knowledge (32%),  record-keeping (14%), technical skills  (10%), clinical judgment (5%), and  communication skills (2%). A total of  329 personalized retraining programs  were completed: 273 clinical training  programs, 41 tutorials, and 15 focused  readings. A reevaluation of all these  physicians showed that 70% of the  retraining programs had succeeded,  15% were partially successful and only  13% had failed. The remaining 2%  involved missing data or withdrawal of  physicians. | The authors conclude that the collaborative CME process described has important and effective original features. |
| Physician, heal thyself | Graham | 2021 | Descriptive | NA | NA | NA | NA | NA | I would highly encourage my younger self to develop a toolkit for health that emphasizes sleep hygiene, physical exercise, a healthy diet and, most critically, a mental health practice that includes meditation. Books like Why We Sleep, by Andrew Walker,[2](https://www.cmaj.ca/content/193/41/E1601#ref-2) outline the incredible physical and mental health consequences of poor sleep, and strategies to improve it. In high school or university, my peers would probably have voted me least likely to ever practise meditation or yoga, but there are important lessons in a meditation practice that give insight into managing stress and anxiety, and it is not hyperbole to say they have radically improved my life. Mobile applications such as Waking Up or Headspace make learning meditation easy, and managing my thoughts and emotions is now a fundamental pillar of my self-care. |
| Long-term outcomes of a simulation-based  remediation for residents and faculty with  unprofessional behavior | Guerrasio & Aagaard | 2018 | Qualitative | NA | 9 | Residents and faculty were referred for unprofessional behaviors, including aggressive, condescending, and argumentative communication styles as well as an inability to read social cues. We had standardized patients recreate the scenarios that triggered the unprofessional behavior. After each scenario, participants reviewed a videotape of their performance, participated in guided self-reflection and feedback, and then iteratively practiced skills. In 2017, about 2 to 4 years after the intervention, we conducted structured phenomenological qualitative interviews until thematic saturation was reached. Transcripts were analyzed inductively for themes by 2 reviewers (J.G. and research assistant). | There are few studies describing remediation for unprofessional behavior in residents and faculty and none that assess the long-term impact of remediation. We implemented a simulation-based personalized remediation program for unprofessional behavior in residents and faculty and collected assessments from participants and referring supervisors. | Requests for interviews were sent to 16 residents, 8 faculty members, and 24 supervisors, including program directors. Nine remediation participants (38%) and 19 referring supervisors (79%) were interviewed. Sixteen supervisors reported no recurrence of unprofessional behavior in participants 2 to 4 years after the intervention, and participants identified behavioral strategies to reduce unprofessional behavior. Participants and respective supervisors reported similar themes of behavior changes that resulted in improved professional interaction with others. | A simulation-based personalized remediation program for unprofessional behavior, where faculty and residents practice behaviors with guided feedback, can lead to sustained positive behavior change in participants. |
| Methods and outcomes for the remediation of clinical reasoning | Guerrasio & Aagaard | 2014 | Quantitative | 10 | NA | Between July 2006 to June 2012, of 151 learners referred to the remediation program, 53 were identified as having clinical reasoning deficits. The remaining 98 learners entered a different arm of the remediation program.12 One of the remediation specialists kept confidential records of the learners enrolled, their level of training, their identified deficits, and academic outcomes. All remediation was provided by a remediation specialist through one-on-one instruction and case review, which included both face-to-face time and e-mail correspondence related to implementing the remediation plan. One-on-one time was recorded in hours. Faculty were asked to record time reading and replying to emails from individual learners that directly related to providing feedback and asking reflective questions that corresponded with the various steps in the remediation plan. Faculty members were asked to record the time in 10 min increments on a spreadsheet. The remediation team implemented a standardized remediation plan based on the current understanding of the clinical reasoning process and deliberate practice. While the steps were standardized for all learners, learners progressed through the steps at their own rate. The support of the remediation specialist was removed when all ten steps were completed. Reassessment was performed post remediation by an independent faculty member or team, as part of a routine clinical assessment based on the level of the learner; assessors were unaware that the learner was being remediated. Reassessment methods included observed structured clinical examinations with standardized patient encounters and script concordance testing,33 repeating part or all of a rotation, mini-clinical evaluation examinations, chart stimulated recall, and attending and direct team rotation evaluations. The results of the reassessments were shared with the rotation or program director, who determined whether remediation was a success or failure. If the learner’s skills met minimum competence for his/her level of training at this assessment, the learner was permitted to continue his training. Otherwise, remediation continued and the learner was subsequently reassessed. If comorbid deficits had not resolved through remediation of clinical reasoning, the learner entered an arm of the remediation program focused on the remaining deficit(s). | To assess the effectiveness of a standardized clinical reasoning remediation plan for medical learners at various stages of training. | The ten-step remediation plan for clinical reasoning was time consuming for faculty but successful for the participants, with the majority (96 %) passing the reassessment and 91 % deemed competent for graduation and continued practice. The remediation plan was intentionally created using our understanding of the clinical reasoning process and prior work on the development of expertise.14–29,34,35 The steps provide a concrete way of teaching discrete skills needed to problem solve in medicine. | Next steps are to study this method at different institutions for continuous improvement of better outcomes and efficiency, and to study patient care outcomes |
| Determining need for remediation  through postrotation evaluations | Guerrasio et al. | 2012 | Quantitative | 12.5 | NA | This retrospective case-control study examines the ability of post rotation evaluation characteristics to predict the need for remediation. We compared the evaluations of 17 residents who were placed on academic warning or probation, from 2000 to 2007, with those for a group of peers matched on sex, postgraduate year (PGY), and entering class. | Post rotation evaluations are frequently used by residency program directors for early detection of residents with academic difficulties; however, the accuracy of these evaluations in assessing resident performance has been questioned. | The presence of an outlier evaluation, the number of words written in the comments section, and the percentage of evaluations with negative or ambiguous comments were all associated with the need for remediation (P = .01, P = .001, P = .002, P = < .001, respectively). In contrast, United States Medical Licensing Examination step 1 and step 2 scores, total number of evaluations received, and percentage of positive comments on the evaluations were not associated with the need for remediation (P = .06, P = .87, P = .55, respectively). | Our study demonstrates that post-rotation evaluation characteristics can be used to identify residents as risk. However, larger prospective studies, encompassing multiple institutions, are needed to validate various evaluation methods in measuring resident performance and to accurately predict the need for remediation. |
| The evolution of resident remedial teaching at one institution | Guerrasio et al. | 2019 | Descriptive | NA | NA | NA | In this Article, the authors aim to describe the development and outcomes of the program and to explore possible reasons for the improved outcomes. | As we have updated and accrued more data since our prior publications, our overall remediation success rates have improved steadily. The CRP currently faces several challenges in the form of limited resources. The program is continuously seeking faculty from a wider diversity of specialties to join the remediation team. The program has seen turnover among highly used faculty, often due to emotional fatigue resulting from their high level of investment in individual learners. The high financial, personnel, and time costs of using the simulation center, including building individualized case scenarios and training standardized patients, have restricted its use. Lastly, advertising the CRP has been challenging because of the large number of training programs and the time needed to reach out to new program directors. | A standardized framework for remediation can have sustained positive outcomes. We are also hoping that, in 5 to 10 years, ongoing data analysis of this program will show significantly improved outcomes for residents who are placed on probation and engage in remediation through a centralized process. |
| Remediation of low ABSITE scores | Harthun et al. | 2005 | Quantitative | 11 | NA | One faculty member was designated to meet with each resident with ABSITE scores below the standard described above. In April, individual study plans were designed with each resident, which emphasized practice questions and strategic planning for study, and the ABSITE test structure and results were reviewed. Two subsequent meetings were held between November and December to monitor progress. Emphasis was placed on minimizing the stigma of poor scores and on maximizing the efficiency of study time. If the resident scored above standard that year, further support was not required. | In 2000, a program was initiated to improve American Board of Surgery In-Training Exam (ABSITE) scores below the 40th percentile (PGY-1 and -2) and the 30th percentile (PGY-3 and -5) for categorical residents. The goal of this program is to maintain scores above this standard. | From 2000 to 2004, 12 (9.5%) ABSITE scores were below-standard, which resulted in 8 (20.5%) residents receiving program support 9 times. All but 1 program encounter resulted in above-standard scores the following year (improvement range, 16 to 65 percentile points; average, 34 points). Two residents had recurrent below-standard scores in subsequent years despite above-standard scores immediately after the program. One resident did not participate in the program, despite it being designated as mandatory. During the same interval, the ABSITE scores of residents not involved in the program decreased by an average of 3.7 percentile points per examination (improved scores 31 times; 39.2%, range 1 to 46, average 13.5, worse scores 45 times; 57%, range 1 to 65, average 15.2, and no change 3 times, 3.8%). | : An individualized program that minimizes the stigma of poor test results, the time commitment required by the residents, and maximizes the benefits of a question-based study system and the knowledge of approach to the ABSITE resulted in significant improvements in scores the next year. However, maintenance of these results needs continued evaluation because 3 residents had recurrent poor scores in subsequent years. |
| Remediation of the deficiencies of physicians  across the continuum from medical school  to practice: a thematic review of the  literature | Hauer et al. | 2009 | Review | NA | NA | The literature search focused on studies of remediation that took place in undergraduate medical education (UME), GME, and continuing medical education (CME) of post licensure physicians. We searched the MEDLINE database through April 2008 for citations by using terms related to remediation (remediation or remedial teaching), level of practitioner (medical students, clinical clerks, internship and residency, and physicians), and other related terms (clinical competence; program evaluation or program development; educational measurement, curriculum, or model; and mentors). We extended the search through October 2008 to identify any newly published studies. In addition, we manually searched the bibliographies of relevant retrieved articles and identified articles from our personal knowledge of the field. We included English-language studies and excluded opinion articles, review articles, descriptions of curricula without a remediation group, and surveys on remediation. We developed a standardized data extraction form based on the Best Evidence Medical Education Collaboration protocol.28 We extracted the following information from each article: level and number of learners/physicians, study location, description of assessment, skill area, criteria for remediation (standard setting), remediation activities, retesting, and outcomes of remediation. We assessed the level of behavioral impact by using the four-level Kirkpatrick hierarchy to assess the strength of the intervention. | Despite widespread endorsement of competency-based assessment of medical trainees and practicing physicians, methods for identifying those who are not competent and strategies for remediation of their deficits are not standardized. This literature review describes the published studies of deficit remediation at the undergraduate, graduate, and continuing medical education levels | Of 207 citations identified, 170 (63 UME, 43 GME, and 64 CME) were selected for further review on the basis of the title, abstract, and, when relevant, the full article. Selected articles contained all three components of remediation as listed in Methods (i.e., identification of performance deficit, remediation intervention, and reassessment of performance after intervention). Thirteen studies met eligibility criteria; the results are described here and in Appendix 1. Articles that were initially reviewed but excluded were of several types: descriptions of performance-problem identification only or of problem identification and remediation without reassessment of performance, surveys of program directors or other educators about performance problems or remediation, and opinion pieces. | See figure 1 |
| Re-demonstration without remediation--a missed opportunity? A national survey of internal medicine clerkship directors. | Hawthorne et al. | 2014 | Quantitative | 8.5 | NA | A national cross-sectional survey of Clerkship Directors in Internal Medicine (CDIM) institutional members was conducted in April 2011. The survey included sections on remediation, grading practices, and demographics. The authors analyzed responses using descriptive and comparative statistics. | To determine which assessment components are used to assign students a passing grade for the clerkship and what remediation measures are required when students do not pass a component. | Response rate was 73% (86/113). Medicine clerkships required students to pass the following components: clinical evaluations 83 (97%), NBME subject exam 76 (88%), written assignments 40 (46%), OSCE 35 (41%), in-house written exam 23 (27%), and mini-CEX 19 (22%). When students failed a component of the clerkship for the first time, 55 schools (64%) simply allowed students to make up the component, while only 16 (18%) allowed a simple make-up for a second failure. Additional ward time was required by 24 schools (28%) for a first-time failure of one component of the clerkship and by 49 (57%) for a second failure. The presence or absence of true remedial measures in a school was not associated with clerkship director academic rank, grading scheme, or percent of students who failed the clerkship in the previous year. | This study raises the question of whether true remediation is being undertaken before students are asked to re-demonstrate competence in a failed area of the clerkship to be ready for the sub-internship level. |
| Patterns of disrespectful physician behavior at an academic medical center: implications for training, prevention, and remediation | Hopkins et al. | 2018 | Quantitative | 12 | NA | The authors conducted a retrospective analysis of reports of disrespectful physician behavior at Stanford Hospital and Clinics from March 2011 through February 2015. Events were stratified by role, gender, specialty, and location in the hospital or clinics where the event occurred. Event rate ratios were estimated using a multivariable negative binomial regression model. Correlation of rates of faculty and trainees in the same specialty was assessed. | The purpose of this study was to describe these patterns of disrespectful behavior events at an academic medical center. Details of these patterns can be used to create more focused education and training for specific physician groups as well as individualized remediation interventions. | One hundred ninety-nine events concerned faculty; 160 concerned trainees. Events were concentrated among a small number of physicians in both groups. The rates of faculty and trainee events within the same specialty were highly correlated (Spearman’s rho: 0.90; P < .001). Male physicians had an adjusted event rate 1.86 (95% CI = 1.33–2.60; P < .001) times that of females. Procedural physicians were 3.67 times (95% CI = 2.63–5.13; P < .001) more likely to have a disrespectful behavior event than nonprocedural physicians when adjusting for other covariates. Most common location for faculty was the operating rooms (69 events, 34%); for trainees, the medical/surgical units (43 events,  27%). | The patterns of disrespectful behavior by gender, specialty, and location within the hospital can guide the design of more focused education and training for particular groups of physicians and individualized remediation interventions. A small proportion of faculty and trainees accounts for most of the recurring behavior. The low number of physicians displaying the more serious, repeated disrespectful behavior requires further study at other institutions to determine how common this pattern is. Future research is needed to address which interventions will be most successful at decreasing triggers and patterns of disrespectful behavior, and to provide further insight into the design of effective education and training. |
| Principles of remediation for the struggling neurosurgery resident | Jensen et al. | 2020 | Descriptive | NA | NA | NA | The focus of this paper is to supplement these guidelines with emphasis on the individual who is not responsive to the standard approach. The outlined remediation curriculum is designed for use by those individuals at a program involved in training neurosurgical residents, but the principles could also be applied to other types of trainees, including medical students or a new advance practice provider that has been added to a neurosurgical practice. | The specific areas that are assessed for deficits include medical knowledge, clinical reasoning and judgment, clinical skills, time management and organization, interpersonal skills, communication, and professionalism. Specific learning goals and objectives as well as teaching and learning methods pertain to the unique areas of deficit, and all of these must be considered with the goal of developing a resident-specific remediation plan. A plan for assessment of the remediation process is described including an evaluation of what constitutes individual resident remediation success. Finally, a discussion of the prior resident remediation studies across many disciplines is made. | A plan of remediation is necessary when resident physicians miss appropriate milestone competencies. Deficits in medical knowledge, clinical reasoning and judgment, clinical skills, time management and organization, interpersonal skills, communication, and professionalism can be assessed to define a remediation plan formulated to close the specific area of deficiency with learning goals, objectives, and teaching methods, as well as a plan for assessment. Resident remediation for issues other than professionalism that use a well-defined protocol can be quite successful and should be instituted early in the career of a resident identified as lacking in any of the neurosurgical milestones. |
| Remediation of learners who perform poorly on an OSCE | Kalet et al. | 2013 | Descriptive | NA | NA | NA | Data from well-designed OSCEs help educators identify trainees with gaps in their core clinical skills. Yet there is little consensus on effective remediation strategies for individuals who perform poorly. | By definition, learner remediation must have a reasonable chance of leading to an improvement in clinical competence. | Table 3.1 breaks the remediation process down into manageable steps. Effective remediation first of all requires good data. Also crucial, to engage meaningfully in and gain life-long benefit from remediation, learners must have or develop the capacity to accurately self-assess and self-regulate learning. |
| Twelve tips for developing and maintaining a remediation program in medical education | Kalet, Guerrasio & Chou | 2016 | Review | NA | NA | The following 12 tips derive from a decade of remediation experience at each of the authors’ three institutions. It is informed by the input of a group of 34 interdisciplinary North American experts assembled to contribute two books on the subject. | We intend this summary to guide program leaders to build better remediation systems and emphasize that developing such systems is an important step toward enabling the transition from time-based to competency-based medical education. | In this article, we offer 12 tips for best practices at the institutional level for effective remediation in medical education. We base these tips on our own experience conducting  remediation, the educational literature, conversations with medical educators globally through presentations and consultations, and completion of two books on the subject. We list five tips that develop the vision and structure for a program, five tips concerning faculty roles and development, and then two tips on accountability and outcomes. | Taking a systematic and programmatic approach to this challenge is critical to being able to make sound, well-informed, justifiable, and fair decisions about competence that honor our contract with society. A programmatic and rigorous approach is likely to yield rich evidence to inform the movement away from time-based and toward an individualized competency-based medical education system for all trainees. |
| Does academic intervention impact abs qualifying examination results? | Karen R. Borman, MD | 2006 | Quantitative | 12.5 | NA | A mandatory intervention program was begun in April 2001 for residents with ABSITE Total Test (TT) percentiles 31. Program elements included: 1) individual faculty mentoring and personal learning plan 2) QE videotape review  sessions 3) Surgical Education and Self-Assessment Program  (SESAP) 4) monthly rotation evaluations, and 5) quarterly status  feedback. A free medical evaluation was offered. Mock orals  participation, educational psychologist consultation, and voluntary follow up mentoring were added later. Study data were  reviewed for 2003-2005 Chief Residents including ABSITE  scores, QE results, conference attendance, rotation Overall Performance ratings, and resident surgeon case volumes. Results  were compared for the academic intervention (AI) and no intervention  (NI) groups. | To assess the impact of a focused academic support program on American Board of Surgery In-Training Examination (ABSITE) scores and Qualifying Examination (QE) | Fifteen residents graduated during the study period.  Eight residents completed nine interventions; seven returned to TT percentiles 30 (7/8, 88%). First post-intervention ABSITE gains were large compared to NI and national peer groups. Standard  Score (SS)TT gains were maintained until residency completion by four AI residents. Median AI PGY-5 TT percentile was 32 and three scores were25. Six AI residents (6/8, 75%) and all NI  residents (7/7, 100%) passed theQEon their first attempts. AI and NI groups were similar for conference attendance, rotation evaluations, and operative log totals. | A focused academic support intervention  for residents with marginal ABSITE TT percentiles can produce immediate substantial gains. Gains are variably maintained through remaining residency years. PGY-5 TT percentiles 25, seen with three AI residents (3/8, 38%), are associated with a 40% first QE failure rate. Therefore, our 75% QE first-time pass rate for AI residents argues for intervention success |
| A novel concept in residency education: case-based remediation | Katz et al. | 2013 | Descriptive | NA | NA | A case was selected to represent a commonly encountered and serious problem. To ensure protection of the resident, details of the case that might potentially identify the resident were changed before presentation to the committee. The anonymous, altered case was presented to a multidisciplinary panel of current or former program directors that analyzed the process, approach, and assessment. The panel generated an expert consensus opinion based on the four steps outlined by Hauer et al. Institutional Review Board approval was waived. | 1) To describe a complicated resident remediation case and employ consensus panel evaluation of the process. 2) To discuss the available assessment tools (including neuropsychologic/medical testing), due process, documentation, reassessment, and relevant barriers to implementation for this and other resident remediations. | Details of a remediation case were altered to protect resident confidentiality, and then presented to a multidisciplinary group of program directors. The case details, action plan, and course were submitted and the remediation process, action plan, and course are assessed based on a standardized remediation approach. The resident entered remediation for poor organizational skills and an inability to make or follow through with patient care plans. Opportunities for improvement in the applied remediation process are identified and discussed. Legal concerns and utility of neuropsychological assessment of residents are reviewed. | Remediation requires a complicated and detailed effort. This case demonstrates issues that program directors may face when working with residents and provides suggestions for use of specific remediation tools. |
| Guiding principles for resident remediation:  recommendations of the CORD remediation  task force | Katz et al. | 2010 | Descriptive | NA | NA | Faculty members of CORD volunteered to participate in periodic meetings, organized discussions and literature reviews to develop overall guidelines for resident remediation and in a collaborative authorship of this article identifying best practices for remediation. | Remediation of residents is a common problem and requires organized, goal-directed efforts to solve. The Council of Emergency Medicine Residency Directors (CORD) has created a task force to identify best practices for remediation and to develop guidelines for resident remediation. | The task force recommends that residency programs: 1. Make efforts to understand the challenges of remediation, and recognize that the goal is successful correction of deficits, but that some deficits are not remediable. 2. Make efforts aimed at early identification of residents requiring remediation. 3. Create objective, achievable goals for remediation and maintain strict adherence to the terms of those plans, including planning for resolution when setting goals for remediation. 4. Involve the institution's Graduate Medical Education Committee (GMEC) early in remediation to assist with planning, obtaining resources, and documentation. 5. Involve appropriate faculty and educate those faculty into the role and terms of the specific remediation plan. 6. Ensure appropriate documentation of all stages of remediation. | Resident remediation is frequently necessary and specific steps may be taken to justify, document, facilitate, and objectify the remediation process. Best practices for each step are identified and reported by the task force. |
| Approach to handling a problem resident | Kaushik et al. | 2019 | Descriptive | NA | NA | NA | The present review discusses a broad approach to recognizing a problem resident and provides suggestions on remedial measures. | Knowledge, skill deficits, attitude problems and unprofessional behaviours are essential in tackling the problem resident. | Resident doctors face a variety of professional and personal problems, including deficits in knowledge, skills or problems with attitude. Faculty members and institutional heads must be sensitized to handle these problems. Remedial measures need to be individualized and must be framed with the involvement of affected resident. One-to-one faculty mentorship, peer support, psychological consultation, rotating the resident out of difficult workplace, stringent monitoring of their behavior, and providing effective feedback are some of the remedial measures to handle a problem resident. Providing enjoyable learning experiences should be the goal of every residency program. |
| The Kolb learning cycle in American Board of Surgery in-training exam remediation: the accelerated clinical education in surgery course | Kosir et al. | 2008 | Quantitative | 12 | NA | For concrete experience, Surgical Education and Self-Assessment Program (SESAP-13) was completed according to the syllabus. For reflective consideration, further reading was done on SESAP 13 topics and corresponding ABSITE Keywords. For the abstract hypotheses step; these keywords and topics were reviewed with the mentor. Active testing involved a required weekly on-line quiz based on the syllabus. | This is a report of a remedial course for surgical residents that integrated the Kolb learning cycle. The purpose is to determine whether surgical residents placed in the mandatory remedial program increased their score on the revised ABSITE as compared to those not in the remedial program, and whether surgical residents in the remedial program raised their score above the 30th percentile, considered an important national standard compared with those not in the remedial program. | Correct scores on the ABSITE increased for 78.6% of residents in the ACES course, with 28.6% scoring 30th percentile or greater. Senior percent correct scores increased by 7.3% and junior percentile scores by 12.5%. | It also merits consideration of whether eligibility to take the qualifying exam in surgery should begin in the last year of residency in order to motivate effort on performance, something that “fits” adult learning theory. |
| A qualitative study of medical educators’ perspectives on remediation: Adopting a holistic approach to struggling residents | Krzyzaniak et al. | 2017 | Qualitative | NA | 8 | We used a constructivist qualitative design with conventional content analysis, which aims to describe an experience through the coding and interpretation of data to identify relevant themes (Hsieh & Shannon 2005). Our study team consisted of five emergency medicine physicians and one internist. All had training in focus group moderation as well as experience with qualitative data analysis. We anticipated achieving saturation after completion of a minimum of four focus groups with a total of at least 12 participants (Morgan 1997; Guest et al. 2006). Funding was provided by an internal education grant from the Department of Emergency Medicine, University of Colorado School of Medicine. This study took place within the University of Colorado School of Medicine system and was approved by the Colorado Multi-Institutional Review Board. | This study aimed to explore and expand our understanding of GME remediation from perspectives shared among a diverse group of medical educators. We explored current practices in order to gain a richer understanding of educators’ attitudes toward remediation and methods perceived to be successful for identifying struggling residents, identification of common domains where residents struggle, description of personal factors that contribute to a resident’s difficulty and exploration of interventions that constitute successful remediation. Our goal was to develop a novel holistic framework for GME remediation that may be used to inform the design and implementation of remediation efforts. | Nineteen physicians across multiple specialties and institutions participated in seven focus groups. Thirteen categories emerged around remediation. Some themes addressed practical components of remediation, while others reflected barriers to the process and the impact of remediation on the resident and program. The themes were used to inform development of a novel holistic framework for remediation. The approach to remediation requires comprehensive identification of individual factors impacting performance. The intervention should not only include a tailored learning plan but also address confounders that impact likelihood of remediation success. Our holistic framework intends to guide educators creating remediation plans to ensure all domains are addressed. | Remediation remains a challenging process, but this holistic framework may provide guidance for educators taking on a remediation effort. Individual programs should reflect on their current practices to examine whether their approach encompasses these critical points and take action to remedy gaps in their process. |
| The struggling trainee: principles of effective remediation | Kurzweil & Galetta | 2018 | Descriptive | NA | NA | NA | Remediation is challenging for all parties involved, and there is a paucity of literature to help guide the most effective process. In this review, we outline key principles of effective remediation of a struggling trainee in the modern era of medical education. | The best approach to a struggling trainee in need of remediation is a systematic one. At the onset of training, it is important for residents to understand the expectations and goals of the program. These objectives can be introduced in an orientation to the program, and should be reinforced regularly through semi-annual evaluations, resident forums, and other appropriate venues. The methods by which goals are assessed like ACGME milestones and other internal tools should be reviewed regularly as well. Early identification of struggling trainees and early intervention is crucial. Meaningful feedback should be provided to effectively improve a resident’s deficiency. A remediation plan should be established in a timely manner, and regular follow-up should be in place to ensure implementation of the plan. An assessment should be made to determine whether remediation has been successfully achieved. Even after the plan is achieved, it is important to remain vigilant about the performance of the struggling resident throughout the entirety of training | Ensuring a fair, consistent, and well-documented process is essential in getting struggling trainees back on course in their journey to become competent, independent physicians. |
| Interventions for undergraduate and postgraduate  Medical learners with academic difficulties: A  BEME systematic review: BEME Guide No. 56 | Lacasse M, Audétat MC, Boileau É, Caire Fon N, Dufour MH, Laferrière MC, Lafleur A, La Rue È, Lee S, Nendaz M, Paquette Raynard E. | 2019 | Review | NA | NA | This systematic review searched MEDLINE, CINAHL, EMBASE, ERIC, Education  Source and PsycINFO (1990-2016) combining these concepts: medical education, professional  competence/difficulty and educational support. Original research/innovation reports describing  intervention(s) for UG/PG medical learners with academic difficulties were included. Data  extraction employed Michie’s Behaviour Change Techniques (BCT) Taxonomy and program  evaluation models from Stufflebeam and Kirkpatrick. Quality appraisal used the Mixed Methods  Appraisal Tool (MMAT). The authors synthesized extracted evidence by adapting the GRADE  approach to formulate recommendations. | Clinical teachers often struggle to report unsatisfactory trainee performance, partly  because of a lack of evidence-based remediation options. | 68 articles met the inclusion criteria, most commonly addressing knowledge (66.2%),  skills (53.9%) and attitudinal problems (26.2%), or learner personal issues (41.5%). The most  common BCTs were Shaping knowledge, Feedback/monitoring, and Repetition/substitution.  Quality appraisal was inconstant (MMAT 0-100%). A thematic content analysis identified 109  interventions (UG: n=84, PG: n=58), providing 24 strong, 48 moderate, 26 weak and 11 very  weak recommendations. | This review provides a repertoire of literature-based interventions for  teaching/learning, faculty development, and research purposes. |
| Management of residents in difficulty in a Swiss general internal medicine outpatient clinic: Change is necessary! | Lanier et al. | 2021 | Qualitative | NA | 10 | We conducted an exploratory qualitative study between December 2015 and July 2016. We asked volunteers from the Primary Care Division of the Geneva University Hospitals to partake to three focus groups (with CR, A, R) and one interview with the division’s CP. We transcribed, coded, and qualitatively analyzed the three focus groups and the interview, using a content thematic approach and Fishbein’s conceptual framework. | The aim of the study was to explore the challenges perceived by physicians regarding the process of identifying, diagnosing, and supporting residents in difficulty in a structured and programmatic way. We explored perceptions of physicians at different hierarchical levels (residents (R), Chief residents (CR), attending physicians (A), Chief Physician (CP)) in order to better understand these challenges. | Supervisors (CR, A, CP) have good identification skills of residents in difficulty, but they did not put in place systematic remediation strategies.  Supervisors (CR, A) were concerned about managing residents in difficulty. They were aware of the possible adverse effects on patient care, but “feared to harm” resident’s career by documenting a poor institutional assessment.  Residents “feared to share” their own difficulties with their supervisors. They thought that it would impact their career negatively.  The four physician’s hierarchical level reported environmental constraints (lack of funding, time constraint, lack of time and resources…). | Various actions and processes have been put in place at the Faculty of Medicine in Geneva. Amongst the most important is the appointment of a task force to develop and implement an institutional remediation program for the entire medical faculty at the pre-graduate level. This makes it possible, particularly to identify learners in difficulty earlier in the course of their training. Discussions are underway for the post-graduate level. In addition, a training course consisting of several workshops and coaching over 2 years has been set up for all Chief residents. Some of the Primary care Division’s Chief residents and attending physicians are involved in this training process. After having started in 2015 in five departments, it will be extended in 2021 to all the departments of the Geneva University Hospitals receiving interns. This training will also benefit students, since it is the same Chief residents who supervise them. |
| Junior doctor remediation: an international reflection | Lessing et al. | 2019 | Perspective | NA | NA | As American medical educators with expertise in remediation, two of the authors were invited to Victoria to visit clinical sites (Monash Health and Deakin University) and to speak at the 23rd Australia and New Zealand Prevocational Medical Education Forum (ANZPMEF) in Melbourne in November 2018. Based on observations at the sites and on discussions at ANZPMEF with medical educators from universities, hospitals and medical centres throughout Australia and New Zealand, the American and Australian authors reflected on pre-vocational junior doctor remediation trends in our two regions. | Based on observations at the sites and on discussions at ANZPMEF with medical educators from universities, hospitals and medical centres throughout Australia and New Zealand, the American and Australian authors reflected on pre-vocational junior doctor remediation trends in our two regions. Medical education remediation is a global challenge, yet to the best of our knowledge, international comparisons have not been previously described. The authors aim not to judge or laud but rather to pave a path for future enquiry and collaboration. Wherever they agree elements of one system to be clearly more effective, they say so. | Terminology and consequences: Most noticeable in all our conversations in Australia was sensitivity to remediation terminology commonly used throughout US medical training; There was concern about confidentiality of junior doctor term assessments used to document the Improving Performance Action Plan; US medical training programs have worked to change a negative or punitive perception of remediation by emphasising the value of supplemental learning and support.  Learner assessments: From what we found out, compared with the US, learner assessments in Australia and New Zealand occur less frequently and are less formal; More extensive and frequent evaluation permits earlier identification of junior doctors who could benefit from remediation.  Learner supervision: Developing the skills of senior medical staff to assist in remediation of learners is a major challenge in the US, Australia and New Zealand, although greater in Australia and New Zealand; Across Australia, the increase in consultant workloads plus a reduction in full-time roles have resulted in reduced time available for supervision and teaching.  Failure to fail: The US, Australia and New Zealand share the same challenges around “failing to fail”.9,10 From nearly every conversation, it seemed that a trainee, acknowledged to be struggling, may still be advanced onto the next rotation or even complete pre-vocational training without formal recognition of any problems. The discomfort caused by formally acknowledging that someone is struggling, along with fear that once acknowledged “the problem becomes our responsibility to fix,” appears to be a universally strong driver for inaction  Greater non-medical role and support: At the visited sites, Australia and New Zealand notably used non-medical educators in more prominent medical education roles compared with the US | The authors hope that by sharing their respective approaches — in preferred terminology, evaluation and supervision, in “failing to fail”, and in the possibilities for the role of medical education officers — they take the next steps together in advancing remediation efforts and successes in both regions of the world. |
| A framework to teach self-reflection for the remedial resident | Leung & Ratnapalan | 2011 | Descriptive | NA | NA | NA | This article describes how to adapt and apply environmental scanning for remedial residents. | Environmental scanning is a rigorous and well-developed business approach that can be adapted for personal continuous quality improvement to foster self-reflection in medical trainees. There are often already existing tools which can form the foundation for regular reflection in medical education using an environmental scanning structure. | Environmental scanning can be thought of as a structured approach to internal and external reflections. |
| A theory-based curriculum design for remediation of residents’ communication skills | Leung, Martin & Batty | 2009 | Descriptive | NA | NA | NA | This article describes an experiential remedial curriculum for communication skills. Educational theories of Kolb, Knowles, Bandura, and Bloom are used to design the curriculum into theory-based design components. | Kolb's experiential cycle models the natural sequence of experiencing, teaching, and learning interviewing skills. A curriculum structured around this cycle has multiple intercalations with the above educational theories. The design is strengthened by appropriately timed use of education strategies such as learning contracts, taped interviews, simulations, structured reflection, and teacher role modeling. Importantly, it also models the form of the clinical interview format desired. | Through understanding and application of contemporary educational theories, a program to remediate interviewing skills can increase its potential for success. |
| Long‐term outcomes of a remedial education program for doctors with clinical performance deficits | Lillis et al. | 2014 | Quantitative | 12 | NA | Over the 18-month period covered in this study, 24 doctors were required by the MCNZ to enter remediation after a performance assessment. The data set used in this study was drawn from these 24 consecutive cases and included the nature of concerns, severity of concerns, results of remediation and outcome of a second assessment when such an assessment was ordered. | Medical regulatory authorities need reliable methods of assessing and remediating doctors where there are concerns over competence. There’s a small but growing literature describing remediation programs and documenting their effectiveness. This article adds to that literature by describing a program associated with the Medical Council of New Zealand (MCNZ) and reporting outcomes for 24 consecutive doctors required to undergo remediation. | Of 24 doctors who underwent initial assessment, 5 failed to engage with remediation and withdrew from clinical work. A 12-month education remediation program was completed by all remaining 19 doctors. Of these, 13 were considered to be practicing at an acceptable standard at the end of remediation on the basis of sequential supervisor reports. Six doctors were required to have a second performance assessment. Of these, only 1 was considered to be functioning at an acceptable standard. Concurrent health concerns were common among this cohort of doctors. Seventy-five percent of doctors who entered remedial education were considered to be practicing at an acceptable standard at the end of remediation. This accords well with international data. A small number of doctors appear to be unresponsive to remediation | A small number of doctors exhibit recidivist behavior despite remediation and repeatedly come to the attention of regulatory authorities. These doctors are of considerable concern and are deserving of closer study. |
| Early predictors of need for remediation  In the Australian general practice training program:  A retrospective cohort study | Magin et al. | 2016 | Quantitative | 12 | NA | We conducted a retrospective cohort study of trainees from a large Australian regionally-based GP training organization. The outcome factor was requirement for formal remediation. Independent variables were demographic factors and a range of formative assessments conducted immediately prior to or during early-stage training. Analyses employed univariate and multivariate logistic regression of each predictor assessment modality with the outcome, adjusting for potential confounders. | We sought to establish early-training assessment instruments predictive of general practice (GP) trainees' subsequently requiring formal remediation. | Of 248 trainees, 26 (10.5 %) required formal remediation. Performance on the Colleague Feedback Evaluation Tool (entailing feedback from a trainee's clinical colleagues on clinical performance, communication and probity) and External Clinical Teaching Visits (half-day sessions of the trainee's clinical consultations observed directly by an experienced GP), along with non-Australian primary medical qualification, were significantly associated with requiring remediation. There was a non-significant trend for association with performance on the Doctors Interpersonal Skills Questionnaire (patient feedback on interpersonal elements of the consultation). There were no significant associations with entry-selection scores or formative exam or assessment scores. | Our finding that 'in vivo' assessments of complex behaviour, but not 'in vitro' knowledge-based assessments, predict need for remediation is consistent with theoretical understanding of the nature of remediation decision-making and should inform remediation practice in GP vocational training. |
| Remedial training for the  radiology resident:  a template for optimization of the learning plan | Mar et al. | 2015 | Interventional | NA | NA | This article provides a template for a step-by-step approach which is team based. | A customized plan for guidance, learning, and assessment must then be developed for each individual’s specific needs. Resident participation is a key aspect of the planning. In addition to possibly revealing other factors contributing to the resident’s difficulty, it will help affirm that the primary goal of the program is to assist the resident. Such an endeavor may quickly prove daunting in the face of particular learning | With the considerable academic and clinical challenges and diversity of trainees, each radiology residency program should be prepared for the eventuality of the resident in need of remediation. A step-by-step approach is detailed here and also summarized in a template. At our institution, all of these components are compiled in a document, the Remediation Plan, by the remedial supervisor. This must then be reviewed by the resident, program director, and Associate Dean, Postgraduate Medicine, to allow for any necessary modifications. Following consensus, the document is signed by all four individuals. The team-based approach to the process is thereby strengthened. Trainees and faculty have both described the plan as rigorous, but staff participation has been very favourable, whereas the learners have expressed appreciation for the dedicated teaching. This collaboration, combined with a customized and comprehensive plan, will optimize the transition of the resident in difficulty into a safe and competent radiologist and valued colleague. | With the considerable academic and clinical challenges and diversity of trainees, each radiology residency program should be prepared for the eventuality of the resident in need of remediation. A step-by-step approach is detailed here and also summarized in a template. At our institution, all of these components are compiled in a document, the Remediation Plan, by the remedial supervisor. This must then be reviewed by the resident, program director, and Associate Dean, Postgraduate Medicine, to allow for any necessary modifications. Following consensus, the document is signed by all four individuals. The team-based approach to the process is thereby strengthened. Trainees and faculty have both described the plan as rigorous, but staff participation has been very favourable, whereas the learners have expressed appreciation for the dedicated teaching. This collaboration, combined with a customized and comprehensive plan, will optimize the transition of the resident in difficulty into a safe and competent radiologist and valued colleague. |
| Assessing the prescribing skills of trainee medical staff: implementation of a routine assessment and remedial training strategy | Mayne et al. | 2010 | Quantitative | 13 | NA | Trainees attending induction in a large NHS foundation hospital participated in a compulsory clinical assessment of prescribing skills. Trainees were presented with a clinical scenario from which they were required to prescribe specified drugs on a hospital prescription chart. A consensus panel marked the station according to pre-specified ‘critical’ errors or omissions. Candidates who made critical errors or omissions were invited to remedial training and reassessment. | To describe the process and outcomes of a routine assessment of prescribing and remedial training for newly appointed medical trainees. | In total 120 trainees were assessed, of whom 72.5% (87/120) made critical errors or omissions. Subsequently, 79.3% (69/87) of trainees were reassessed; 79.7% (55/69) of whom passed on 2nd attempt, and 78.6% (11/14) passed on 3rd attempt; 3 doctors did not attend further reassessment. The most common critical errors were: prescription of the wrong dose of warfarin (59.2%); failure to stop aspirin (44.6%); and inappropriate abbreviation of ‘units’ when prescribing insulin (39.8%). 47 candidates (38.8%) prescribed amoxicillin in a penicillin allergic patient. Foundation Year 1 doctors performed worse than Senior House Officer (SHO) and Specialist Registrar (SPR) doctors (Mann-Whitney test, p<0.001, p=0.12 respectively) and made more critical errors (p=0.003 and p=0.32). It cannot be assumed that newly appointed doctors prescribe safely. Critical errors or omissions were made by all grades of medical staff, but particularly Foundation Year 1 doctors. Steps to improve exposure to prescribing practice in the undergraduate curriculum should be encouraged and linked to ongoing development of skills throughout practice. | Although considerable emphasis is now given to the teaching of prescribing skills in undergraduate and UK Foundation Medical School curricula, it is clear that a more integrated strategy is required across the UK National Health Service to standardise the way electronic prescribing is implemented and to reduce local variations in the use of non-standard prescribing processes for common, but potentially dangerous, drugs. |
| Orthopaedic resident remediation  frequency, interventions, and outcomes | Melton et al. | 2018 | Quantitative | 15.5 | NA | The program directors of all current 159 Accreditation Council for Graduate Medical Education-recognized orthopaedic residency programs in the United States were e-mailed a non-identifying digital survey. Follow-up e-mails were sent monthly for a 3-month period. The data remained anonymous, with no identifying information. The data are reported with use of descriptive statistics. | Despite multiple studies in other areas of surgical resident education, there is no current literature regarding orthopaedic resident remediation. The goal of the present study was to use a survey format to determine the frequency of remediation, the underlying etiology, the intervention strategies utilized, and the outcomes. | Seventy (44%) of 159 program directors responded; most were from academic institutions with 4 to 5 residents per class. One hundred and fifty-eight residents were remediated, with the greatest number being remediated during the third postgraduate year (PGY-3). Professionalism, patient care, and communication were the most common deficiencies requiring remediation. Mentorship, feedback, and probation were the most common interventions. Of the 117 residents for which the outcome was reported, 58 graduated on time, 14 graduated from another program, 25 graduated from another specialty, 14 were terminated, pursued litigation, and 3 chose a non-medical career. Rehabilitation, didactics, feedback, and mentorship were associated with the highest rates of on-time graduation. | Most remediated residents were PGY-3, suggesting increased scrutiny as residents moved from junior to senior responsibilities. The greatest number of deficiencies requiring remediation pertained to the affective domain, which highlights the importance of screening measures such as away rotations and interviews. The relationships formed during increased feedback sessions and mentorships can help problem residents to graduate on time. |
| Can this resident be saved? Identification and early intervention for struggling residents | Minter et al. | 2014 | Descriptive | NA | NA | A  panel of experts was convened and asked to present at  the American College of Surgeons Clinical Congress a  strategy focused on early identification of these residents,  development of targeted interventions, and mechanisms  for setting expectations for resolution of the identified  deficit or issue.  Common deficiencies or issues impacting  performance were specifically targeted for discussion, and  this article highlights the experts’ recommendations  within the domains of operative deficiency, organizational  inefficiency, substance abuse, and poor clinical judgment. | The American College of Surgeons Committee on  Resident Education within the Division of Education  sought to define common domains of deficiency  frequently observed in struggling surgical residents. | Improved diagnostics are clearly required for early identification  and intervention for residents who are struggling  with various aspects of performance during surgical residency.  Historically, these deficiencies have gone unrecognized  and untreated, resulting in the potential graduation  of a resident who is ill prepared for safe independent practice,  or termination of a resident who had a deficiency that  could have been effectively remediated during residency. | The rubrics presented by this expert panel of surgical educators  provide a framework for developing diagnostics to  determine what the exact cause of a resident’s deficiency  might be within common domains that are critical to a  surgeon’s development. As we move more explicitly into  a model of competency-based assessment for progression  during residency, the ability to identify, assess, and treat  identified deficiencies in our residents’ performance will  be required and documented as they progress through  the Milestones. |
| Remediation strategies for emergency medicine patient care milestones | Murano, Smith & Weizberg | 2018 | Descriptive | NA | NA | NA | Objective - to create tools for identifying and remediating residents with deficiencies in patient care milestones (PCMs). | Among the challenges of remediation, PDs have difficulty with identifying residents in need of remediation, diagnosing the cause of their underlying deficiencies, and remediating them. Residents failing to meet expectations may be identified in several ways, including the review of end-of-rotation evaluations, CCC meeting assessments, or a resident’s semi-annual review. However, waiting to uncover issues during infrequently scheduled evaluations may lead to a delay in the identification of deficiencies. | EM program leadership can use the PCM-mSDOTs to identify resident strengths and areas for improvement, track resident progress, and initiate remediation plans. The PCM remediation rubric may a useful tool to formulate an individualized remediation plan for any resident with deficiencies at various milestone levels |
| Simulation-based remediation in emergency medicine  residency training: a consensus study | Nadir et al. | 2019 | Review | NA | NA | We conducted a literature search on SBR practices using the terms “simulation,” “remediation,” and “simulation based remediation.” We identified relevant themes and used them to develop an open-ended questionnaire that was distributed to EM programs with experience in SBR. Thematic analysis was performed on all subsequent responses and used to develop survey instruments, which were then used in a modified two-round Delphi panel to derive a set of consensus statements on the use of SBR from an aggregate of 41 experts in simulation and remediation in EM. | With the exception of SBML and procedural remediation,9 there remains no clear consensus on when and how to appropriately use simulation for remediation in EM for other sub-competencies. The lack of specific recommendations and guidelines makes SBR planning difficult for both residency program and simulation leadership. To answer this need, the CORD-EM Simulation Community of Practice (COP), CORD-EM RTF and the SAEM Simulation Academy formed a joint collaboration, the Simulation Based Remediation Collaborative (SBRC), to clarify the role of SBR. This study was based on their work, and its objective was to build consensus on the appropriate use of SBR in EM. | Faculty representing 30 programs across North America composed the consensus group with 66% of participants identifying themselves as simulation faculty, 32% as program directors, and 2% as core faculty. The results from our study highlight a strong agreement across many areas of SBR in EM training. SBR is appropriate for a range of deficits, including procedural, medical knowledge application, clinical reasoning/ decision-making, communication, teamwork, and crisis resource management. Simulation can be used both diagnostically and therapeutically in remediation, although SBR should be part of a larger remediation plan constructed by the residency leadership team or a faculty expert in remediation, and not the only component. Although summative assessment can have a role in SBR, it needs to be very clearly delineated and transparent to everyone involved. | Simulation can be used diagnostically as well as therapeutically in remediation processes. Once a deficit is identified, simulation can be a helpful remediation tool for certain competencies and milestones, but simulation is not a one-size-fits-all approach that can be applied to every EM skill or competency. Simulation is best suited for remediation of procedural, patient care and communication milestones and less suited for remediation of systems-based practice and problem-based learning milestones. SBR can be one aspect, but should not be the sole component of a remediation plan. Similarly, SBR performance should only be one component of how remediation success is assessed by program leadership and the CCC, using tools with validity evidence when possible. These SBR assessments should be transparent between the simulation faculty, the learner, the program leadership and the CCC. |
| Residents in difficulty—just slower learners? A case–control study | O’Neill et al. | 2014 | Qualitative | 10 | NA | The study design was a cumulative incidence matched case–control study. The source population was  all active specialist trainees, who were medical school graduates from Aarhus University, in 2010 to June 2013 in  two Danish regions. Cases were doctors who decelerated, transferred, or dropped out of residency. Cases and  controls were matched for graduation year. Medical school exam failures, grades, completion time, and academic  dispensations as predictors of case status were examined with conditional logistic regression. | The aim of this  study was to examine if a sample of Danish residents in difficulty tended to struggle already in medical school, and  to determine whether administratively observable performance indicators in medical school could predict difficulties  in residency. | In total 89 cases and 343 controls were identified. The total number of medical school re-examinations  and the time it took to complete medical school were significant individual predictors of subsequent difficulties  (deceleration, transferral or dropout) in residency whereas average medical school grades were not. | Residents in difficulty eventually reached similar competence levels as controls during medical  school; however, they needed more exam attempts and longer time to complete their studies, and so seemed to  be slower learners. A change from “fixed-length variable-outcome programmes” to “fixed-outcome variable-length  programmes” has been proposed as a way of dealing with the fact that not all learners reach the same level of  competence for all activities at exactly the same time. This study seems to support the logic of such an approach  to these residents in difficulty. |
| Developing a physician's professional identity through medical education | Olive & Abercrombie | 2017 | Descriptive | NA | NA | Irby and Hamstra outlined  three medical education frameworks for addressing professionalism: one based on virtues, one  on behaviors, and one on professional identity formation. We will approach this issue primarily  from a professional identity formation perspective. | Professionalism represents a fundamental characteristic of physicians. Professional  organizations have developed professionalism competencies for physicians and medical  students. The aim of teaching medical professionalism is to ensure the development of a  professional identity in medical students. Professional identity formation is a process  developed through teaching principles and appropriate behavioral responses to the stresses of  being a physician. Addressing lapses and critical reflection is an important part of the  educational process. | The "hidden curriculum" within an institution plays an important role in professional identity formation. Assessment of professionalism involves multiple mechanisms. | Steps in remediating professionalism lapses include (1) initial assessment, (2) diagnosis of problems and development of an individualized learning plan, (3) instruction encompassing practice, feedback and reflection and (4) reassessment and certification of competence. No reliable outcomes data exist regarding the effectiveness of different remediation strategies. |
| Evolution of a Remedial CME Course in Professionalism:  Addressing Learner Needs, Developing Content, and  Evaluating Outcomes | Parran, Jr et al. | 2013 | Quantitative | 11.5 | NA | The medical ethics course has been designed in consultation with licensure agencies to address the learning needs of physicians with problems in the areas of boundary maintenance and ethics. Teaching methods and outcome evaluations include lectures, case discussions, multiple-choice question tests, skill practice sessions, and writing a reflective essay based on the participants' ethical lapse. Information is also gathered regarding participant demographics, training, and practice characteristics. | Scant information is available about the nature of the professional violations resulting in referral of physicians for remedial continuing medical education (CME). The CME program at Case Western Reserve University (CWRU) School of Medicine has developed the Intensive Course in Medical Ethics, Boundaries, and Professionalism (medical ethics course) for physician referrals due to ethical breaches. In this report, the authors present 7 years of data regarding the type of behavior that resulted in course referral as well as information regarding course and outcome evaluation development and participant demographics. | Between September 2005 and February 2012, 358 learners participated in the course. The average age was 52 years and 73% were board certified. Of the 269 physicians who wrote a reflective essay, the reasons for referral included prescribing of controlled drugs, sexual boundary issues, providing services to family or friends, not maintaining proper medical records, and billing issues. | This report outlines the strategies used by CWRU to develop remedial CME courses using the medical ethics course as an example for course and outcome evaluation development. This is the first report characterizing the type and frequency of the medical ethics violations that result in mandatory participation in remedial CME. |
| A novel educational intervention to increase clinical scholarship among faculty and trainees | Penzner et al. | 2015 | Review | NA | NA | We performed a literature review and a departmental needs assessment survey to identify gaps in knowledge and training. Based on our findings we developed and implemented a comprehensive educational program for faculty and trainees consisting of five core components: (1) didactic series on clinical scholarship, (2) semi-structured academic mentorship (3) biweekly writing seminar, (4) requirement for scholars to present regularly on goals and progress, and (5) lunch meetings to support idea sharing and collaboration. Using input from educational research experts, established surveys, and literature review, we developed web-based questionnaires to examine (1) what similar activities are in place around the country, and (2) which aspects of our CSI are most generative of academic progress. We created record keeping for scholars’ progress and faculty’s interventions to track measurable outcomes (e.g. grants, awards, papers, presentations). We will review academic life post-residency for these trainees to elucidate effectiveness of the CSI long-term, and will also compare the current group of trainee participants with past trainees who did not engage with such an Institute. | The aim of this project is to introduce this model for clinical scholarship, with discussion of its rationale, development and mechanism, and explanation of program evaluation results. A secondary aim is to elucidate the most successful components of the CSI to enhance productivity and to demonstrate generalizability. | Program evaluation data are being collected and will be reported. | A CSI creates an infrastructure for these developments and a community generative of academic progress. It offers a model for an innovative educational program with demonstrated efficacy in fostering scholarship in the Weill Cornell Department of Psychiatry. Clinical scholarship maintains the health of the field and its academic institutions, particularly as the current cost-driven climate threatens to undermine academic identity, idea sharing and career-oriented self-examination. |
| Managing residents in difficulty within CBME residency educational systems: a scoping review | Pirie et al. | 2020 | Descriptive | NA | NA | Articles published between 2011 to 2017 were included if they were about postgraduate medical education, RID, and offered information to inform the structure and/or processes of CBME. All three reviewers performed a primary screening, followed by a secondary screening of abstracts of the chosen articles, and then a final comprehensive sub-analysis of the 11 articles identified as using a CBME framework. | Best practices in managing residents in difficulty (RID) in the era of competency-based medical education (CBME) are not well described. This scoping review aimed to inventory the current literature and identify major themes in the articles that address or employ CBME as part of the identification and remediation of residents in difficulty. | Of 165 articles initially identified, 92 qualified for secondary screening; the 63 remaining articles underwent full-text abstracting. Ten themes were identified from the content analysis with “identification of RID” (41%) and “defining and classifying deficiencies” (30%) being the most frequent. In the CBME article sub-analysis, the most frequent themes were: need to identify RID (64%), improving assessment tools (45%), and roles and responsibilities of players involved in remediation (27%). Almost half of the CBME articles were published in 2016–2017. | Although CBME programs have been implemented for many years, articles have only recently begun specifically addressing RID within a competency framework. Much work is needed to describe the sequenced progression, tailored learning experiences, and competency-focused instruction. Finally, future research should focus on the outcomes of remediation in CBME programs |
| Early detection of factual knowledge deficiency and remediation  in otolaryngology residency education | Platt et al. | 2014 | Quantitative | 15.5 | NA | For each didactic lecture within a single otolaryngology residency training program, multiple choice questions were provided before and after each lecture. Questions were based on lecture objectives that were derived from the American Board of Otolaryngology curriculum. Scores were tracked over the course of 1 academic year and compared to the scores of residents on the OTE administered in that year to determine correlation with a validated measure of factual knowledge. The effect of remedial measures on improvement in OTE scores was determined. | The objective of this study was to develop a more frequent method for tracking of factual knowledge to prevent educational delay. | Over the course of 1 academic year, there were 328 questions presented to 12 residents before and after 32 lectures in the didactic program. Ten residents completed an average of 244 questions. Overall OTE scores demonstrated a significant and very strong correlation to lecture question scores (Pearson r = 0.86, P = .002). Remedial measures for residents during the previous 5 years who had inadequate OTE scores were successful in improving scores (P = .002). | A structured didactic program that uses review questions to assess knowledge can be used to track acquisition of factual knowledge. Early identification of residents with deficiencies facilitates the development of individualized learning plans that result in successful remediation. |
| Identifying the at-risk General Practice trainee: a retrospective cohort meta-analysis of General Practice registrar flagging | Prentice et al. | 2021 | Review | NA | NA | Informed by unstructured interviews with Senior Medical Educators to understand the fagging system of each RTO, meta-analyses of routinely-collected fagging data were used to examine the predictive validity of fagging at various points in training and exam performance. Additionally, fagging system features identified from the interviews were used to inform exploratory subgroup analyses and meta-regressions to further assess the predictive and consequential validity of these systems. | The present study attempts to address this gap by examining the predictive and consequential validity of fagging systems used by Australian General Practice regional training organisations (RTOs) in relation to Fellowship examinations. | Registrars fagged near the end of their training were two to four times more likely to fail Fellowship exams than their non-fagged counterparts. Regarding fagging system features, having graded (i.e. ordinal) fagging systems was associated with higher accuracy, whilst involving the assigned medical educator in remediation and initiating a formal diagnostic procedure following a fag improved registrars’ chances of passing exams. | These results demonstrate both predictive and consequential validity of fagging systems. We argue that fagging is most effective when initiated early in training in conjunction with mechanisms to maximise diagnostic accuracy and the quality of remediation programs. |
| Rethinking remediation: a model to support the  detailed diagnosis of clinicians’ performance  problems and the development of effective  remediation plans | Prescott-Clements et al. | 2017 | Mixed | 8 | 9 | The model we developed has three stages: Stage 1: Developing the questions for semi-structured discussions with individual clinicians and their employer. Stage 2: Data collection 2a. Interview with the clinician 2b. Semi-structured discussion with the employer Stage 3: Triangulation and development of the remediation program | This article describes the development of a new model for the development of remediation action plans for clinicians with performance problems based on established theory, and the rationale for this approach based on the evidence available. We began by reviewing the medical education literature to explore the existing approaches to remediation, reported effectiveness, and underlying theories these were based upon. Then, we considered the nature of performance problems of clinicians accessing NCAS’ support in order to identify appropriate theories on which to base our approach, with the aim of maximizing effectiveness in terms of achieving long-term, improved, clinical performance in the workplace. | NA | The next stage of this work is to perform a longitudinal evaluation of the model to determine the effectiveness of the method and impact upon the remediation of clinicians. Through systematic follow-up and evaluation of construct data, we will attempt to identify the constructs most likely to predict successful—or unsuccessful—remediation. |
| Remediating doctors’ performance to  restore patient safety: a realist review  protocol | Price et al. | 2018 | Review | NA | NA | Realist review is an approach to evidence synthesis that seeks to develop programme theories about how an intervention works to produce its effects. The initial search strategy will involve: database and grey literature searching, citation searching and contacting authors. The evidence search will be extended as the review progresses and becomes more focused on the development of specific aspects of the programme theory. The development of the programme theory will involve input from a stakeholder group consisting of professional experts in the remediation process and patient representatives. Evidence synthesis will use a realist logic of analysis to interrogate data in order to develop and refine the initial programme theory into a more definitive realist programme theory of how remediation works. The study will follow and be reported according to Realist And Meta-narrative Evidence Syntheses—Evolving Standards (RAMESES). | The aim of this research is to identify why, how, in what contexts, for whom and to what extent remediation programmes for practising doctors work to support patient safety. | During the review, we shall move iteratively between the analysis of particular examples, refinement of the programme theory and further iterative searching for data to test particular theories. The final realist programme theory will be presented in a diagram and through a narrative description of CMOCs. | The proposed research will make an empirical contribution to the existing body of knowledge by developing a transferable realist programme theory of how remediation of doctors works, for whom and in what contexts. Achieving this type of understanding will also enable us to develop recommendations to support the optimal tailoring, design and implementation of remediation interventions for underperforming doctors in order to support patient safety. This research will generate new knowledge about a poorly understood area of healthcare delivery that directly affects the standards of care received by patients. It is thus consistent with a focus on improving the quality and the organisation of health services, in this instance within the specific area of improving the design and delivery of remediation programmes |
| Optimising the delivery of remediation programmes for doctors: a realist review | Price et al. | 2021 | Review | NA | NA | Medical underperformance puts patient safety at risk. Remediation, the  process that seeks to ‘remedy’ underperformance and return a doctor to safe practice, is therefore a crucially important area of medical education. However, although  remediation is used in health care systems globally, there is limited evidence for the  particular models or strategies employed. The purpose of this study was to conduct a  realist review to ascertain why, how, in what contexts, for whom and to what extent  remediation programmes for practising doctors work to restore patient safety | We conducted a realist literature review consistent with RAMESES standards.  We developed a programme theory of remediation by carrying out a systematic search  of the literature and through regular engagement with a stakeholder group. We searched  bibliographic databases (MEDLINE, EMBASE, PsycINFO, HMIC, CINAHL, ERIC, ASSIA  and DARE) and conducted purposive supplementary searches. Relevant sections of text  relating to the programme theory were extracted and synthesised using a realist logic of  analysis to identify context–mechanism–outcome configurations (CMOcs) | A 141 records were included. The majority of the studies were from  North America (64%). 29 CMOcs were identified. Remediation programmes are  effective when a doctor's insight and motivation are developed and behaviour  change reinforced. Insight can be developed by providing safe spaces, using  advocacy to promote trust and framing feedback sensitively. Motivation can  be enhanced by involving the doctor in remediation planning, correcting causal  attribution, goal setting and destigmatising remediation. Sustained change  can be achieved by practising new behaviours and skills, and through guided  reflection | Remediation can work when it creates environments that trigger behaviour change mechanisms. Our evidence synthesis provides detailed recommendations on tailoring implementation and design strategies to improve remediation  interventions for doctors. |
| Remediation in the context of the  competencies: a survey of pediatrics  residency program directors | Riebschleger &  Haftel | 2013 | Quantitative | 6.5 | NA | We obtained data via an anonymous online survey of paediatrics residency program directors. For the purposes of the survey, remediation was defined as ‘‘any form of additional training, supervision, or assistance above that required for a typical resident.’’ Respondents were asked to quantify 3 groups of residents: (1) residents requiring remediation; (2) residents whose training was extended for remediation purposes; and (3) residents whose training was terminated owing to issues related to remediation. For each group, the proportion of residents with deficiencies in each of the 6 competencies was calculated. | The goal of this article is to describe the degree to which each of the competencies contributed to deficiencies requiring remediation. | In all 3 groups, deficiencies in medical knowledge and patient care were most common; deficiencies in professionalism and communication were moderately common; and deficiencies in systems-based practice and practice-based learning and improvement were least common. Residents whose training was terminated were more likely to have deficiencies in multiple competencies. | Although medical knowledge and patient care are reported most frequently, deficiencies in any of the 6 competencies can lead to the need for remediation in pediatrics residents. Residents who are terminated are more likely to have deficits in multiple competencies. It will be critical to develop and refine tools to measure achievement in all 6 competencies as the graduate medical education community may be moving further toward individualized training schedules and competency-based, rather than time-based, training |
| Twelve tips for addressing medical student and resident physician lapses in professionalism | Rougas et al. | 2015 | Descriptive | NA | NA | To shed light on this important issue and generate a shared institutional model, a half-day seminar entitled ‘‘Professionalism in Medical Education’’ was held in September 2013 at the Alpert Medical School of Brown University (AMS) in Providence, Rhode Island (USA) as part of its Program in Educational Faculty Development. A 90-min workshop was preceded by a keynote address given by a nationally known expert in physician professionalism. Over 50 faculty members attended both sessions to discuss and share experiences related to addressing lapses in professionalism. The 12 tips in this article are a summary of the points discussed in that workshop and are provided here as a guide for those seeking specific, practical steps when undertaking formal and informal discussions with learners. | This article reviews the literature related to addressing unprofessional behavior among trainees in medicine and connects it to the shared experience of medical educators at one institution. The framework presented aims to provide practical guidance and empowerment for educators responsible for addressing medical student and resident physician lapses in professionalism. | The tips are organized into four sections: building a healthy organization (tips 1–3), data gathering and fact checking (tips 4–6), having the conversation (tips 7–9), and closing the loop (tips 10–12). With each tip, we have provided potential phrasing (Table 1) to help guide the discussion with learners (and when necessary colleagues) at each step. | Thoughtful and well executed plans for addressing lapses in professionalism can have a positive impact on medical students and resident physicians, and it is our hope that the strategies outlined here will assist medical educators in achieving this goal. |
| Recognizing residents with a deficiency  in operative performance as a step closer  to effective remediation | Sanfey et al. | 2013 | Perspective | NA | NA | NA | As educators, we need to ensure that our trainees are ready for independent practice before graduation, but with reductions in resident work hours, all residents are spending less time in the operating room18 and performing fewer cases than program directors consider desirable.19 Almost certainly the increasing number of residents who are choosing to do post residency fellowship training reflects the residents’ own concern about their readiness for independent practice. Therefore, the need to develop a systematic approach to evaluating operative performance and recognizing and remediating DOP is apparent. | See discussion | As a first step toward developing a taxonomy, we have presented a framework of forward planning, self-direction, situation awareness, and patient safety/judgment for categorizing nontechnical DOP to facilitate targeted remediation. This approach has limitations, there is some overlap in these categories and they were developed from an observation of videotaped performance by experts blinded to the history and year of training of the resident. The attending surgeon in the operating room who knows the resident, and can place deficiencies into context with case complexity, resident history, and year of training, might categorize these deficits in a different way. Although the remediation strategies outlined here will be expensive in faculty time commitment, the cost to our patients is far greater if they are not implemented, and we graduate residents who are not able to practice safely in independent practice. As a next step we will develop and validate a more detailed taxonomy to facilitate specific recognition and targeted remediation strategies for DOP. In the longer term, additional research will be needed to determine whether or not the remediation strategies outlined here are in fact effective. |
| Developing a modern standard to define and assess professionalism in trainees | Schwartz, Kotwicki & McDonald | 2009 | Review | NA | NA | The authors review the development of standards of professionalism in medical education. They define educational goals for professionalism and also discuss the practical problems with assessing professionalism and addressing it with the trainees. Strategies for remediation of unprofessional conduct are outlined | Assessing professionalism in medical education poses many challenges. The authors discuss common themes and principles in managing professionalism in medical education. | Given the importance of role models in the development of professional behavior, maintaining an environment that fosters professionalism is an implicit feature of teaching professionalism. Professionalism should be a part of the objectives for each course and clinical rotation, using clearly defined goals and objectives. Assessment of professionalism should begin early and be conducted frequently, giving trainees the opportunity to change. A formal mentoring system can be an effective mechanism to develop role models and teach professionalism. | For these models to be effective, other variables that initially appear to be indirectly related to training professionalism must also be bolstered. First, senior faculty and other staff must also be held to high professional standards, because modeling behaviors is a powerful teaching tool for trainees. Second, a low threshold for completing a professionalism report must be expected and promoted across all training sites. Having protected teaching time and time for face-to-face feedback is essential for making this strategy successful. Next, PPCs need to make certain that recommendations for remediation of unprofessional behaviors match the origin of the behaviors. Trainees with illnesses or personal problems must be referred to treatment, not disciplined; popular trainees must be held to consistent standards that are the same as for their colleagues. Finally, trainees deserve formative as well as evaluative feedback about their professionalism so that they will be able to absorb feedback without constant fear of grading retribution |
| Association of general surgery resident remediation  and program director attitudes with resident attrition | Schwed | 2017 | Descriptive | NA | NA | This multicentre study surveyed 21 US program directors in general surgery about their opinions regarding resident education and attrition. Data on total resident complement, demographic information, and annual attrition were collected from the program directors for the study period of July 1, 2010, to June 30, 2015. The general surgery programs were chosen on the basis of their geographic location, previous collaboration with some coauthors, prior work in surgical education and research, or a program director willing to participate. Only categorical surgical residents were included in the study; thus, program directors were specifically instructed to exclude any preliminary residents in their responses. | To determine whether program director attitudes are associated with resident attrition and to measure the categorical resident attrition rate. | The overall 5-year attrition rate of 8.8% was significantly lower than previously reported. Program directors at low-attrition programs were more likely to use resident remediation. Variations in attrition may be explained by program director attitudes, although larger studies are needed to further define program factors affecting attrition. | Larger studies are needed to further define program factors affecting attrition. |
| Remediation in Canadian medical residency  programs: established and emerging best  practices | Shearer et al. | 2018 | Descriptive | NA | NA | We conducted a literature review to identify best practices in the area of remediation. We then reviewed remediation policies from all 13 English medical schools in Canada other than our own and conducted interviews with key informants from each institution. Each policy and interview transcript pair was then reviewed for evidence of pre-defined “best practices.” Team members also noted additional potential policy or process enablers of successful remediation | We sought to (1) review existing literature in order to ascertain “best practices” for remediation in postgraduate medical education; (2) examine the degree of concordance between current remediation policies and practices at Canadian medical schools and published best practices; and (3) highlight policy or procedural factors that can optimize or impede remediation processes. | Most policies and processes aligned with some but not all published best practices. For instance, all participating schools tailored remediation strategies to individual resident needs, and a majority encouraged faculty-student relationships during remediation. Conversely, few required the teaching of goal-setting, strategic planning, self-monitoring, and self-awareness. In addition, we identified avoidance of automatic training extension and the use of an educational review board to support the remediation process as enablers for success. | A best practice identified in addition to those suggested by Kalet et al. (2014) recommended flexibility in determining whether remediation should result in an extension of training. The traditional view that a period of remediation is “in addition” to regular training prolongs residency, and can cause a number of challenges for residents, departments and post-graduate administration. For residents, finishing later than their colleagues in the program can be distressing and challenging to their self esteem (Hurst et al. 2016). For departments, finding space for residents who require additional/extra training in addition to their regular resident complement can be challenging. For postgraduate administration, keeping track of changes to end-dates related to remediation requests can add to the administrative burden associated with remediation. Some of these challenges may be avoided by allowing remediation that is concurrent with regular training when possible and educationally appropriate. Another additional best practice identified in our analysis recommended the use of an arm’s length Educational Advisory Board (EAB). In addition to minimizing conflicts of interest, development of EABs may serve to develop expertise in remediation and improve both the efficiency and effectiveness of remediation processes. Our key informants highlighted the utility of the EAB in the approval of proposed remediation plans, both for the faculty who develop them, and for the residents who participate in them. Some implied a legal advantage, and potentially fewer appeals for the institution when responsibility for review and approval of the plans rested with a centralized, experienced committee, as compared with individual programs. |
| What is the prevalence and success of remediation of emergency medicine residents? | Silverberg et al. | 2015 | Quantitative | 13.5 | NA | We developed the survey in Surveymonkey™ with attention to content and response process validity. EM program directors responded how many residents had been placed on remediation in the last three years. Details regarding the remediation were collected including indication, length and success. We reported descriptive data and estimated a multinomial logistic regression model. | The primary objective of this study was to determine the prevalence of remediation, competency domains for remediation, the length, and success rates of remediation in emergency medicine (EM). | We obtained 126/158 responses (79.7%). Ninety percent of programs had at least one resident on remediation in the last three years. The prevalence of remediation was 4.4%. Indications for remediation ranged from difficulties with one core competency to all six competencies (mean 1.9). The most common were medical knowledge (MK) (63.1% of residents), patient care (46.6%) and professionalism (31.5%). Mean length of remediation was eight months (range 1-36 months). Successful remediation was 59.9% of remediated residents; 31.3% reported ongoing remediation. In 8.7%, remediation was deemed “unsuccessful.” Training year at time of identification for remediation (post-graduate year [PGY] 1), longer time spent in remediation, and concerns with practice-based learning (PBLI) and professionalism were found to have statistically significant association with unsuccessful remediation. | Future directions might prospectively identify a cohort of residents on remediation and examine the overlap of domains, determine methods of successful remediation and risk factors associated with failure to remediate |
| Understanding professional development: case studies of remedial support | Skelton, Wiskin & Ward | 2019 | Qualitative | NA | 10 | This study reports on work undertaken by the Interactive Studies Unit (ISU), University of Birmingham. A total of 727 doc- tors were referred to the ISU for one-to-one remedial support in a variety of non-clinical areas between 2010 and 2018. | This study, therefore, looks at a variety of cases where the authors, as members of the Interactive Studies Unit (ISU), have been involved in offering remedial, confidential support. Our aim is to offer a description of our experiences and in particular to describe the overlap between values and behavior, as this presents in particular cases. | The basic taxonomy of referrals the ISU works with, and which echoes those developed elsewhere, considers problems as being at the level of the self, interactions with others, or working in an institutional or societal context. Six common generic problems are identified, and presented and discussed as generic cases. These are designed to be representative of the complex manner in which behavior and values interact, and problems at the three levels above impinge on each other. All cases are accompanied by details of suggested educational activities. | Since most students and junior doctors do not have these difficulties, we do not suggest prohibitively expensive one-to-one support for all. Our experience highlights problems that learners may have which offer “early warning” of later difficulty. These can be an accumulation of purportedly lower stakes events like frequently being “a little bit late,” infrequently initiating in small groups, poor planning skills, tendency to externalize problems, rambling responses, and a fall back on “I have high standards” as an explanation for the alienation of others. Recognizing—and acting on—commonly presenting signs early on helps with preemptive support. |
| Defining uniform processes for remediation, probation and  termination in residency training | Smith | 2017 | Descriptive | NA | NA | To achieve consensus and external content validity of our model, we conducted semi-structured interviews of seven PDs from different programs and five designated institutional officers (DIOs) or deans of graduate medical education (GME) from five institutions. Interviews began with open-ended questions to allow PDs, DIOs, and the GME office to describe their processes for remediation, including probation and termination. They were then asked to provide definitions for each of the four domains identified within our model. Themes were abstracted and compared with our definitions. | Establishing shared and consistent definitions of remediation processes will enable training programs to achieve the goals intended by the ACGME, hold the medical profession accountable, and will further engender the public trust.[1](https://www.ncbi.nlm.nih.gov/pmc/articles/PMC5226740/#b1-wjem-18-110) Although categorizing specific resident deficiencies is beyond the scope of this paper, by incorporating the remediation practices from multiple specialties and identifying the common threads, we provide guidelines for the remediation process independent of the medical specialty. | While we noted minor variances regarding how programs or institutions applied different aspects of the remediation process, the central definitions and sequence followed were consistent with our model. | There is significant variation among programs regarding definitions and processes of remediation, probation, and termination.[16](https://www.ncbi.nlm.nih.gov/pmc/articles/PMC5226740/#b16-wjem-18-110) We provide a consensus framework for defined triggers, associated documentation, and disclosure practices. If remediation is not adequately documented and a clear process is not followed, this can hamper and affect the outcomes of formal grievance processes; thus, this schema has a standardized component to avoid that pitfall. |
| A general framework for approaching residents in difficulty | Smith et al. | 2007 | Descriptive | NA | NA | The authors describe a proposed general framework for approaching residents in difficulty. | The general framework we describe in this paper has evolved from the combined experience of the authors who, in their administrative capacities, have dealt with many types of difficulties in three specialties and in  several training programs. This framework is supported  by relevant literature and, in the authors’ experience, it has stood the test of administrative and legal challenges. The three steps in the framework—preparing for difficulties, categorizing difficulties, and responding to difficulties—are discussed. | Each resident’s difficulty within a given training program is unique. A general framework such as we have  described can only provide guidance for the program’s  specific response to the difficulty. However, we have  found that the structure provided by this framework has helped the program to organize and streamline their  response to the resident with difficulties and to avoid  missing critical elements in that response. | The critical first element of this framework is the development of graduated learning objectives and essential job functions before dealing with learner difficulties. This activity by the faculty responsible for resident training often reveals differences in basic values and styles. It is useful to take the time  to negotiate these differences before the pressure of an actual resident in difficulty adds haste and divisiveness  to the proceedings. The second critical element of this framework is to make a distinction between different types of difficulties (competence, laws and professional standards, and  performance and disability) because they are handled  very differently. |
| Individualized remediation during  fellowship training | Sparks et al. | 2016 | Quantitative | 14 | NA | The Accreditation Council for Graduate Medical Education requires medical training programs to monitor, track, and formally document a fellow's performance. If deficiencies are found, programs are expected to prepare and implement an effective plan of action for improvement and to ensure that graduates acquire the personal and professional attributes of an independent physician. We revised our evaluation policy and instituted a remediation protocol in 2008. | We revised our evaluation policy and instituted a remediation protocol in 2008. | Since that time, 130 pediatric anaesthesia fellows have graduated. Seven fellows (5%) underwent departmental formal consultation for deficient behavior or poor performance. Of these 7 fellows, 4 underwent an individualized remediation program (IRP). A formal performance review and written contract, with specifically identified problems and general themes, recommendations for time-based successful behaviors, and clearly identified consequences for unsuccessful behaviors, was initiated for each fellow undergoing an IRP. | Our approach has the advantage of multimodality, time-based daily evaluations, and group discussions in the context of a Clinical Competency Committee. Utilization of an IRP as a metric for progress has features similar to effective cognitive behavioral therapy contracts and has ensured that our graduates are held to clearly delineated and specified skills and behaviors that allow them to work independently in the field of pediatric anaesthesiology. |
| A stitch in time saves nine: intervention strategies for the remediation of competency | Stubbe et al. | 2007 | Descriptive | NA | NA | NA | The authors describe intervention strategies for the remediation of competency. | Themes included the identification of residents in need of remediation, process and monitoring of a remediation plan and remediation of attitudes. | The areas that are ripe for research and development include effective curricula, effective evaluation and remediation of competency, and leadership skill development. As medicine becomes more complex, so does the task of training physicians. Joint monitoring and outcomes research of the training enterprise are the scholarly and academic missions of the present and the future. |
| Remediation: where does the responsibility lie? | Swanwick & Whiteman | 2013 | Descriptive | NA | NA | NA | Inevitably, the evolving system has led to questions about how those that are falling short of the required professional standards can be helped to meet them. In other words, what in the way of remediation needs to be made available, how can it be provided efficiently and effectively, and where does the responsibility for such provision lie? | NA | So, to manage remediation locally requires expert input and placement support. How should this be provided? There are a number of options, ranging from the direct contracting of employers with private providers to the commissioning of a ‘regional’ professional support service, either privately provided or established within the NHS structures. |
| Mentoring for doctors in difficulty | Taylor et al. | 2012 | Descriptive | NA | NA | NA | The Mentoring Scheme for Doctors in Difficulty in the West Midlands aims to support any GP, including  GP trainees, experiencing significant professional problems by offering peer support from trained mentors.  The scheme was started in 2007 and has supported 47 mentees to date. Based on this experience,  and our ongoing research, we explore the potential for development of this scheme and for similar  schemes elsewhere. We discuss the originality of the West Midlands scheme, reflect on the successes and challenges so far  and on the impact of the scheme on doctors in difficulty and ultimately patient care in the region. | The West Midlands scheme has had many successes and continues to face financial challenges, but  positive feedback from mentees, demonstrating the impact of the mentoring process, has highlighted the  real value of this initiative. | Currently the West Midlands mentoring scheme provides a unique service to doctors in difficulty. Other  regions which share the aim of ensuring high-quality, safe care for patients in the region by supporting  doctors in difficulty may likewise wish to develop a scheme which aligns with the goals of primary care  education in general. The key feature is providing a local confidential mentoring service run by practising  GPs who have been trained in mentoring and are supported in their mentoring activity. There is potential  for larger schemes to support doctors in specialties other than general practice. |
| Self vs. other focus: predicting professionalism remediation  of emergency medicine residents | Thaxton et al. | 2018 | Qualitative | NA | 8 | We conducted a professional development quality improvement (QI) initiative to improve resident education and mentorship by achieving a better understanding of each resident’s reasons for valuing a career in medicine. This initiative entailed an interview administered to each resident beginning emergency medicine training at San Antonio Military Medical Center during 2006-2013. The interviews uniformly began with the standardized question “Why is Medicine important to you?” The residency program director documented a free-text summary of each response to this question, the accuracy of which was confirmed by the resident. We analyzed the text of each resident’s response after a review of the QI data suggested an association between responses and professionalism actions (retrospective cohort design). Two associate investigators blinded to all interview data, remedial actions, and resident identities categorized each text response as either self-focused (e.g., “I enjoy the challenge”) or other-focused (e.g., “I enjoy helping patients”). Additional de-identified data collected included demographics, and expressed personal importance of politics and religion. The primary outcome was a Clinical Competency Committee professionalism remedial action. | Our objective was to correlate the outcome of professionalism-related remedial actions during residency with the predictor variable of resident response to a standardized interview question: “Why is Medicine important to you?” | Of 114 physicians starting residency during 2006-2013, 106 (93.0%) completed the interview. There was good inter-rater reliability in associate investigator categorization of resident responses as either self-focused or other-focused (kappa coefficient 0.85). Thirteen of 50 residents (26.0%) expressed self-focus versus three of 54 (5.4%) residents expressed other-focus experienced professionalism remedial actions (p<0.01). This association held in a logistic regression model controlling for measured confounders (p=0.02). | Future work might study the impact on resident professionalism of other factors not explored in this study including social upbringing, childhood education, hospital environment, and the “hidden curriculum.”24,25 It would also be interesting to administer our interview tool in different contexts. In particular, future studies could examine whether simply educating residents about the association we identified in this study to stimulate their own introspection into the motivations underlying their career choices might be effective in decreasing instances of unprofessional behavior. Self-focused professional values as expressed during our standardized interview correlated with professionalism remedial actions during residency. These results may aid medical educators in identifying residents who may benefit from additional attention to professional development. |
| Remediation of underperformance in surgical trainees: a scoping review | To et al. | 2020 | Descriptive | NA | NA | Following scoping review protocols, we set out to identify the evidence-base for remediation of surgical trainees, outline key concepts and uncover areas to stimulate further research. | Surgical trainees with significant under-performance require formal support to return to an expected standard, termed remediation. The aim of this scoping review was to define remediation interventions, approaches, and contexts. | From a screen of 80 articles, 24 reported on remediation of surgical trainees. Most were from medical journals (n = 21, 88%) and published in the United States (n = 20, 83%). Ten articles (41%) reported outcomes of remediation of a trainee cohort and 7 (19%) were survey reports from surgical directors. The remainder were a mix of commentaries, editorials or reviews. Thirteen articles (54%) described trainees with deficiencies in multiple competencies, 8 articles (33%) had a singular focus on academic performance, and 1 article (3%) on technical skills. All articles used targeted individualized remediation strategies, a range of intervention methods (some multimodal) and recommended a 6- to 12-month period of remediation (n = 7, 29%). The program director was often the only supervisor (n = 12, 50%). One article reported trainees’ perspective of the process and one used educational theory to inform remediation. | Data with clearly reported outcomes were limited, but we found that targeted, individualized, multimodal and long-term remediation covering a range of competencies have been reported in the literature for surgical trainees. There is a need for development of explicit frameworks, strengthen the support for supervisors and trainees and further apply educational theory to develop better interventions that remediate deficiencies for all competencies. |
| Remediation practices for surgery residents | Torbeck & Canal | 2009 | Quantitative | 10 | NA | A web-based survey was e-mailed to 253 program directors of all US surgery residency programs. Questions were asked about remediation and probation practices for residents failing to meet the competencies. | This study sought to determine to what extent surgery programs are remediating residents who fail to achieve competency and to offer remediation strategies for those who struggle with problem residents. | Programs seem to struggle the least with knowing how to remediate medical knowledge and patient care deficits and struggle more with professionalism and interpersonal communication skills. Most programs have no remediation methods in place for systems-based practice and practice based learning and improvement deficits | For future study, it would be interesting to discover how many residents each program has terminated and/or failed to promote as a result of ACGME competency non-performance. It will be interesting to observe how the Residency Review Committee will deal with these challenges especially as it relates to the fluid dynamics of general surgery residencies of the future. |
| Competence and cognitive difficulty in physicians: a follow-up study. | Turnbull et al. | 2006 | Quantitative | 12 | NA | Between 1997 and 2001, the authors undertook neuropsychological screening of 45 participants of a physician competency assessment program. For those physicians reassessed after a period of remediation, the authors relate the findings of the physicians’ competence reassessments to their  neuropsychological scores. | Remediation of incompetent physicians has proven difficult and sometimes impossible. The authors wished to determine whether such physicians had neuropsychological impairment sufficient to explain their incompetence and their failure to improve after remedial continuing medical education (CME). | Nearly all physicians performing well on  competency assessment had no or mild  cognitive impairment. Conversely, a  significant number of physicians  performing poorly on competency  assessment had sufficient  neuropsychological difficulty to explain  their poor performance. The cognitive  impairment was more marked in elderly  physicians, and referencing the  neuropsychological scores to an agematched normative population  underestimates the impairment. No  physician with moderate or severe neuropsychological dysfunction had  successful competency reassessment.  Increasing age was associated with poor  performance on competency testing, but  was less strongly associated with  unsuccessful reassessment. | A large minority of the physicians who  fell significantly below desired levels of  competence had cognitive impairment  sufficient to explain their lack of  competence and their failure to improve  with remedial CME. |
| Remediation through transformation: applying educational theory to the struggling resident | Vipler et al. | 2020 | Descriptive | NA | NA | NA | The authors provide a descriptive overview of each of Cranton’s perspectives, introducing concrete examples drawn from the medical literature. This article will contrast current remediation strategies with those using TL theory in order to assist graduate medical educators in applying these principles to the remediation of their own struggling residents. | Current best practices for remediating struggling medical learners involve technical, step-by-step methods, often addressing one deficit at a time. While we are not advocating for a complete jettison of these well-established remediation strategies, we believe these are technical frameworks that, in isolation, may not work for the adaptive challenges facing the struggling resident. The strong emotions inherent to TL experiences may have great potential for motivation and may be used in tandem with some current remediation strategies to optimize success. Ultimately, through its three foundational perspectives, transformative learning presents a promising framework to guide medical educators in creating the adaptive solutions that are needed in remediating our struggling residents. | First, a formal literature review of TL in GME would provide an important state of the science with respect to this methodology. Second, Providing detailed and practical guidelines for educators in the health professions about facilitating TL should be included in faculty development. Third, since there are no studies comparing initiatives that foster TL with other educational methods, meaningful evaluation of outcomes when remediating with TL is needed. These may include graduation rate, patient satisfaction scores, or ideally, clinical outcomes. |
| Focused board intervention (FBI): A remediation program for written board preparation and the medical knowledge core competency | Visconti et al. | 2013 | Quantitative | 11 | NA | All residents in 8 classes of an EM 1–3 program were assessed using the In-Training Examination. Residents enrolled in the Focused Board Intervention (FBI) remediation program based on an absolute score on the EM 3 examination of ,70% or a score more than 1 SD below the national mean on the EM 1 or 2 examination. Individualized education plans (IEPs) were created for residents in the FBI program, combining self-study audio review lectures with short-answer examinations. The association between first-time pass rate for the American Board of Emergency Medicine (ABEM) Written Qualifying Examination (WQE) and completion of all IEPs was examined using the x2 test. | To determine whether a remediation program for residents identified as at risk for failure on the Emergency Medicine (EM) Written Board Examination is associated with improved outcomes. | Of the 64 residents graduating and sitting for the ABEM examination between 2000 and 2008, 26 (41%) were eligible for the program. Of these, 10 (38%) residents were compliant and had a first-time pass rate of 100%. The control group (12 residents who matched criteria but graduated before the FBI program was in place and 4 who were enrolled but failed to complete the program) had a 44% pass rate (7 of 16), which was significantly lower (x2 5 8.6, P 5.003). | When a program identifies a resident as falling behind in a basic competency, the implementation of a remedial education plan may correct this deficiency and help the resident find success. |
| What does remediation and probation status mean? a survey of emergency medicine residency program directors | Weizberg et al. | 2015 | Quantitative | 8 | NA | An anonymous electronic survey was distributed to EM PDs via e-mail using SurveyMonkey to determine the current practice followed after residents are placed on remediation or probation. The survey queried four designations: informal remediation, formal remediation, informal probation, and formal  probation. These designations were compared for deficits in the domains of medical knowledge (MK) and non-MK remediation. The survey asked what process for designation exists and what actions are triggered, specifically if documentation is placed in a resident’s file, if the graduate medical education (GME) office is notified, if faculty are informed, or if resident privileges are limited. Descriptive data are reported. | To determine if EM PDs follow a common process to guide actions when residents are placed on remediation and probation. | There was great variation among PDs in the management and definition of remediation and probation. Between 81 and 86% of programs place an official letter into the resident’s file regarding formal remediation and probation. However, only about 50% notify the GME office when a resident is placed on formal remediation. There were no statistical differences between MK and non-MK remediation practices. | Creation of a standardized practice of when and how to execute remediation and probation would guide PDs in the assessment of the academic and clinical performance of trainees. It would also assist future employers in their understanding of a resident’s training that included a remediation period. Thus, successfully completing a remediation period need not hold a negative connotation. Rather, it would reassure an employer that an identified deficiency was corrected and brought up  to an acceptable standard. A standardized process would provide a clear definition and process of management of performance problems. This would provide clarity for the resident, the program, the GME office, and other stakeholders. |
| Remediation with trust, assurance and safety | Whiteman & Jamieson | 2007 | Descriptive | NA | NA | NA | So how should you set up a service that is going to offer meaningful remediation  to a general practitioner (GP) identified as poorly performing? How do you pick them up from the damaging confusion that results from most performance  assessment processes and help them construct a way forward? How do you work  collaboratively with the other stakeholders and regulatory organisations to  ensure that remediation requirements are fair and patient safety is not compromised? | Analysis of the caseload has shown that we have been asked to intervene at  many differing stages in cases, ranging from ‘light-touch’ work-based support  and supervision for practitioners still working in their practices to the provision  of comprehensive remedial retraining for practitioners required to work in a  closely supervised and monitored environment. | 1 Patient safety. The overriding principle behind our approach to remediation and  support. With every practitioner we see we now ask ourselves whether there are  patient safety issues being exposed by the remediation work we are involved with.  We do not assume that the referring body will have been fully reassured that none  existed nor that none would be exposed by retraining and remedial support work.  Not least because through endeavouring to engage with the practitioner we often  uncover further concerns about their fitness to practise and their abilities to  engage with an educational programme.  2 Using the strengths of deanery resources, including our network of experienced  educators to support those referred in their learning plans and career development  and drawing on the supervision and support of colleagues to help clarify  thinking and boundaries around case management.  3 Applying adult learning principles and methods through empowerment of the  practitioner, and facilitation of their learning progress, often a technique that  those GPs referred to us have not had experience of.  4 Collaborative working with organisations involved with regulation and assessment  in individual case management and the development of local procedures  and expertise in performance cases. |
| Adverse childhood experiences in trainees and physicians with professionalism lapses: implications for medical education and remediation | Williams et al. | 2021 | Quantitative | 14 | NA | A final sample of 123 cases of U.S. trainees and physicians who had been referred to a Midwestern center for assessment and/or remediation of professionalism issues from 2013 to 2018 was created. Included professionalism lapses fell within 3 categories: boundary violation, disruptive behavior, or potential substance use disorder concerns. All participants completed a psychosocial developmental interview, which includes questions about ACE exposure. Overall rate of reported ACEs and types of ACEs reported were explored. | Unprofessional behavior, which can include failure to engage, dishonest and/or disrespectful behavior, and poor self-awareness, can be demonstrated by medical trainees and practicing physicians. In the authors’ experience, these types of behaviors are associated with exposure to adverse childhood experiences (ACEs). Given this overlap, the authors studied the percentage of ACEs among trainees and physicians referred for fitness-for-duty evaluations and patterns between the types of ACEs experienced and the reason for referral. | Eighty-six (70%) participants reported at least 1 ACE, while 27 (22%) reported 4 or more. Compared with national data, these results show significantly higher occurrence rates of 1 or more ACEs and a lower occurrence rate of 0 ACEs. ACEs that predicted reasons for referral were physical or sexual abuse, feeling unwanted or unloved, witnessing abuse of their mother or stepmother, or caretaker substance use. | Adoption of a trauma-informed medical education approach may help those that have been impacted by trauma rebuild a sense of control and empowerment. The findings of this study may be useful predictors in identifying those at risk of problematic behavior and recidivism before a sentinel event. |
| The problem resident behavior guide: strategies for remediation | Williamson, Quattromani, & Aldeen | 2016 | Review | NA | NA | The study authors, who have professional experience at three different academic hospitals with emergency medicine residency programs created ‘‘The Problem Resident Behavior Guide’’, an instructional manual that provides more than 200 objective and achievable strategies for remediation based on deficiencies identified within the framework of the EM milestones of the ACGME core competencies. The remediation strategies stem from the experiences of the three study authors supplemented by recommendations from additional education leaders. The authors also conducted an extensive Pubmed literature search on the topic of remediation, and incorporated recommendations from the internal medicine, critical care, surgery and family practice literature on the subject of remediation. | The goal of the ‘‘Problem Resident Behavior Guide’’ is to provide specific strategies for resident remediation based on deficiencies identified within the framework of the EM milestones. The ‘‘Problem Resident Behavior Guide’’ is a written instructional manual that provides concrete examples of remediation strategies to address specific milestone deficiencies | Most recommendations require active participation by the resident with guidance by faculty to achieve the remediation expectations. The ACGME outcomes-based milestones aid in the identification of deficiencies with regards to resident performance without providing recommendations on remediation. The Problem Resident Behavior Guide can therefore have a significant impact by filling in this gap. | Emergency medicine residency programs must comply with the new ACGME milestones. Once a deficiency is identified, we hope this guide will provide helpful starting points in the remediation process. Each recommendation can be tailored to suit the needs and culture of individual residencies. We welcome all feedback and suggestions for future editions of this guide. |
| Resident evaluation and remediation:  a comprehensive approach | Wu et al. | 2010 | Interventional | NA | NA | Our remediation program uses resources from both our radiology department and our institutional GME office similar to other programs and specialties.6–9 The centrepiece is a resident/program director/faculty educational liaison agreement form that documents the problem and its significance and outlines a detailed remediation plan that FIGURE Evaluation and Remediation Diagram shows the relationship between evaluation and remediation. Reprinted with permission from Boiselle.2 PRACTICAL ARTICLES Journal of Graduate Medical Education, June 2010 243 Journal of Graduate Medical Education 2010.2:242-245. Downloaded from www.jgme.org by WDAS Country Access Consortium on 01/12/19. For personal use only. contains clear goals and benchmarks, as well as a plan for reassessment to determine whether the remediation program has been successful. This intervention was designed in accordance with the principles outlined by Borus.10 A discernible strength of this framework is that it sets up an alliance between 3 parties: (1) the resident, (2) the program director, and (3) the faculty educational liaison with experience in the specific area of difficulty. With all 3 parties invested in the agreement, the ‘‘intervention’’ feels like a concerted team effort rather than a punitive situation and frames the intervention itself in positive rather than oppositional terms. A proactive remediation plan may include additional reading assignments, one-on-one faculty/ mentor tutorials, specific assistance with reporting or procedural skills, and repeating a clinical rotation after completion of such measures. | In recognition of the importance of early detection and prompt remediation of residents who struggle with academic performance, we sought to develop a multifaceted approach to resident evaluation with the aim of early identification and prompt remediation of difficulties. This article describes our comprehensive evaluation program and remediation program, which uses resources within our radiology department and institutional graduate medical education (GME) office. | NA | In summary, a comprehensive resident evaluation and remediation program is an essential component of any residency program. Having an evaluation program with a multifaceted approach has the potential to detect problems early, when they are potentially more responsive to remediation. Moreover, a proactive remediation program that effectively identifies performance issues and incorporates departmental and institutional resources is necessary to effectively deal with issues once they are recognized. |
| General surgery resident remediation and attrition | Yaghoubian et al. | 2012 | Quantitative | 14 | NA | Eleven-year retrospective analysis of 348 categorical general surgery residents at 6 West Coast programs. | To determine the rates and predictors of remediation and attrition among general surgery residents. | Three hundred forty-eight categorical general surgery residents were included. One hundred seven residents (31%) required remediation, of which 27 were remediated more than once. Fifty-five residents (15.8%) left their programs, although only 2 were owing to failed remediation. Remediation was not a predictor of attrition (20% attrition for those remediated vs 15% who were not [P = .40]). Remediation was most frequently initiated owing to a deficiency in medical knowledge (74%). Remediation consisted of monthly meetings with faculty (79%), reading assignments (72%), required conferences (27%), therapy (12%), and repeating a clinical year (6.5%). On univariate analysis, predictors of remediation included receiving honours in the third-year surgery clerkship, United States Medical Licensing Examination (USMLE) step 1 and/or step 2, and American Board of Surgery In-Training Examination scores at postgraduate years 1 through 4. On multivariable regression analysis, remediation was associated with receiving honors in surgery (odds ratio, 1.9; P = .01) and USMLE step 1 score (odds ratio, 0.9; P = .02). On univariate analysis, the only predictor of attrition was the American Board of Surgery In-Training Examination score at the postgraduate year 3 level (P = .04). | Almost one third of categorical general surgery residents required remediation during residency, which was most often owing to medical knowledge deficits. Lower USMLE step 1 scores were predictors of the need for remediation. Most remediated residents successfully completed the program. Given the high rates of remediation and the increased educational burden on clinical faculty, medical schools need to focus on better preparing students to enter surgical residency. |
| Remediation of residents in difficulty: a retrospective 10-year review of the experience of a postgraduate board of examiners | Zbieranowski et al. | 2013 | Quantitative | 7.5 | NA | A retrospective review of resident records for the University of Toronto Faculty of Medicine's (UT-FOM) Board of Examiners for Postgraduate Programs (BOE-PG) was done from July 1, 1999 to June 30, 2009 using predetermined data elements entered into a standardized form and analyzed for trends and significance. Outcomes for residents in difficulty were tracked through university registration systems and licensure databases. | To determine, through a 10-year review, (1) the prevalence of residents in difficulty, (2) characteristics of these residents, (3) areas of residents' weakness, and (4) outcomes of residents who undergo remediation. | During 10 years, 103 UT-FOM residents were referred to the BOE-PG, representing 3% of all residents enrolled. The annual prevalence of residents referred to the BOE-PG ranged from 0.2% to 1.5%. The CanMEDS framework was used to classify areas of residents' weaknesses and organize remediation plans. All 100 residents studied had either medical expertise (85%) or professionalism (15%) weaknesses or both. Residents had difficulties with an average of 2.6 CanMEDS Roles, with highest frequencies of Medical Expert (85%) Professional (51%), Communicator (49%), Manager (43%), and Collaborator (20%). Often, there were multiple remediation periods, with an average of six months' duration. Usually, remediation was successful; 78% completed residency education, 17% were unsuccessful, and 5% remained in training. | Our study prompted questions that require further research. Currently, there are almost no data that reveal how physicians who were in remediation during their studies succeed in professional practice after residency.16 Are they confident and competent physicians? Do they later run into problems professionally? This information may be difficult to obtain but would be useful for outcome measurements on current practice. It would be interesting to determine whether there is a difference in the prevalence of residents in difficulty between those who are international medical graduates and those who are Canadian-trained physicians and whether weaknesses in the CanMEDS competencies are the same for both groups. More important, it would be beneficial to know what program directors and faculty can do to improve remediation processes. Finally, a question that requires further inquiry is whether earlier referral to the BOE-PG results in a more effective remediation strategy, as defined by successful completion of residency education, a subject that we plan to address in a further study. |
